# HLA-DRB1: A new potential prognostic factor and therapeutic target of cutaneous melanoma and an indicator of tumor microenvironment remodeling

**DOI:** 10.1371/journal.pone.0274897

**Published:** 2022-09-21

**Authors:** Huiling Deng, Yuxuan Chen, Jiecong Wang, Ran An

**Affiliations:** Department of Plastic Surgery, Union Hospital, Tongji Medical College, Huazhong University of Science and Technology, Wuhan, PR China; Centro de Investigacion y de Estudios Avanzados del Instituto Politecnico Nacional, MEXICO

## Abstract

Cutaneous melanoma (CM) is the most common skin cancer and one of the most aggressive cancers and its incidence has risen dramatically over the past few decades. The tumor microenvironment (TME) plays a crucial role in the occurrence and development of cutaneous melanoma. Nevertheless, the dynamics modulation of the immune and stromal components in the TME is not fully understood. In this study, 471 CM samples were obtained from TCGA database, and the ratio of tumor-infiltrating immune cells (TICs) in the TME were estimated using the ESTIMATE algorithms and CIBERSORT computational method. The differently expressed genes (DEGs) were applied to GO and KEGG function enrichment analysis, establishment of protein-protein interaction (PPI) network and univariate Cox regression analysis. Subsequently, we identified a predictive factor: HLA-DRB1 (major histocompatibility complex, class II, DR beta 1) by the intersection analysis of the hub genes of PPI network and the genes associated with the prognosis of the CM patients obtained by univariate Cox regression analysis. Correlation analysis and survival analysis showed that the expression level of HLA-DRB1 was negatively correlated with the Stage of the patients while positively correlated with the survival, prognosis and TME of melanoma. The GEPIA web server and the representative immunohistochemical images of HLA-DRB1 in the normal skin tissue and melanoma tissue from the Human Protein Atlas (HPA) database were applied to validate the expression level of HLA-DRB1. CIBERSORT analysis for the ratio of TICs indicated that 9 types of TICs were positively correlated with the expression level of HLA-DRB1 and only 4 types of TICs were negatively correlated with the expression level of HLA-DRB1. These results suggested that the expression level of HLA-DRB1 may be related to the immune activity of the TME and may affect the prognosis of CM patients by changing the status of the TME.

## Introduction

Cutaneous melanoma (CM) is a highly aggressive malignancy caused by the malignant transformation of melanocytes and it is also the most common type of skin cancer [1–3]. According to the latest population-based cancer occurrence data released by the American Cancer Society, 100,350 new cutaneous melanoma cases and 6850 cutaneous melanoma deaths are expected in the United States in 2020, 4% increase in new cases and 5% decrease in deaths compared to 2019 [4, 5]. The known risk factors for cutaneous melanoma include ultraviolet radiation, indoor tanning, dysplastic naevi, genetic factor, phenotypic features and so on [6]. Current therapeutic approaches include surgical therapy, radiotherapy, chemotherapy, immunotherapy, targeted therapy and so on [7]. Surgical excision is the primary treatment of cutaneous melanoma. In the early stages of CM, it can be successfully treated with simple surgery and has a high survival rate, but once it has spread and metastasized, it can quickly become a life-threatening disease [1, 6, 8]. Hence, early diagnosis is of great significance for the prognosis of CM patients. In addition, it is necessary to find new biomarkers and drug therapy targets for the sake of improving the accuracy of diagnosis and treatment. Multiple studies have revealed that genetic mutations and the alterations of TME led to the spread of melanoma [9–13]. With the rapid development of high-throughput sequencing technologies, it has become more convenient to detect gene expression during tumor progression, providing effective targets for diagnosis and treatment, but there is still a lack of reliable biomarkers to monitor treatment effects [3]. Consequently, there is an urgent need to find microenvironment-related genes that affect the prognosis of CM patients.

The tumor microenvironment (TME) is a heterogeneous population of cells comprised of diverse immune cells, tumor cells and stromal cells [14, 15]. Accumulated studies have illuminated that the TME plays an important role in tumor occurrence and predicting clinical prognosis of the CM patients [16, 17]. With the use of targeted therapy and immune checkpoint inhibitors, both recurrence-free survival and overall survival in high-risk cutaneous melanoma (stage IIB/C-III) patients have improved. However, due to the presence of primary resistance, not all patients respond to this treatment which can also lead to severe toxicity and acquired resistance [18–21]. Accordingly, finding out the causes of primary and acquired drug resistance is of great significance for improving the survival rate and prognosis of CM patients. It has been demonstrated that the intrinsic factors which lead to immunotherapy resistance included the expression of certain genes and pathways in tumor cell can prevent the invasion or function of immune cells in the TME [20]. A large number of researches have shown that TME played a dual role in the occurrence and progression of tumors. Changes in the TME can not only promote normalization of tumor cells, but also promote tumor growth, invasion and metastasis [22]. However, the dynamic regulatory processes and mechanisms of various molecular components in the TME are not fully understood. Therefore, it is of great importance to understand the molecular composition and function of TME for promoting valid diagnosis, immunotherapy and prognosis of CM patients.

In this study, 471 CM samples were obtained from the TCGA database. Furthermore, ESTIMATE algorithms and CIBERSORT computational method were applied to estimate the ratio of tumor-infiltrating immune cell (TIC) and the proportion of immune cell and stromal cell and identified a predictive factor, HLA-DRB1(major histocompatibility complex, class II, DR beta 1), which may be a latent indicator for reflecting the change of TME status in CM was identified. The HLA system is the major histocompatibility complex in humans. It is the most complex and polymorphic gene in human beings, which is related to the development of various tumors, and has always been the focus of tumor immunity research [23–25]. The HLA class II molecules are composed of an α chain (DRA) and a β chain (DRB) and are usually expressed by immune cell subsets, including B cells, activated T cells, dendritic cells, macrophages and so on [26]. HLA molecules play a primary role in the activation of the immune cell and autoimmune diseases by presenting antigenic peptides to T cells [27]. Accumulating evidences have revealed that the HLA system is associated with many tumors, including cervical cancer, gastric cancer, breast cancer and so on [28–32].

## Materials and methods

### Data source

The transcriptome profiling and clinical data of 471 CM samples (including one normal sample and 470 CM samples) analyzed in this study were obtained from The Cancer Genome Atlas (TCGA) database (https://portal.gdc.cancer.gov/). The data was normalized by fragments per kilobase per million (FPKM) for subsequent analysis. Subsequently, integrity screening was performed to exclude samples without complete clinical information and expression information. Finally, only 406 samples with complete clinical data were used for subsequent analysis. The progression-free survival (PFS) data of CM patients were obtained from the UCSC Xena database (http://xena.ucsc.edu) and only 400 of the 406 samples included in this study owned complete PFS data.

### Generation of ImmuneScore, StromalScore and ESTIMATEScore

ESTIMATE algorithm, which was realized by the estimate package of the R language version 4.1.0, was applied to estimate the proportions of the immune-stromal components in the TME of each sample. ESTIMATE is a tool for predicting tumors, the presence of infiltrating stromal cells or immune cells in tumor tissues using the gene expression data. The algorithm includes the ImmuneScore (representing the infiltration of immune cells in tumor tissue), StromalScore (capturing the presence of stroma in tumor tissue), and ESTIMATEScore (inferring the purity of tumor), which were positively relevant to the ratio of immune components, stromal component and the sum of immune and stromal component, respectively. A higher score means a higher ratio in the TME.

### Survival analysis and clinical correlation analysis of the TME

Survival analysis was implemented through the survival package and survminer package of R language. Kaplan-Merier method was applied to plot the survival curve, and the significance test was realized by log rank. The correlation analyses between the TME (ImmuneScore, StromalScore, and ESTIMATEScore) and clinical features (Age, Stage, and Ulceration) were achieved through package limma and package ggpubr of the R language. The correlation tests were achieved through the Wilcoxon rank sum test and the Kruskal-Walls rank sum test, p-value <0.05 was regarded as significant correlation.

### Generation of DEGs

406 CM samples were segmented into high score group and low score group based on the median value of StromalScore. Wilcoxon rank sum test and the package limma of R language were applied to conduct the differentiation analysis. Genes that met log FC > 0 and false discovery rate (FDR) <0.05 were considered as Stromal differential genes. Similarly, the samples were divided into high score group and low score group according to the median value of ImmuneScore. And then, Immune differential genes were identified by the same method. Finally, the intersection analysis of Stromal differential genes and Immune differential genes was conducted to obtain the differently expressed genes (DEGs) which were used for subsequent analyses. The intersection analysis of Stromal differential genes and Immune differential genes was accomplished by the package VennDiagram of the R language.

### Functional enrichment analysis of DEGs

Gene Ontology (GO) analysis is frequently used in functional enrichment studies. Moreover, the enriched GO terms can be categorized into biological process (BP), molecular function (MF), and cellular component (CC). The Kyoto Encyclopedia of Genes and Genomes (KEGG) is a database designed to systematically analyze the metabolic pathways and functions of gene products in cells. GO and KEGG enrichment analysis of the DEGs in this study were accomplished by the colorspace, stringi, ggplot2, DOSE, clusterProfiler, enrichplot, and org.Hs.eg.db packages of the R language. Only terms that satisfied both p-value <0.05 and q-value <0.05 were deemed to be significant enrichment.

### PPI network establishment

The STRING database is one of several online resources dedicated to organism-wide protein association networks and it is designed to provide critical assessment and integration of protein-protein interactions, including physical interactions and functional associations [33, 34]. The PPI network of the DEGs was constructed through the STRING database (http://string-db.org). The DEGs were submitted to the STRING database to assess the latent protein-protein interaction relationship. Only nodes with the confidence of interactive relationship >0.9 will appear in the PPI network. Thereafter, the Cytoscape version 3.7.1 software was utilized to visualize the PPI network. Nodes with high connectivity degrees often played a more crucial role in maintaining the stability of the whole PPI network. CytoHubba, a plugin of Cytoscape, was applied to calculate the connectivity degree between each protein node. The genes with the top 20 connectivity degree were regarded as hub genes of the PPI network.

### Univariate Cox regression analysis

Univariate Cox regression analysis, which was realized by the survival package and limma package of the R language, was performed to explore the genes connected with the prognosis of CM patients. Only genes that satisfied p-value <0.000005 would appear in the forest map. Subsequently, the intersection analysis of the hub genes of PPI network and the genes associated with the prognosis of the CM patients obtained by univariate Cox regression analysis was implemented. And the intersection analysis was conducted by the Venn diagram webtool (bioinformatics.psb.ugent.be/webtools/Venn/).

### Survival analysis of HLA-DRB1 expression

The overall survival (OS) and progression-free survival (PFS) curves of HLA-DRB1 expression were accomplished by the survival package and survminer package of the R language. GEPIA (http://gepia.cancer-pku.cn/) is a web server that integrates the gene expression profile data from TCGA (https://portal.gdc.cancer.gov/) and GTEx (https://gtexportal.org) database. It can be used for cancer and normal gene expression profiling and interactive analysis, including differential expression, correlation analysis, survival analysis and so on [35]. The disease-free survival (DFS) curve of HLA-DRB1 expression was plotted through the GEPIA web server. All of the survival curves were plotted by Kaplan-meier method and the significance test was accomplished through log rank.

### Correlation analysis among the Stage, TME and HLA-DRB1 expression

The correlation analysis between the Stage and the expression level of HLA-DRB1 was realized by the limma package and ggpubr package of the R language. Correlation test was achieved through the Kruskal-Walls rank sum test, and p-value <0.05 was considered significant correlation. Moreover, Pearson correlation analysis, which was achieved through the software StataMP 16, was applied to evaluate the correlation between the TME (ImmnueScore, StromalScore, and ESTIMATEScore) and the expression level of HLA-DRB1. And Pearson correlation coefficient (R) >0 represented positive correlation while R<0 on behalf of negative correlation.

### ROC curves and independence of HLA-DRB1 expression

The IBM SPSS statistics 28.0.0.0 software was utilized to plot the ROC curve of HLA-DRB1so as to assess the accuracy of the HLA-DRB1 expression in predicting the prognosis and survival of the CM patients. The univariate and multivariate Cox regression analyses, which were achieved through the package survival, survminer of R language, were implemented to test whether the expression level of the HLA-DRB1 can be an independent prognostic factor of melanoma. The factor that met p-value <0.05 both in the univariate and multivariate prognostic analysis was deemed to be an independent prognostic factor of CM.

### Validation of the expression level of HLA-DRB1

The validation of the expression level of HLA-DRB1 in the normal and melanoma samples was realized by the GEPIA web server (http://gepia.cancer-pku.cn/). The |log_2_ FC| which was defined as medianTumor—medianNormal Cutoff of the gene expression box plot was 1. The log_2_ FC = medianTumor -medianNormal. The medianTumor and medianNormal in the formula respectively mean the median value of HLA-DRB1 expression in the CM group and Normal group and the HLA-DRB1 expression data were first log_2_ transformed. P-value Cutoff was 0.05 and other parameters were default values. The Human Protein Atlas (HPA) database (https://www.proteinatlas.org/) is a Swedish-based program initiated in 2003, this database contains 44 kinds of normal human tissue and 17 different forms of human cancer mRNA and protein expression data and immunohistochemical staining slice image information. All data in this database is open and freely accessible to explore the human proteome. The immunohistochemical images of the normal skin tissue and melanoma tissue were obtained from HPA database.

### TICs abundance profile of 406 CM samples

TIC abundance profile of the CM samples was estimated by CIBERSORT computational method and the corrplot package of the R language. Subsequently, quality filtering was carried out, and only samples with p-value <0.05 were employed for the later analysis.

### Differentiation and correlation analysis between HLA-DRB1 expression and the content of immune cells

The differentiation analysis between the expression of HLA-DRB1 and the content of immune cells was finished by limma package and vioplot package of the R language. Wilcoxon rank sum test was employed for statistical significance test. Only immune cells with p-value <0.05 were considered to be significantly different. The correlation analysis between the expression level of HLA-DRB1 expression and the content of immune cells was realized by the limma, ggExtra, ggpubr, and ggplot2 packages of the R language. Spearman rank correlation coefficient was applied for the correlation test. R-value >0 means positive correlation and R-value <0 means negative correlation. P-value <0.05 was deemed to be significantly correlated.

## Result

### Analysis process of this study

**Fig 1:**
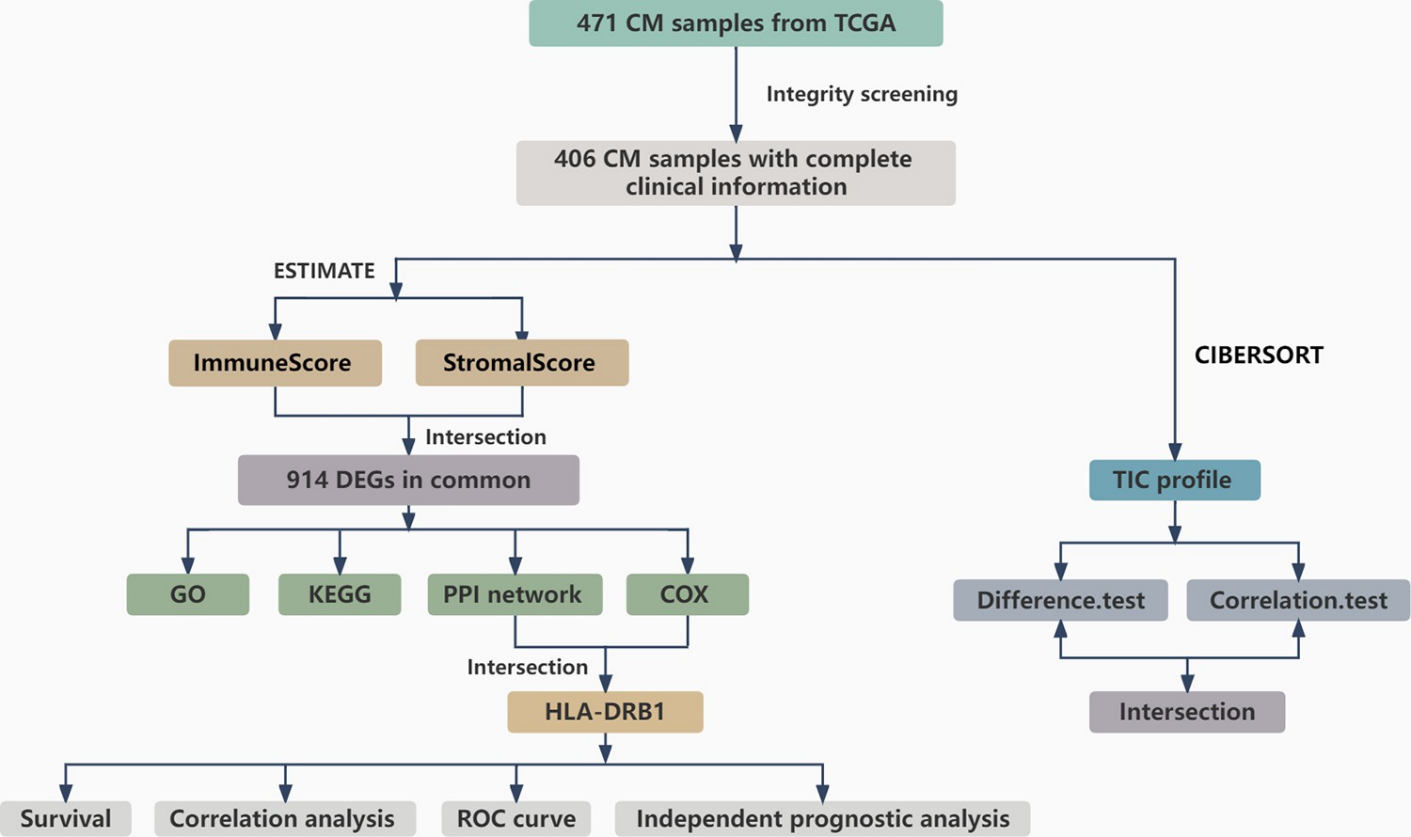
Analysis workflow of this study.

The workflow chart of our study was illustrated in Fig 1. The transcriptome expression profiles which were normalized by the fragments per kilobase per million (FPKM) and the clinical data of 471 CM samples were acquired from the TCGA database. The 406 CM samples with complete clinical information were utilized for subsequent analysis. ESTIMATE algorithms and CIBERSORT computational method were applied to evaluate the ratio of TICs and the proportion of immune cells and stromal cells. The 406 CM samples were respectively split into high score group and low score group on the basis of the median value of ImmuneScore, StromalScore, and ESTIMATEScore. Survival analysis was applied to explore the relationship between the ratio of ImmuneScore, StromalScore, ESTIMATEScore, and the survival of CM patients. And then correlation analyses between the TME and clinical features were performed. Subsequently, differentiation analysis of gene expression was proceeded to find DEGs. And the DEGs were used for GO annotation analysis, KEGG pathways enrichment analysis, univariate Cox regression analysis, and establishing protein-protein interaction (PPI) network. Consequently, HLA-DRB1 was identified by the intersection analysis between the top 20 hub genes obtained from the PPI network and the result of the univariate Cox regression analysis. Followed by survival analysis and correlation analysis among the TME, Stage, and the expression level of HLA-DRB1. And then ROC curve and independence prognostic analysis of HLA-DRB1 expression were conducted to assess the accuracy and independence of the expression level of HLA-DRB1 in predicting the survival and prognosis of the CM. Afterward, the TICs abundance profile of 406 CM samples was estimated by the CIBERSORT computational method. Whereafter, differentiation analysis and correlation analysis between HLA-DRB1 expression and the content of immune cells were performed. Eventually, intersection analysis was enforced using the result of differentiation analysis and correlation analysis.

### Survival analysis of the TME

**Fig 2:**
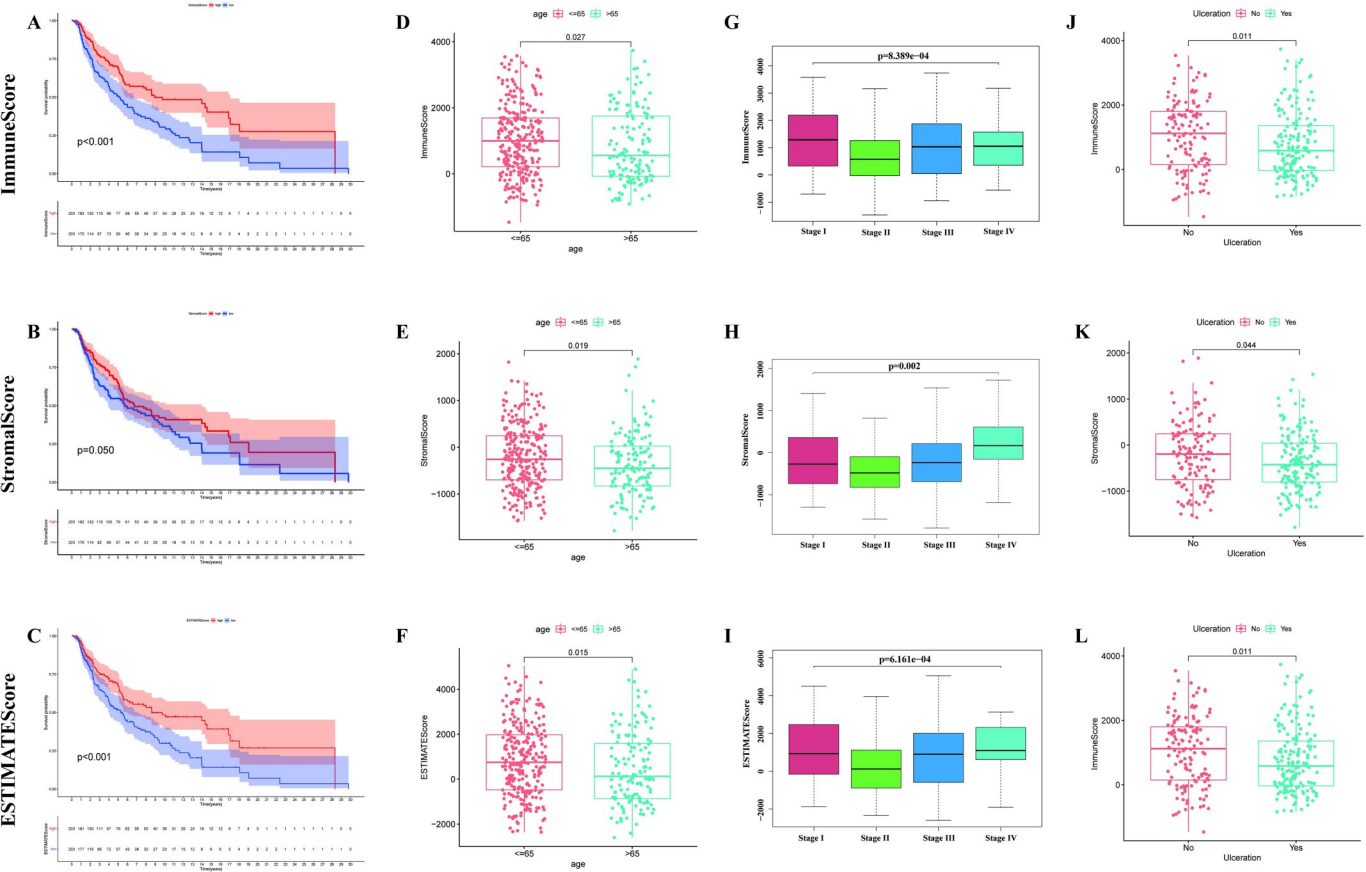
Survival curve and correlation analysis between TME and clinical features. (A) Kaplan-Meier survival analysis of ImmuneScore. (B) Survival analysis result of StromalScore. (C) Survival analysis of ESTIMATEScore. (D-L) Correlation analyses among ImmuneScore, StromalScore, and ESTIMATEScore and age, Stage, and Ulceration.

Kaplan-Merier method, which was based on the ImmuneScore, StromalScore, and ESTIMATEScore, was applied to research the relationship between the ratio of immune cells, stromal cells, and the survival probability of CM patients. The 406 CM samples were respectively segmented into high score group and low score group according to the median value of ImmuneScore, StromalScore, ESTIMATEScore. A higher score means a higher ratio in the TME. ESTIMATEScore is the sum of ImmuneScore and StromalScore. It can be seen from the Fig 2A that the patients with high ImmuneScore possessed a higher survival probability and survival time than the patients with low ImmuneScore. Besides, the median months of survival in the high ImmuneScore group was longer than that in low ImmunScore group. The high ImmuneScore group had a median survival of 43.8 months, while the low ImmuneScore group had a median survival of 27.3 months. Although StromalScore was not statistically significant with survival probability (Fig 2B), ESTIMATEScore was still positively correlated with the survival probability (Fig 2C). Those result suggested that the ratio of immune cells made a more important role in the survival of CM patients.

### Correlation analysis between the TME and clinical features

As is shown in Fig 2D–2F, the patients were divided into "< = 65" group and ">65" group according to their age. Analogously, ImmuneScore (Fig 2D), StromalScore (Fig 2E), and ESTIMATEScore (Fig 2F) all were negatively correlated with the age of patients. It can be known from Fig 2G–2I that there were significant differences in the ImmuneScore, StromalScore, and ESTIMATEScore among patients in different Stages. Furthermore, the patients in Stage I owned a higher ImmuneScore and ESTIMATEScore than the patients in other Stages. As is displayed in Fig 2J–2L, ImmuneScore, StromalScore, and ESTIMATEScore were significantly relevant to the presence or absence of ulceration as well, the patients without ulceration owned a higher score. It can be inferred from these results that TME might play an indispensable role in the invasion, metastasis, and progression of CM patients, but further exploration is still needed.

### Differentiation analysis of gene expression and generation of DEGs

**Fig 3:**
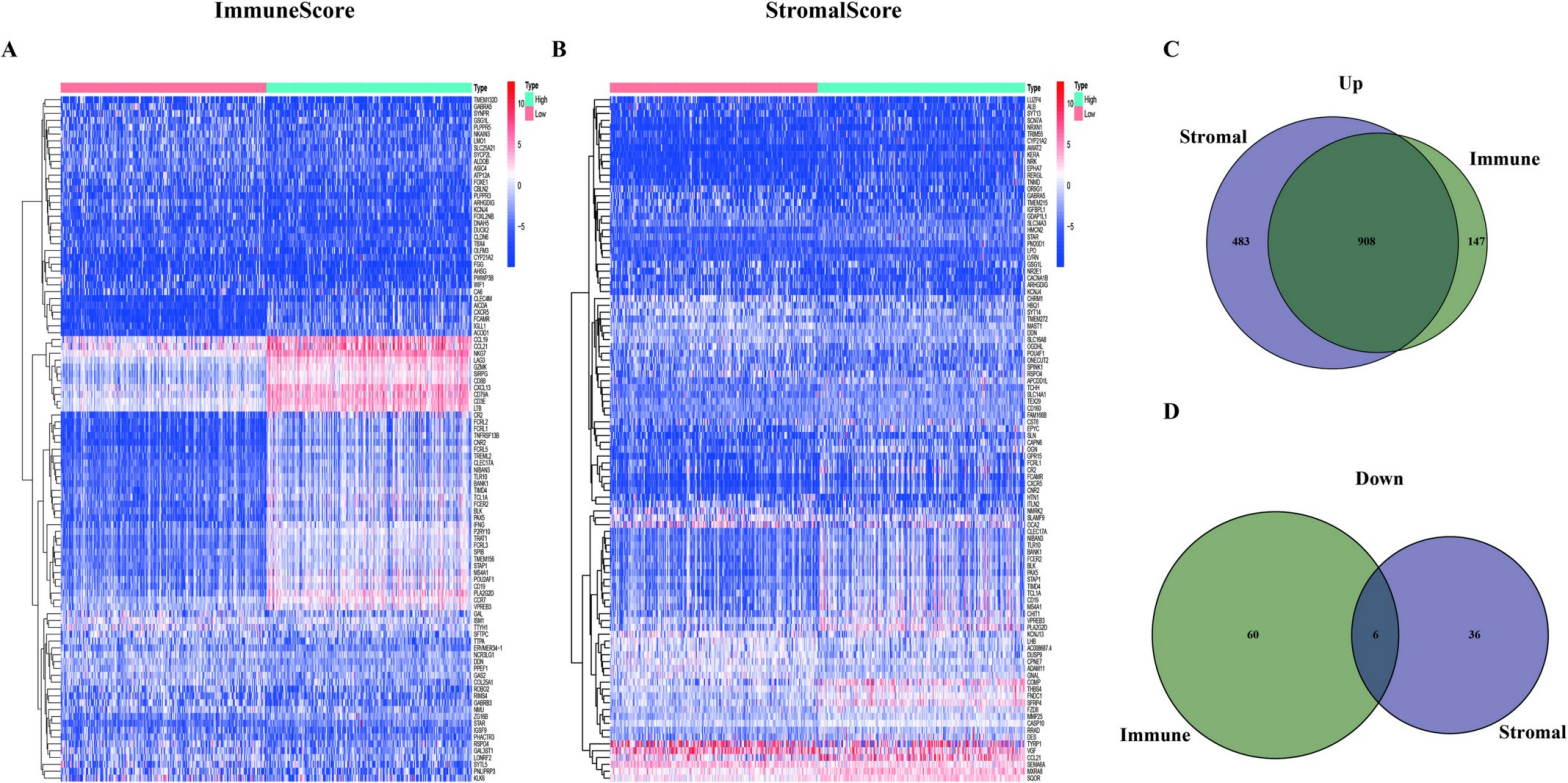
Heatmaps and Venn plots of DEGs. (A) Heatmap of Immune differential genes generated by the comparison between the high Immunescore group and the low Immunescore group. The row name of the heatmap is the name of genes and the column name is sample ID (not added in the figure). (B) Heatmap of Stromal differential genes. (C, D) Venn plots demonstrated the result of the intersection analysis of Immune differential genes and Stromal differential genes.

To research the exact changes of gene expression, differentiation analysis was respectively applied to ImmuneScore and StromalScore. The 406 CM samples were segmented into high score group and low score group on the basis of the median value of ImmuneScore (Fig 3A) and StromalScore (Fig 3B) to investigate the difference in gene expression between the high score group and low score group. And 1121 Immune differential genes (including 1055 up-regulated genes and 66 down-regulated genes) and 1433 Stromal differential genes (including 1391 up-regulated genes and 42 down-regulated genes) were identified. The intersection analysis of the Stromal differential genes and Immune differential genes was realized by the VennDiagram package of the R language. The genes in the overlapping part of the Venn plots (Fig 3C and 3D) were identified as DEGs. It can be seen from Fig 3C and 3D that there are 914 DEGs in total, including 908 up-regulated DEGs and 6 down-regulated DEGs. These DEGs (total 914 genes) might play a vital role in the progression of the CM patients.

### Functional enrichment analysis of DEGs

**Fig 4:**
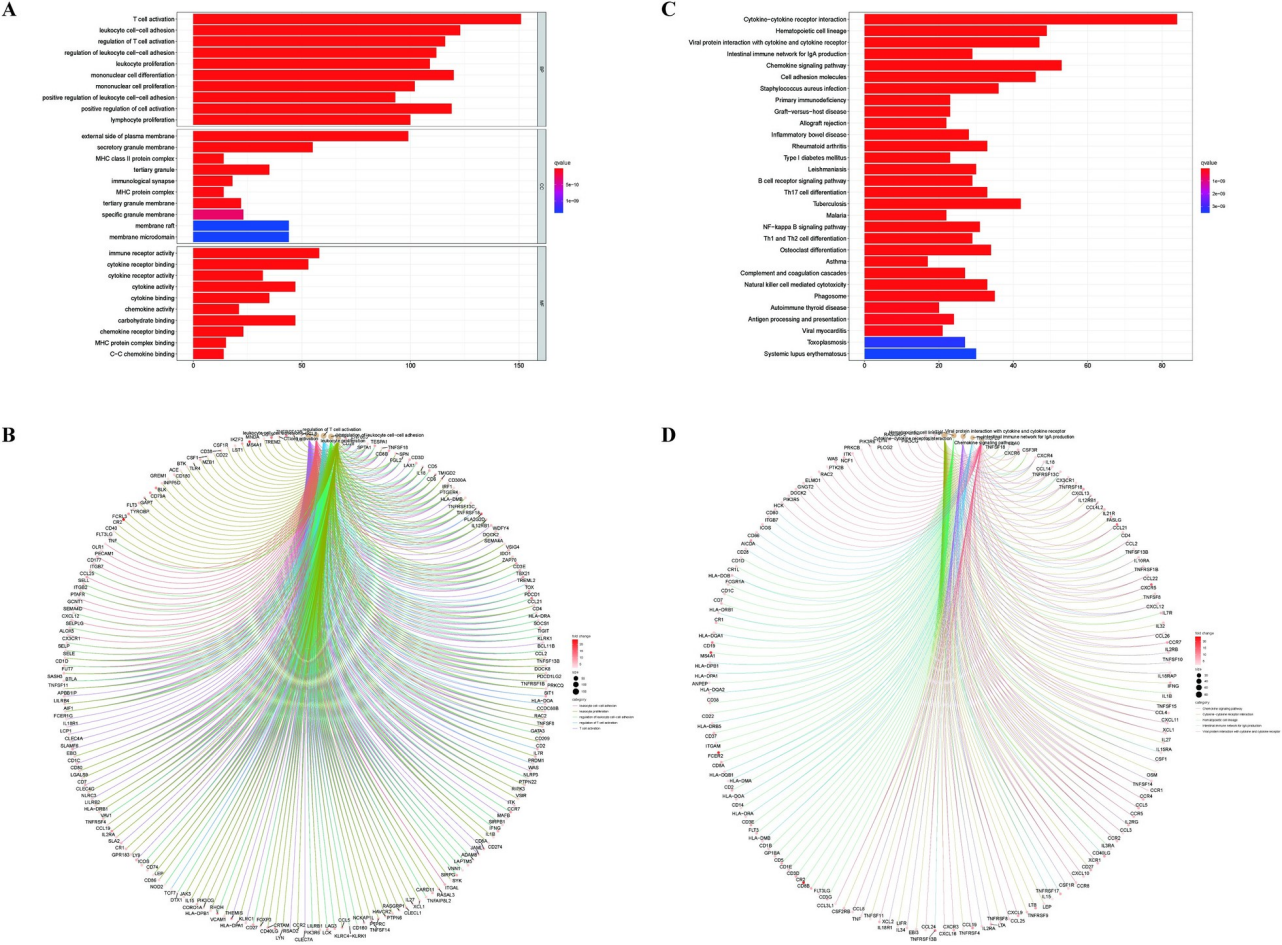
Functional enrichment analysis of DEGs. (A, B) The results of the GO annotation analysis of the DEGs. (C, D) The results of the KEGG pathways enrichment analysis of DEGs.

GO and KEGG enrichment analysis of the DEGs were fulfilled by R language. The result of GO analysis (Fig 4A and 4B) expounded that the DEGs were mainly enriched in the GO terms relevant to the immune cells, such as T cell activation, leukocyte cell−cell adhesion, and mononuclear cell differentiation. KEGG enrichment analysis (Fig 4C and 4D) displayed that DEGs were mainly enriched in Cytokine-cytokine receptor interaction, Hematopoietic cell lineage, and Chemokine signaling pathway. The function enrichment analysis of DEGs demonstrated that these genes were prevailingly mapped to the immune-related pathway. Therefore, immune factors played a considerable role in the TME of CM patients.

### PPI network establishment and hub gene filtration

**Fig 5:**
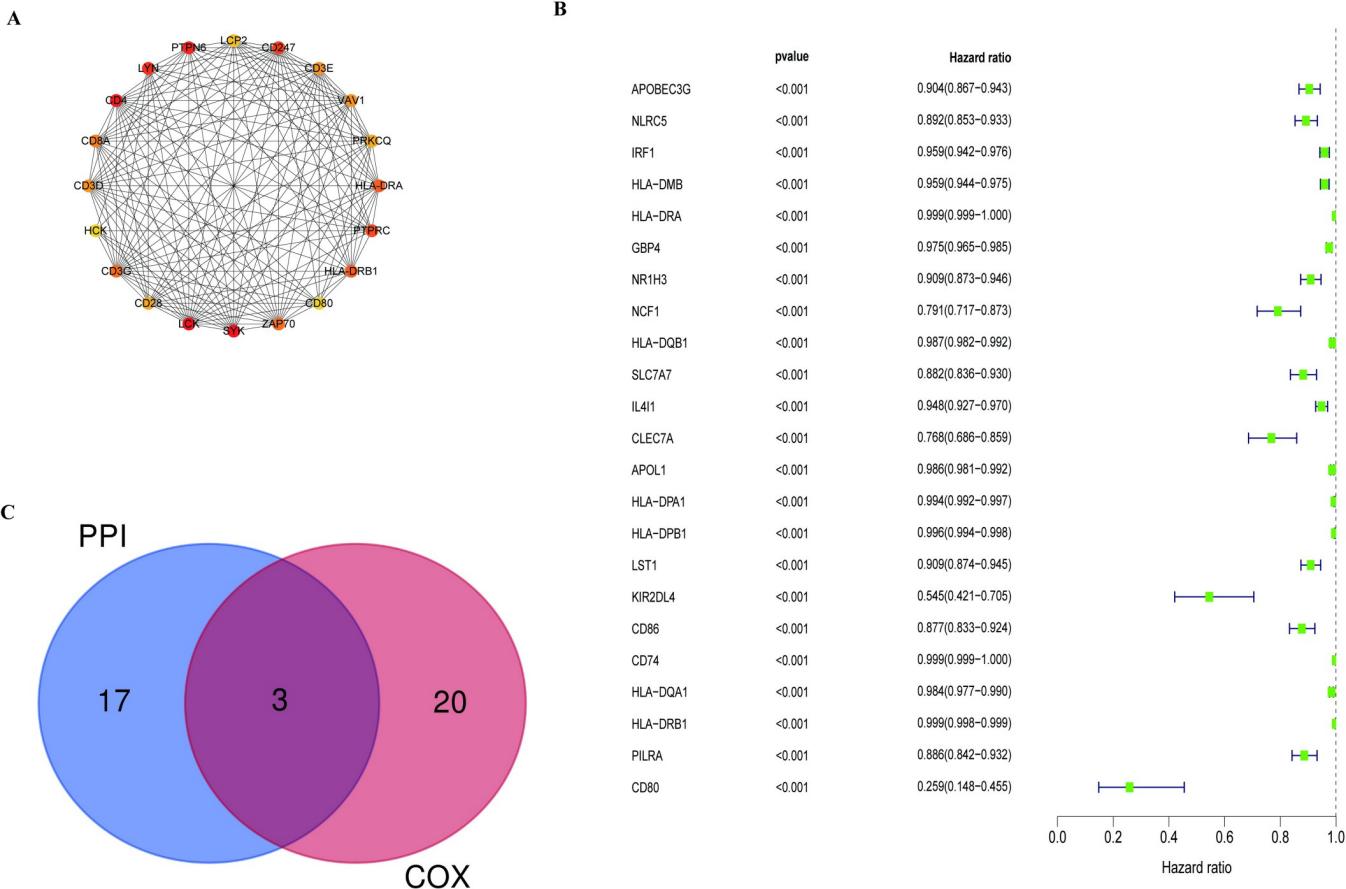
Protein–protein interaction network analysis and univariate Cox regression analysis. (A) The network of top 20 hub genes with high connectivity degree in the PPI network. (B) The Forest map represented the result of the univariate Cox regression analysis. (C) Venn plot showed the intersection part of the result of the PPI network and univariate Cox regression analysis.

PPI network was established by the STRING database and was visualized by Cytoscape to predict protein interactions between each DEG. The genes with the top 20 connectivity degrees in the PPI network were exhibited in Fig 5A and Table 1. Interestingly, all the hub genes were up-regulated genes in CM.

**Table 1:** Top 20 hub genes with higher connectivity degree in the PPI network.

Gene sample	Gene description	Degree
LCK	LCK proto-oncogene, Src family tyrosine kinase	52
SYK	Spleen associated tyrosine kinase	50
CD4	CD4 molecule	47
PTPN6	Protein tyrosine phosphatase non-receptor type 6	46
LYN	LYN proto-oncogene, Src family tyrosine kinase	42
CD247	CD247 molecule	38
PTPRC	Protein tyrosine phosphatase receptor type C	36
HLA-DRA	Major histocompatibility complex, class II, DR alpha	34
HLA-DRB1	Major histocompatibility complex, class II, DR beta 1	34
CD3G	CD3g molecule	33
ZAP70	Zeta chain of T cell receptor associated protein kinase 70	33
CD8A	CD8a molecule	32
CD3E	CD3e molecule	31
CD3D	CD3d molecule	31
VAV1	Vav guanine nucleotide exchange factor 1	31
CD28	CD28 molecule	29
PRKCQ	Protein kinase C theta	29
LCP2	Lymphocyte cytosolic protein 2	28
CD80	CD80 molecule	27
HCK	HCK proto-oncogene, Src family tyrosine kinase	27

### Univariate Cox regression and intersection analysis

Univariate Cox regression analysis was conducted to find the genes associated with the prognosis of the CM patients. As is illustrated in Fig 5B, there were 23 low-risk genes correlated with the prognosis of CM, which played a protective role in the CM patients. The higher the expression levels of these genes, the greater the prognosis of CM patients. Subsequently, intersection analysis was implemented using the hub genes of the PPI network and the result of univariate Cox regression analysis. Only HLA-DRA, HLA-DRB1, and CD80 were in the overlapping part of the Venn plot (Fig 5C) and HLA-DRB1 was chosen for the subsequent analysis.

### Survival analysis of HLA-DRB1 expression

**Fig 6:**
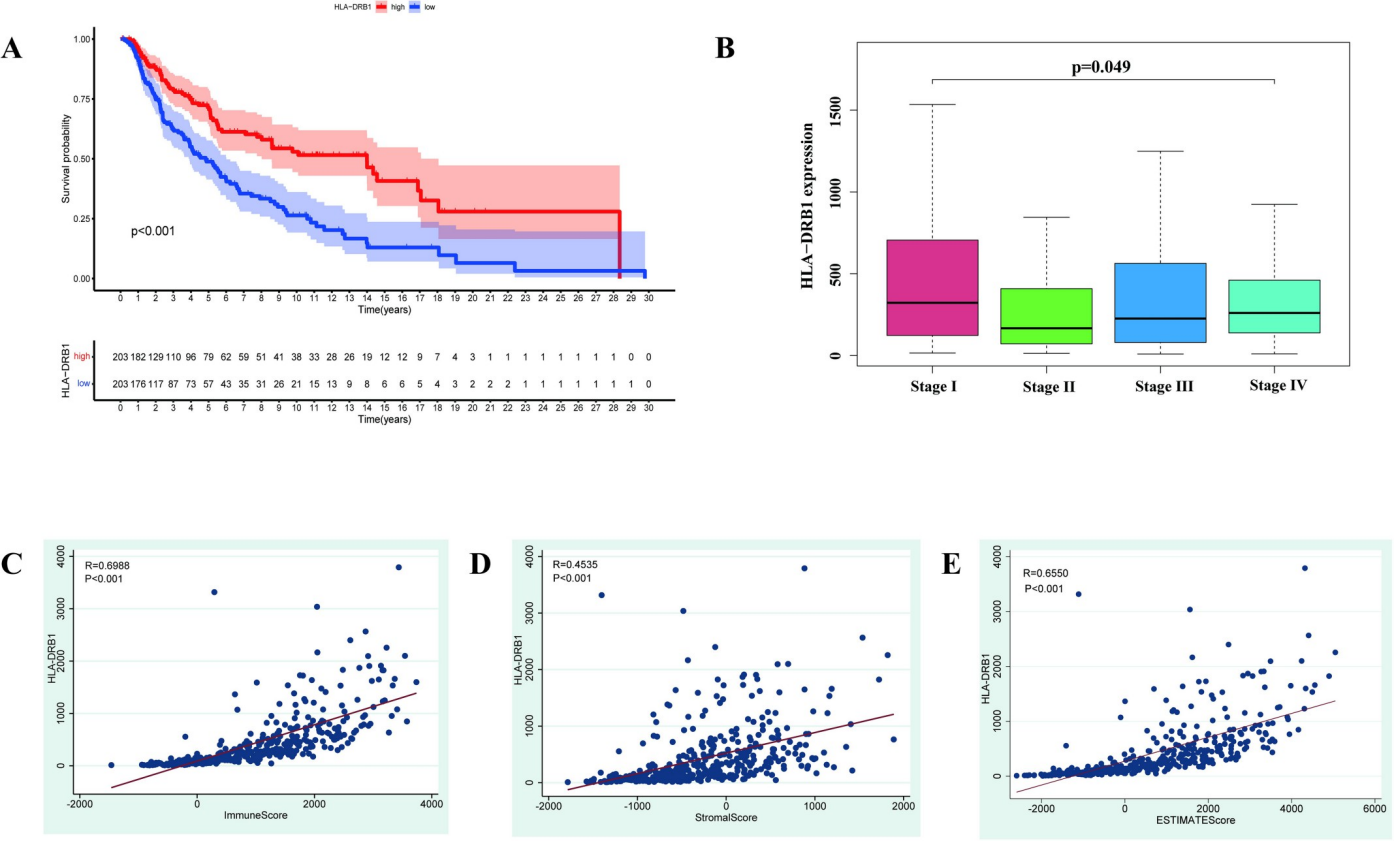
Survival analysis and correlation analysis of HLA-DRB1 expression. (A) The overall survival curve of the high HLA-DRB1 expression group and low HLA-DRB1 expression group. (B) The result of the correlation analysis between the expression level of HLA-DRB1 and the Stage of patients. (C-E) The results of the correlation analysis among the ImmnueScore, StromalScore, ESTIMATEScore, and the expression level of HLA-DRB1.

The 406 CM samples were divided into high expression group and low expression group on the basis of the median value of the expression level of HLA-DRB1. Survival analysis was implemented to explore the difference in OS, PFS, and DFS between the high HLA-DRB1 expression group and low HLA-DRB1 expression group. It can be concluded from Fig 6A, S1A and S1B Fig that the OS, PFS, and DFS of the patients in the high HLA-DRB1 expression group were higher than that in the patients in the low HLA-DRB1 expression. Obviously, it can be inferred that the expression level of HLA-DRB1 was positively relevant to the survival and prognosis of CM patients.

### Correlation analysis among the Stage, TME and HLA-DRB1 expression

The correlation analysis was enforced to research the relevance among the TME, Stage, and the expression level of HLA-DRB1. As is revealed in Fig 6B, the expression level of HLA-DRB1 among the patients in different Stages was deemed to have significantly different, and the patients in Stage I owned a higher expression level of HLA-DRB1 than the patients in other Stages. It can be inferred that the expression level of HLA-DRB1 decreased with the progression of the disease. Hence, the expression level of HLA-DRB1 was not only positively correlated with the survival and prognosis of CM patients but also concerned with the invasion and metastasis of CM patients. The correlation scatter plots among the ImmuneScore, StromalScore, and the expression level of HLA-DRB1 were displayed in Fig 6C and 6D. Uniformly, ImmuneScore (Fig 6C, R = 0.6988, p-value < 0.001), StromalScore (Fig 6D, R = 0.4535, p-value < 0.001) and ESTIMATEScore (Fig 6E, R = 0.6550, p-value < 0.001) were positively correlated with the expression level of HLA-DRB1. Therefore, the expression level of HLA-DRB1 was positively related to the ImmuneScore, StromalScore, and ESTIMATEScore.

### ROC curves and independence of HLA-DRB1 expression

**Fig 7:**
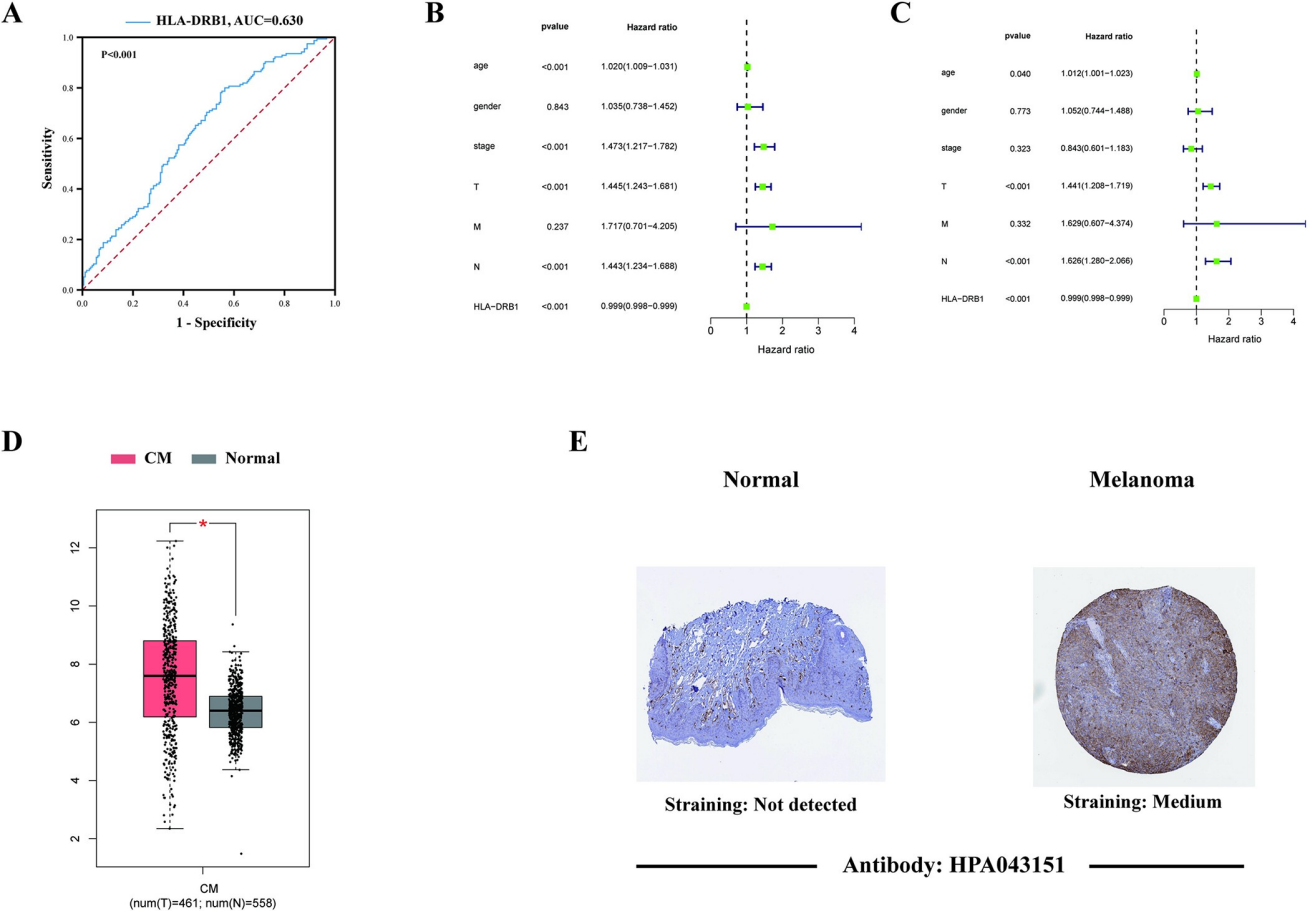
ROC curves, independent prognostic analysis and the validation of the expression level of HLA-DRB1. (A) The ROC curve of the expression level of HLA-DRB1. (B, C) The results of univariate and multivariate Cox regression analysis. (D) The box plot displayed the expression level of HLA-DRB1 in the melanoma and normal skin samples from the TCGA and GTEx databases based on the GEPIA web server. (E) The representative immunohistochemical images of HLA-DRB1 in the normal skin tissue and melanoma tissue from the Human Protein Atlas (HPA) database.

Fig 7A exhibited the result of ROC curves of HLA-DRB1 expression (AUC = 0.630, p-value <0.001). This result indicated that the expression level of HLA-DRB1 was highly sensitive and accurate in predicting the survival of CM patients. As is demonstrated in Fig 7B and 7C, the p-value of the expression level of HLA-DRB1 was less than 0.001 both in the univariate and multivariate Cox regression analysis. Accordingly, it can be concluded that the expression level of HLA-DRB1 can work as an independent prognostic factor in CM and it is more accurate than the Stage of patients in predicting survival and prognosis of CM patients.

### Validation of the expression level of HLA-DRB1

The GEPIA webserver was applied to validate the expression level of HLA-DRB1 in the melanoma (461 tumor samples from the TCGA database) and the matching normal (558 normal skin samples from the GTEx database) samples. It can be founded in Fig 7D that the expression level of HLA-DRB1 in the melanoma samples was higher than in the normal samples. And the result of the representative immunohistochemical images (Fig 7E) of HLA-DRB1 in normal skin tissue and melanoma tissue from The Human Protein Atlas (HPA) database were in accordance with the gene expression box plots. As is shown in Fig 7E, the staining intensity of the melanoma tissues was deeper than that in normal tissue. The deeper the staining intensity, the higher the expression of HLA-DRB1. These results further supported our findings, the high expression of HLA-DRB1 in the melanoma tissues played a protective role and was positively relevant to the survival and prognosis of patients.

### Correlation between HLA-DRB1 expression and the ratio of TICs

**Fig 8:**
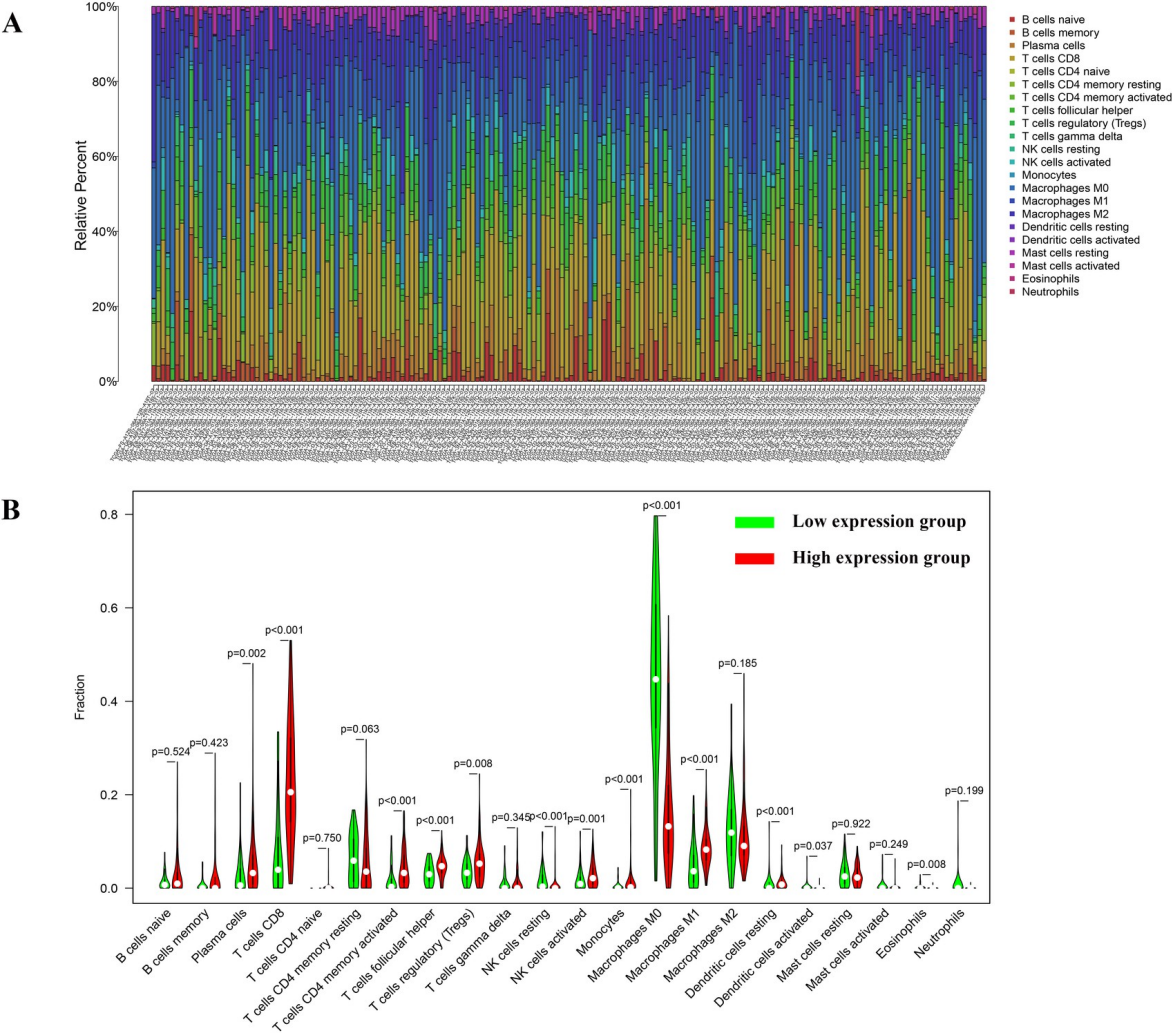
TIC abundance profiles in the CM samples and differentiation analysis between the expression level of HLA-DRB1 and the content of immune cells. (A) Barplot displayed the ratio of 22 kinds of TICs in different CM samples. The abscissa of the barplot is the sample ID and the ordinate is the abundance of each TIC. (B) The violin plot depicted the differences in the ratio of 22 types of TICs between the high HLA-DRB1 expression group and low HLA-DRB1 expression group.

**Fig 9:**
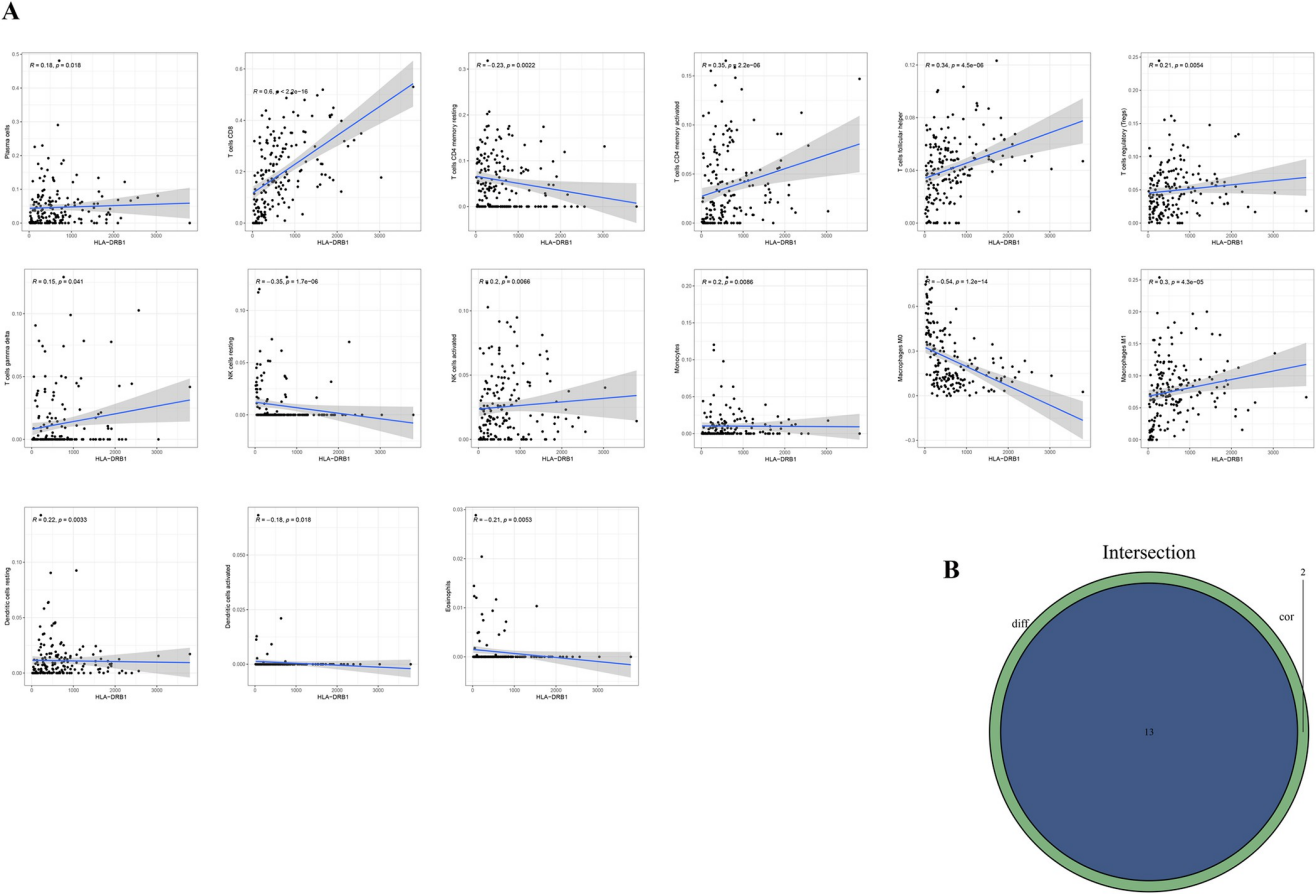
Correlation between TICs proportion and HLA-DRb1 expression. (A) The scatter plot described 15 types of TICs whose proportions were correlated with the expression of HLA-DRB1. (B) The Venn plot displayed the result of the intersection analysis.

CIBERSORT computational method was applied to estimate the TICs abundance profile of 406 CM samples to further confirm the correlation between the expression level of HLA-DRB1 and the TME. Whereafter, 22 types of immune cell abundance profiles in different CM samples were established (Fig 8A). Subsequently, differentiation analysis and correlation analysis were executed to explore the relationship between the HLA-DRB1 expression and immune cell infiltration in the TME. As is shown in Fig 8B, there were significant differences in the proportion of 13 kinds of immune cells by comparing the infiltration of immune cells in the high HLA-DRB1 group and low HLA-DRB1 group. Fig 9A demonstrated the result of the correlation analysis between the HLA-DRB1 expression and the proportion of immune cells. It can be seen that there were 15 kinds of immune cells, whose proportion was significantly correlated with the expression level of HLA-DRB1. Eventually, the 13 kinds of immune cells obtained from the differentiation analysis and 15 kinds of immune cells obtained from the correlation analysis were used to conduct intersection analysis. The Venn plot (Fig 9B) displayed the result of intersection analysis, and there were 13 kinds of immune cells in the overlapping part of the Venn plot. Among them, 9 types of TICs were positively related to the expression level of HLA-DRB1. Only 4 types of TICs were negatively relevant to the expression level of HLA-DRB1. From this, it can be inferred that the expression levels of HLA-DRB1 can alter the immune activity of TME by affecting the infiltration of immune cells in the TME.

## Discussion

In this study, we found that the immune component in TME was associated with the survival and prognosis of CM patients by analyzing the transcriptome expression profiles and clinical data of 406 CM samples from the TCGA database. In addition, the correlation analysis among the ImmuneScore, StromalScore, ESTIMATEScore and the Stage of the CM patients revealed that the ImmuneScore and ESTIMATEScore were significantly correlated with the Stage of CM patients. And the Stage of melanoma is composed of micro staging of the primary melanoma, clinical and radiographic assessment of regional and distant metastatic [36]. Consequently, it can be inferred that the ratio of immune components and stromal component in the TME may play an essential role in the invasion and metastasis of CM patients, which is consistent with previous [22]. These results not only demonstrate the need to explore the interaction between melanoma cells and immune cells, but also provide new ideas for finding more effective treatments.

Over the past few years, emerging immune checkpoint inhibitors (ICIs), such as anti-PD-L1 (programmed cell death ligand 1) and anti-CTLA-4 (cytotoxic T-lymphocyte antigen4), have presented significant clinical efficacy and observably improved the treatment of the unresectable and metastatic melanoma as well as those with a high risk of recurrence after resection [2, 37–39]. Despite these significant advances in cancer immunotherapy, primary and acquire drug resistance is a key obstacle to further improving prognosis of CM patients. Studies indicated that approximately 40–65% of CM patients receiving anti-PD-1 therapy are resistant to immune checkpoints and more than 70% of CM patients receiving anti CTLA-4 therapy are resistance to immune checkpoints [21]. Accordingly, there is a huge demand for novel targets and candidates for the immunotherapy of CM patients. In the present study, intersection analysis was conducted using the hub genes from PPI network and the result of univariate Cox regression analysis and identified a predictive biomarker, HLA-DRB1. The results of survival analysis and correlation analysis of HLA-DRB1 expression proved that the HLA-DRB1 expression in CM patients was observably correlated with the OS, PFS, DFS and Stages. Patients in high HLA-DRB1 expression group had higher survival rate and better prognosis than those in low HLA-DRB1 expression group. Furthermore, a previous study based on two independent anti-PD-1-treated melanoma patient cohorts showed that MHC II positivity in melanoma cells was associated with treatment response, PFS, and OS. And anti- PD-1/PD-L1 therapy has superior clinical efficacy in CM patients with MHC II (such as HLA-DR) expression [40]. Moreover, Douglas B. Johnson et al. also conducted a further study based on pre-treatment tumor biopsy tissue from 166 patients receiving anti-PD-1 therapy across 10 academic cancer centers and found a significant improvement in progression-free survival, and overall survival in melanoma patients with high HLA-DR expression. And among melanoma patients with high HLA-DR levels, 80% of them responded to PD-1 blockers [41]. In addition, the findings of Scott J. Rodig et al. are consistent with those of Douglas B. Johnson et al. They found that melanoma MHC class II expression was associated with a better prognosis in patients initially treated with anti-PD-1 [42]. Therefore, the HLA-DRB1 perhaps is not only a new latent prognostic marker, therapeutic target in the TME of the CM patients, but also a potential biomarker of high likelihood of high response to anti-PD-1/PD-L1 drugs.

HLA-DRB1 belongs to the HLA class II β chain paralogs. The class II molecules are heterodimers composed of an α chain (DRA) and a β chain (DRB) expressed by antigen-presenting cells (including macrophages, B cells and dendritic cells) [26]. The class II molecules are not only necessary for presenting peptides to T-helper CD4^+^ cells and inducing their activation, but are also critical for the development of the T cell repertoire and for the proliferation and differentiation of antigen-specific CD4 T cells in adaptive immune responses [43–46]. Their gene products can also participate in the inflammatory responses, antigen processing and presentation as part of the adaptive immune response, and interact with NK cells and cytokines as part of the innate immune response [47]. Therefore, HLA-DRB1 may promote its antitumor activity by interacting with NK cells and cytokines or inducing T cell activation, thereby improving the survival and the prognosis of CM patients.

It can be concluded from the univariate Cox regression analysis that HLA-DRB1 was a protective factor for CM patients, its expression level was positively relevant to the survival of the CM patients. Moreover, the correlation analysis between the Stage of patients and the HLA-DRB1 expression showed that the expression level of HLA-DRB1 decreased with the progression of melanoma. And the validation of the expression level of HLA-DRB1 based on the GEPIA web server and the HPA database showed that the expression level of HLA-DRB1 in the melanoma tissue was higher than that in normal skin tissue. The study of Yan Degenhardt et al. consistent with our study. Their study demonstrated that the expression level of MHC II genes was increased compared to normal melanocytes in the vertical growth phase (VGP) of melanoma, but decreased in metastatic melanoma [48]. Additionally, earlier studies have revealed that the variations in the expression levels of MHC class II molecules may promote tumor cells to evade immune surveillance and facilitate tumor metastasis [49, 50]. And the study of Aptsiauri N et al. revealed that the deficiency of the HLA gene expression due to viral infection, somatic mutation or other causes may affect immune suppression and cancer development [51].

Although HLA-DRB1 has many polymorphic variants, whether the differences in the HLA-DRB1 allele expression are correlated the survival and disease progression in CM patients remains to be further research [52]. A previous study reported that increased HLA-DRB1*1101 expression is associated with increased risk of recurrence in patients with localized melanoma [53]. Unfortunately, their study was limited to patients with localized melanoma at Anderson Cancer Center, and their findings may not apply to patients with other Stage of melanoma. Furthermore, the study of Anjali Dhall et al. revealed that the patients with HLA-DRB1*1101 has a higher mean overall survival time than patients without HLA-DRB1* 1101 [54]. Accumulated studies have demonstrated that the expression levels of MHC I and MHC II genes in melanoma cells are correlated with anti-tumor immune response, and the expression levels of HLA II genes, especially HLA-DP and HLA-DR, are positively correlated with time to progression (TTP) and overall survival of CM patients [52, 55, 56]. Therefore, it can be inferred that the down-regulation of HLA-DRB1 in CM patients may be a mechanism of immune escape of melanoma cells. And the down-regulation expression of HLA-DRB1 in melanoma tissues is not only associated with metastasis and poor prognosis in CM patients, but also may accelerate the transition of TME from tumor suppressive to tumor friendly.

In this study, 9 types of TICs (such as T cells CD8, T cells CD4 memory activated, NK cells activated etc.) were found to be positively correlated with the expression level of HLA-DRB1. Similarly, the study of MR Bernsen et al. manifested that the expression levels of MHC II molecules in melanoma cells positively correlated with the presence of tumor-infiltrating lymphocytes, regression of lesions and overall survival of patients [55]. Tumor-infiltrating lymphocytes (TILs) are a heterogenous group composed of effector T cells, tolerogenic or T regulatory (Treg) cells, functionally exhausted T cells, natural killer (NK) cells, macrophages, dendritic cells (DCs) and other types of immune cells [57, 58]. The function of TILs in malignant melanoma and its association with the prognosis of CM patients have been extensively researched [59]. Previous studies have shown that dense lymphocyte infiltration in primary melanoma can improve the survival and prognosis of CM patients [57]. And the 5-year survival rate of the CM patients with active TIL response was 77% while the 5-year survival rate of the patients without active TIL response was only 55% [57, 60]. Moreover, Hersey, Rosenberg and Tobias Schatton et al. also found that TILs may play an important role in promoting tumor clearance [59, 61, 62]. Numerous studies have demonstrated that CD8^+^ T cells, CD4^+^ T cells and NK cells can play their anti-tumor role by cytotoxic molecules or mediating immunological surveillance and clearance of virus infected cells and tumor-transformed cells or inducing antigen-independent immune response against malignant cells [63–67]. Hence, the amount of T cells CD8, T cells CD4 memory activated, NK cells activated were positively relevant to the expression level of HLA-DRB1, suggesting that HLA-DRB1 expression may be closely related to the maintenance of anti-tumor immune activity of the TME.

Interestingly, it can be learned from the correlation analysis between HLA-DRB1 expression and the ratio of TICs that the ratio of TICs in patients with high HLA-DRB1 expression is significantly higher than that in patients with low HLA-DRB1 expression. However, the number of Tregs in the TME has been reported to be inversely correlated with survival in both mouse and human melanoma [68]. Treg cells can inhibit CD8^+^ T cells through intercellular interactions and can also induce CD8^+^ T cell dysfunction by secreting cytokines that promote tumor metastasis [69, 70]. Therefore, some scholars have proposed that Treg cells play a key role in local tumors, while some reports have suggested that the ratio of Treg/CD8^+^ T cells is more important [20, 71, 72]. Therefore, we further studied the correlation between HLA-DRB1 expression and the ratio of Tregs/CD8^+^ T cells, and the result showed that HLA-DRB1 expression was negatively correlated with the ratio of Tregs/CD8^+^ T cells (S2 Fig, R = -0.1781, p-value = 0.0006). Hence, although the HLA-DRB1 expression was negatively correlated with the content of Tregs in the TME of CM patients, the overall effect of those two lymphocytes was positive for the prognosis of CM patients. However, further research is still required.

In this study, we found that HLA-DRB1 may be a new potential prognostic factor and therapeutic target of cutaneous melanoma and an indicator of tumor microenvironment remodeling according to a series of bioinformatics analyses. However, there also are some limitations in our study. Firstly, the CM sample size in TCGA database is limited, and the clinical information of some samples is incomplete, which makes less data available for analysis. Secondly, there are difference in the number of patients in different stages, which may have some impact on the results of the study. In addition, the conclusions of our study are mainly based on data from public databases, and further prospective studies are highly need. However, we still believe that the expression level of HLA-DRB1 has the ability to accurately predict the prognosis of melanoma patients, which provides a new idea for the treatment of CM.

## Conclusion

Our bioinformatics analysis identified 908 up-regulated genes and 6 down-regulated genes jointly owned by the ImmuneScore and StromalScore based on the transcriptome profiling data obtained from the TCGA database. Afterwards the tumor microenvironment-related genes in CM were identified by the ESTIMATE algorithm. HLA-DRB1was a latent prognostic factor and therapeutic target for the CM patients. The high expression of HLA-DRB1 was positively correlated to the prognosis and survival of CM patients. Furthermore, the downregulation of HLA-DRB1 is not only associated with metastasis and poor prognosis of CM patients, but also might accelerate the change of TME from tumor suppressive to tumor friendly. Further researches were needed to confirm the results of our study.

## Supporting information

S1 Fig Survival analysis of HLA-DRB1 expression. (A) progression-free survival curve, (B) disease-free survival curve of high HLA-DRB1 expression group and low HLA-DRB1 expression group.
(TIF)

S2 Fig The result of correlation analysis between the HLA-DRB1 expression and the ratio of Tregs/CD8+ T cells. The abscissa is the ratio of Tregs/CD8+ T cells and the ordinate is the expression level of HLA-DRB1. Pearson correlation coefficient (R) = -0.1781, p-value = 0.0006.
(TIF)

S1 File (ZIP)

S2 File (ZIP)

S3 File (ZIP)

S4 File (ZIP)

S5 File (ZIP)

S6 File (ZIP)

S7 File (ZIP)

S8 File (ZIP)

S9 File (ZIP)

S10 File (ZIP)

S11 File (ZIP)

S12 File (ZIP)

S13 File (ZIP)

S14 File (ZIP)

S15 File (ZIP)

S16 File (ZIP)
