# Supplementary figures and images for: HLA-DRB1: A new potential prognostic factor and therapeutic target of cutaneous melanoma and an indicator of tumor microenvironment remodeling
Source: PLoS One. 2022 Sep 21;17(9):e0274897. doi: 10.1371/journal.pone.0274897 (PMC9491554; doi:10.1371/journal.pone.0274897)

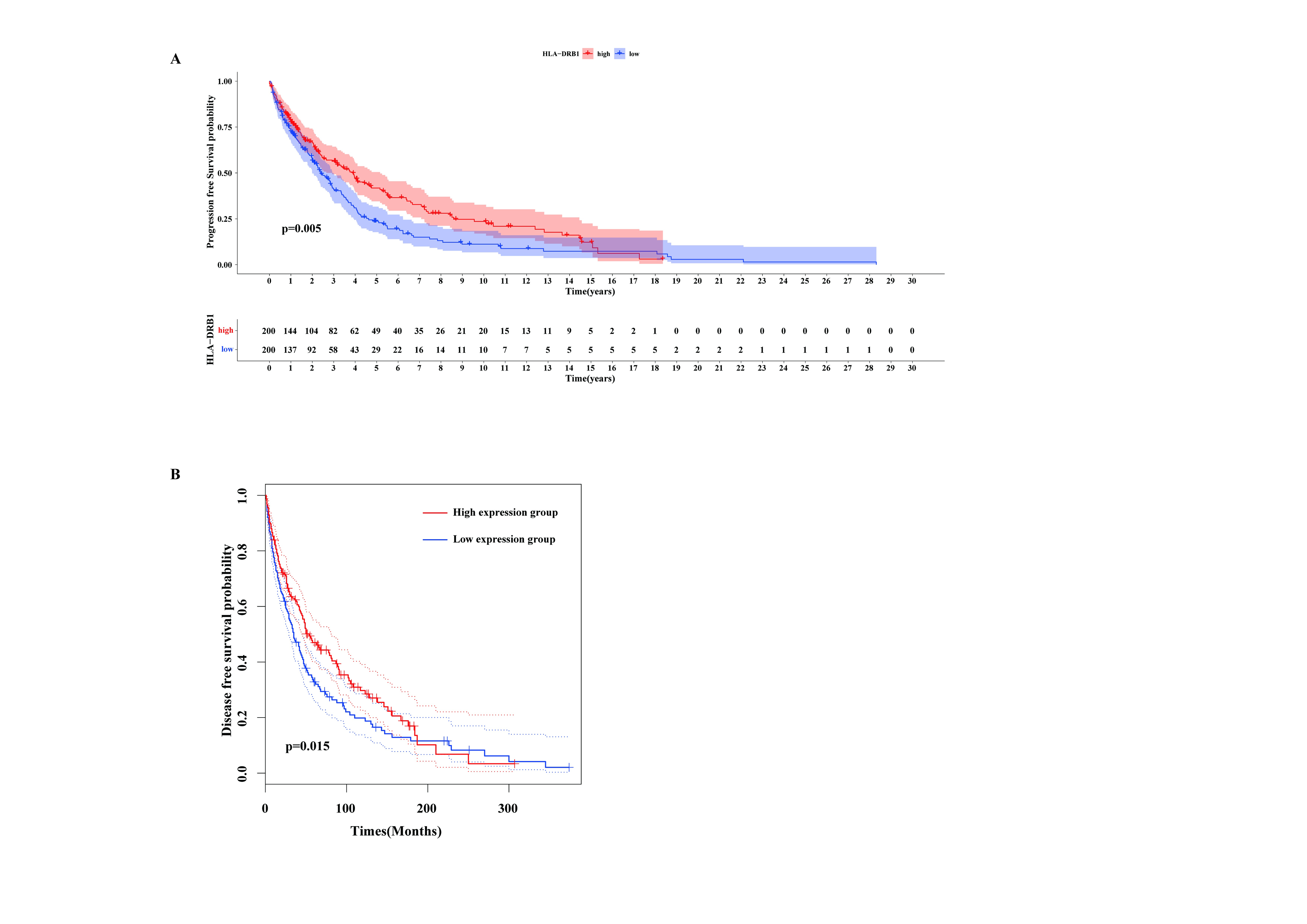

Supplement: S1 Fig — (A) progression-free survival curve, (B) disease-free survival curve of high HLA-DRB1 expression group and low HLA-DRB1 expression group. (TIF) [file pone.0274897.s001.tif]

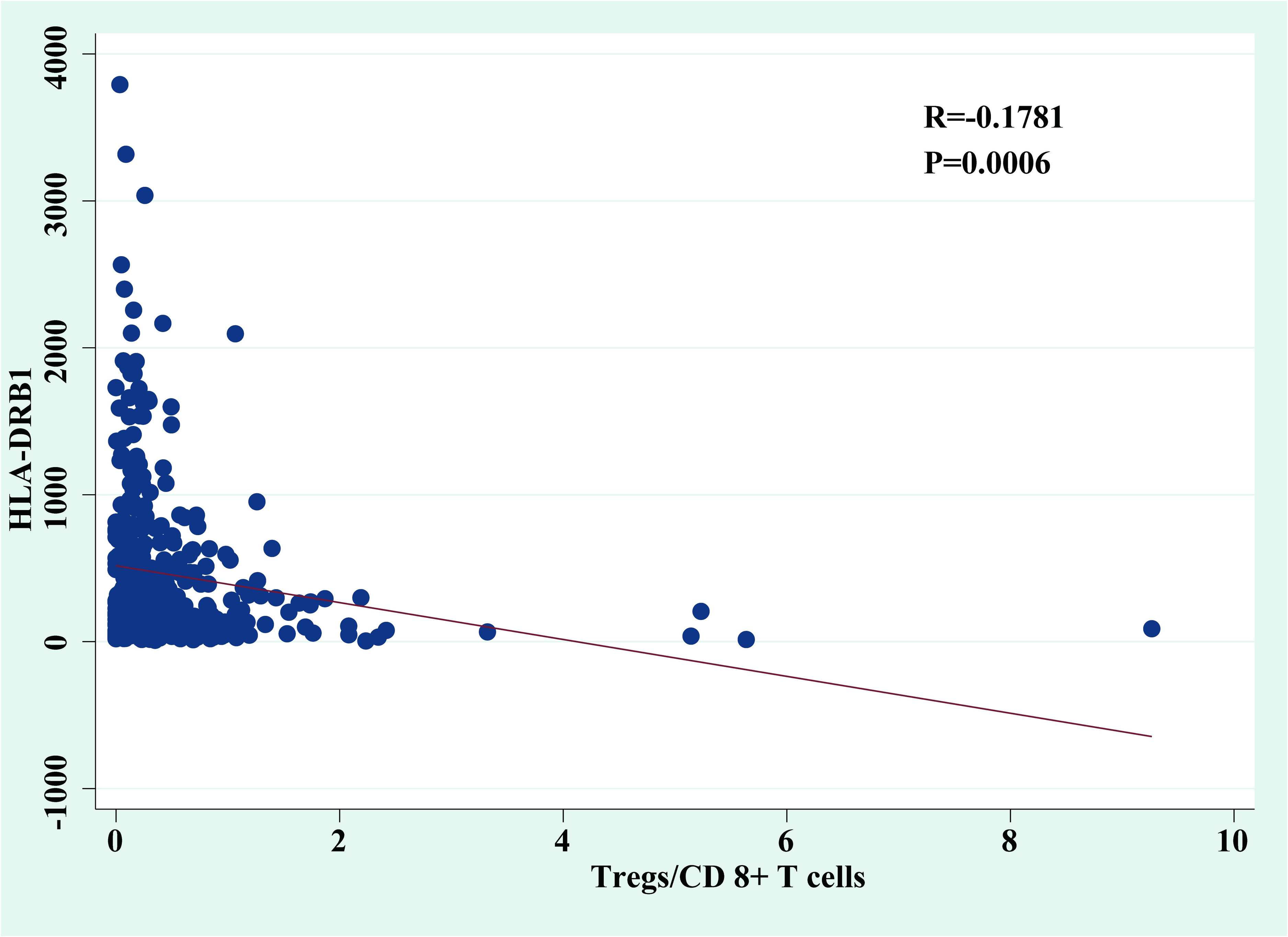

Supplement: S2 Fig — The abscissa is the ratio of Tregs/CD8+ T cells and the ordinate is the expression level of HLA-DRB1. Pearson correlation coefficient (R) = -0.1781, p-value = 0.0006. (TIF) [file pone.0274897.s002.tif]

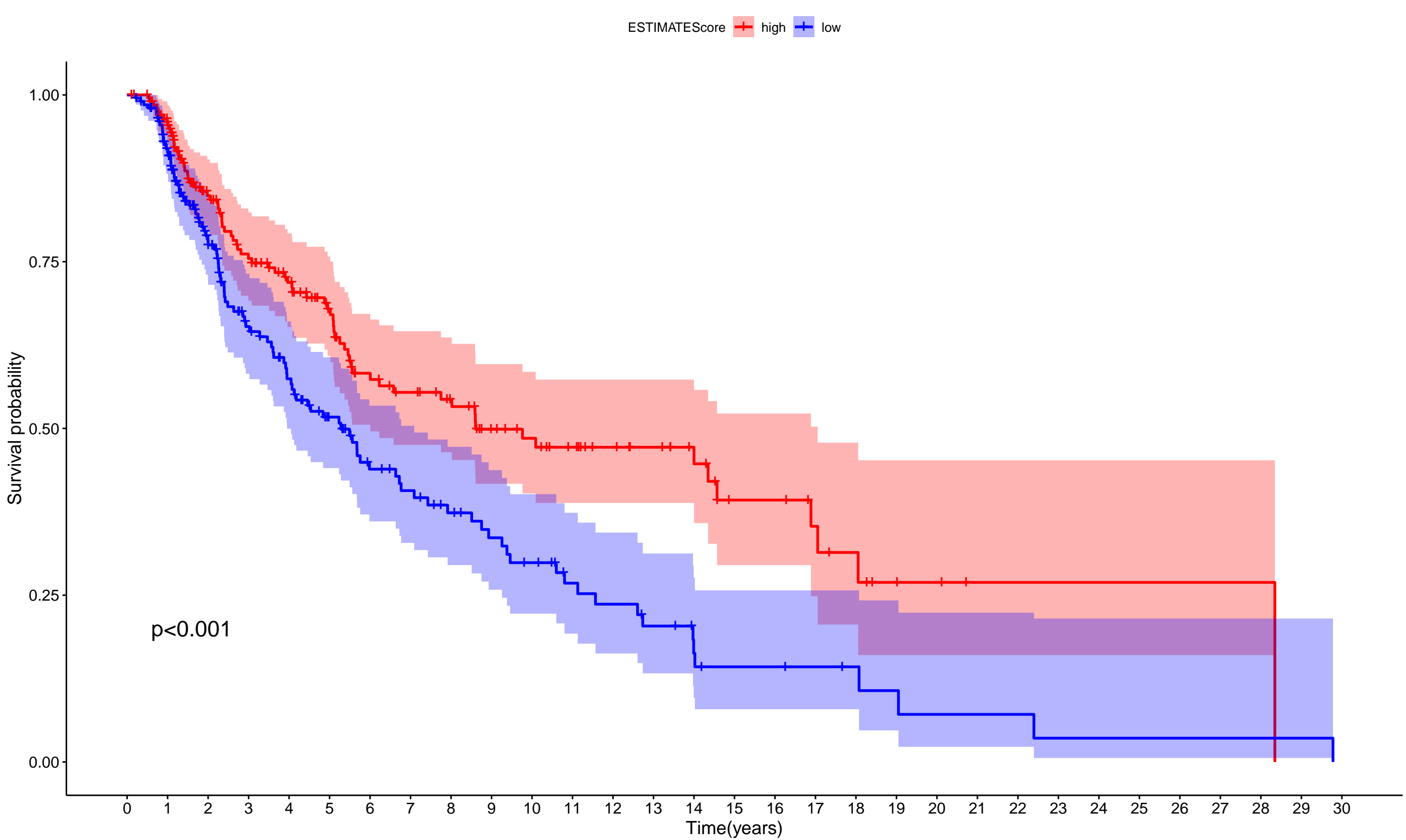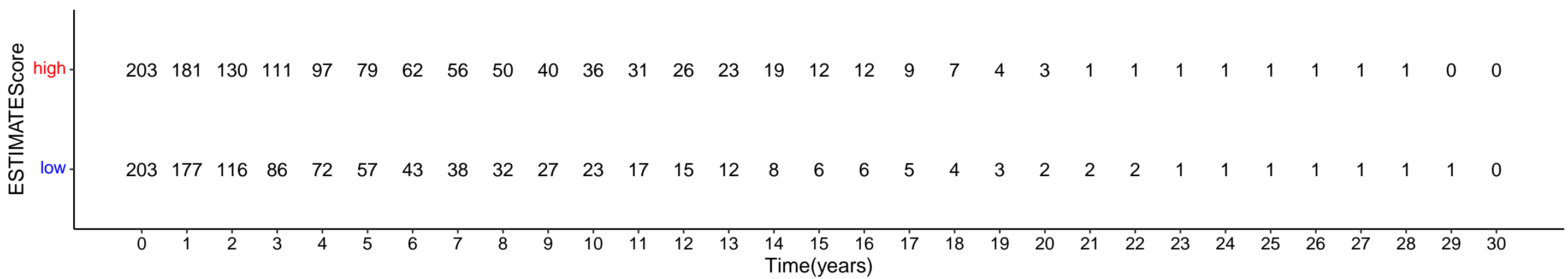

Supplement: S3 File — (ZIP) [file pone.0274897.s005.zip › Step 3.Survival analysis based on ImmuneScore, StromalScore and ESTIMATEScore/output files/sur.ESTIMATEScore.pdf]

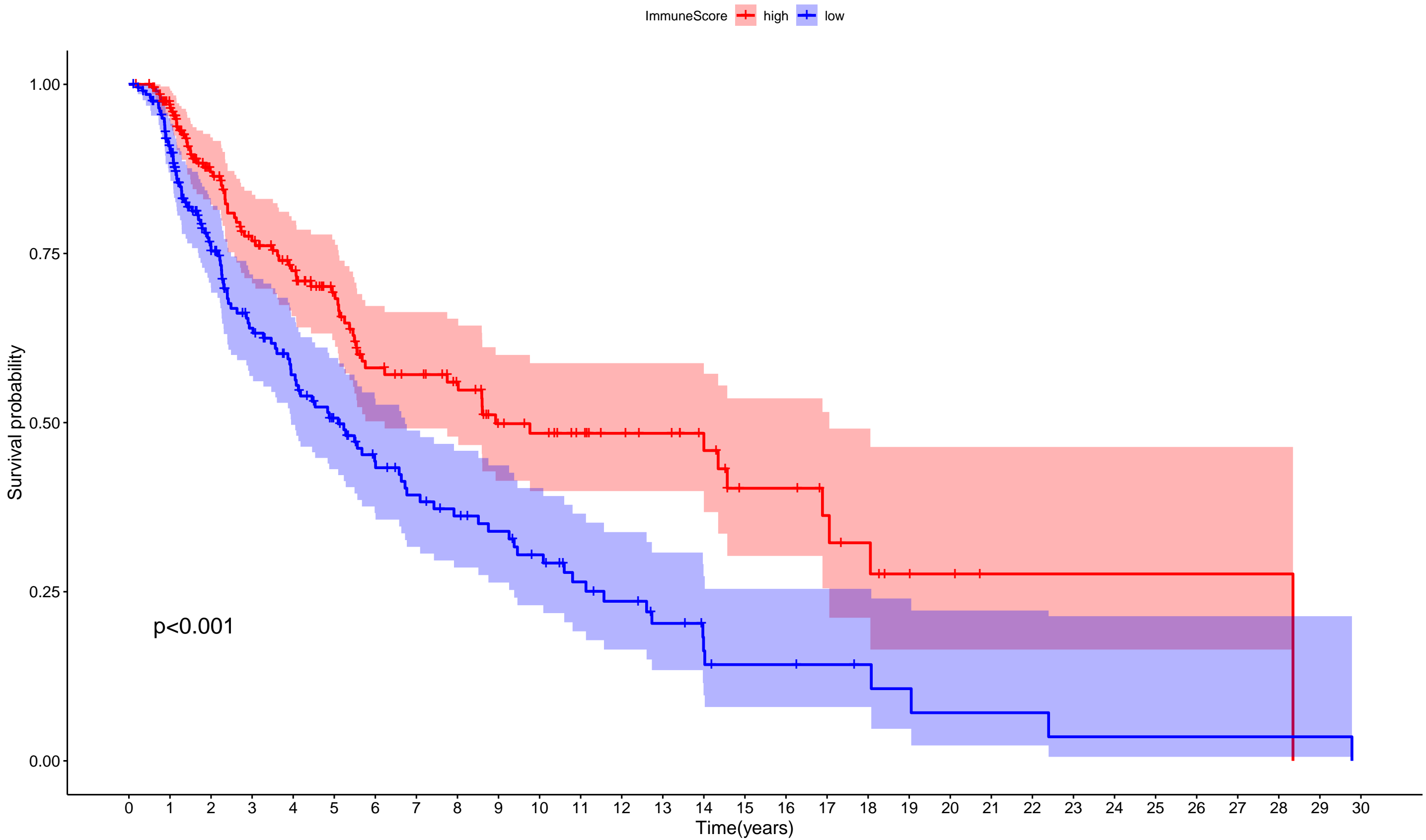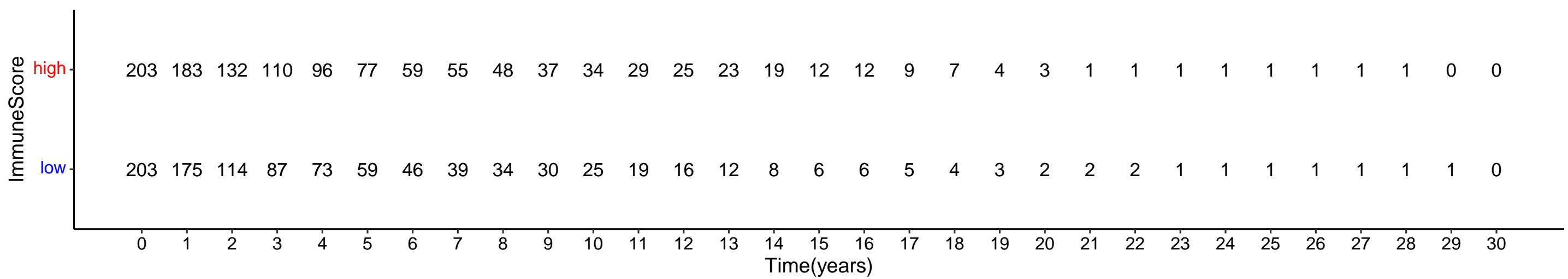

Supplement: S3 File — (ZIP) [file pone.0274897.s005.zip › Step 3.Survival analysis based on ImmuneScore, StromalScore and ESTIMATEScore/output files/sur.ImmuneScore.pdf]

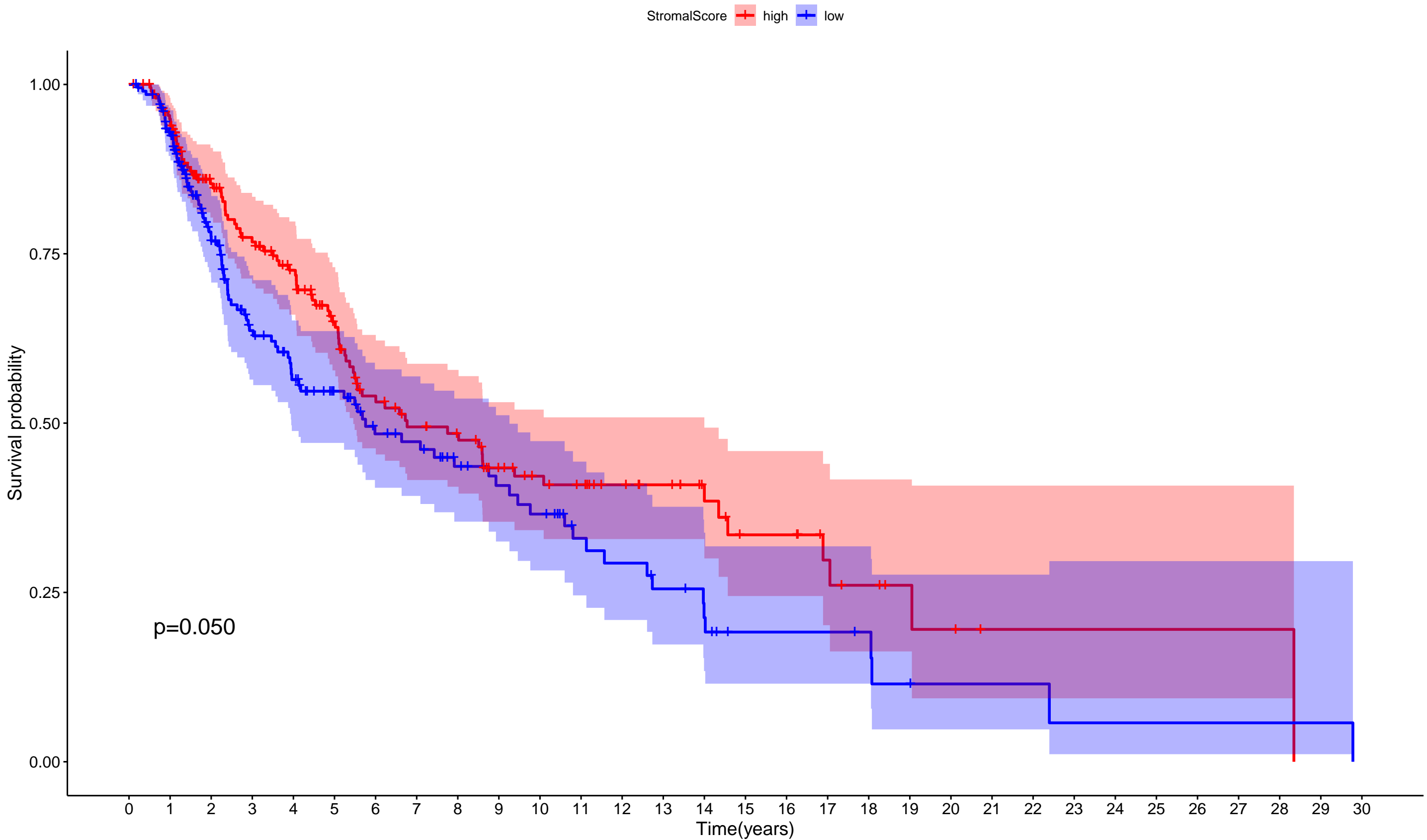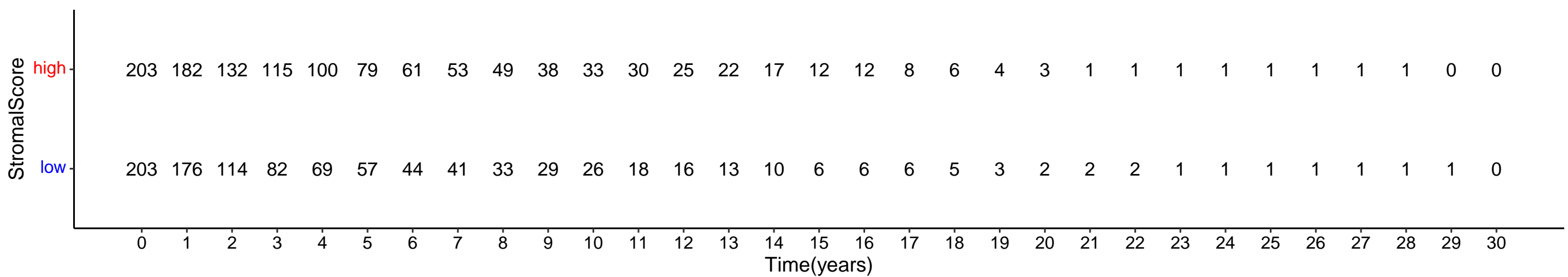

Supplement: S3 File — (ZIP) [file pone.0274897.s005.zip › Step 3.Survival analysis based on ImmuneScore, StromalScore and ESTIMATEScore/output files/sur.StromalScore.pdf]

age 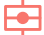 <=65 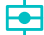 >65

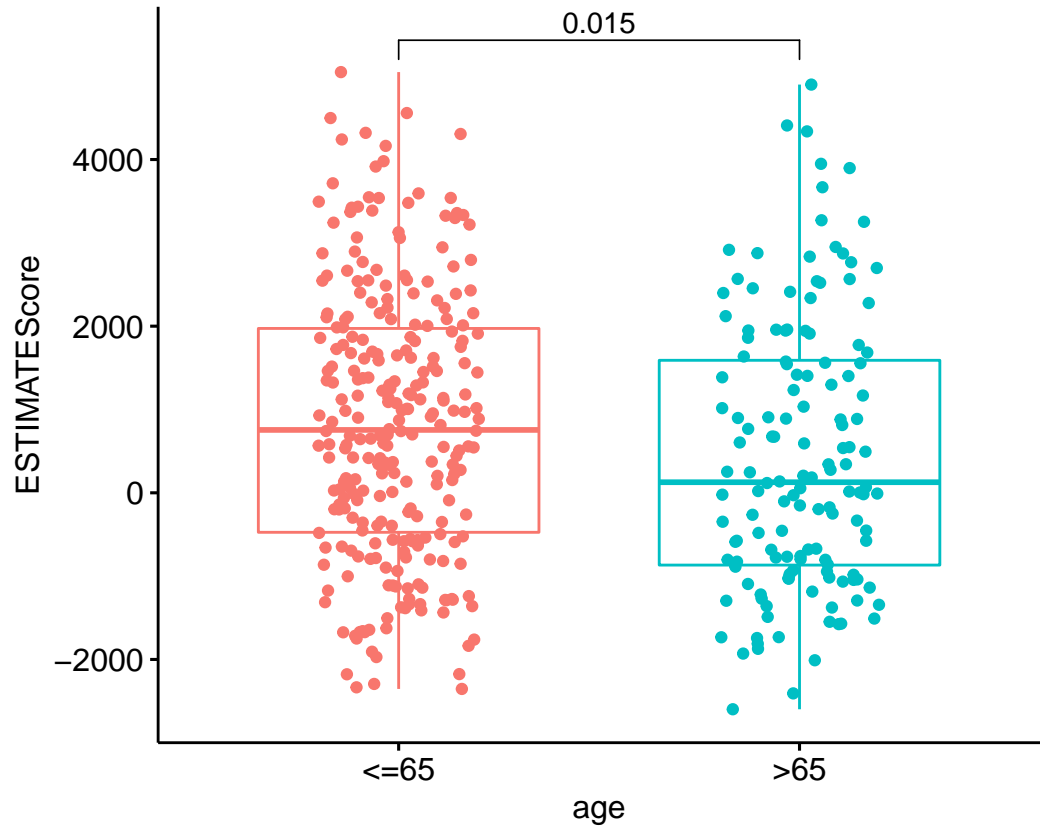

Supplement: S4 File — (ZIP) [file pone.0274897.s006.zip › Step 4.Clinical correlation analysis of the TME/age and Ulceration/output files/ESTIMATEScore.age.pdf]

Ulceration 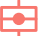 No 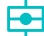 Yes

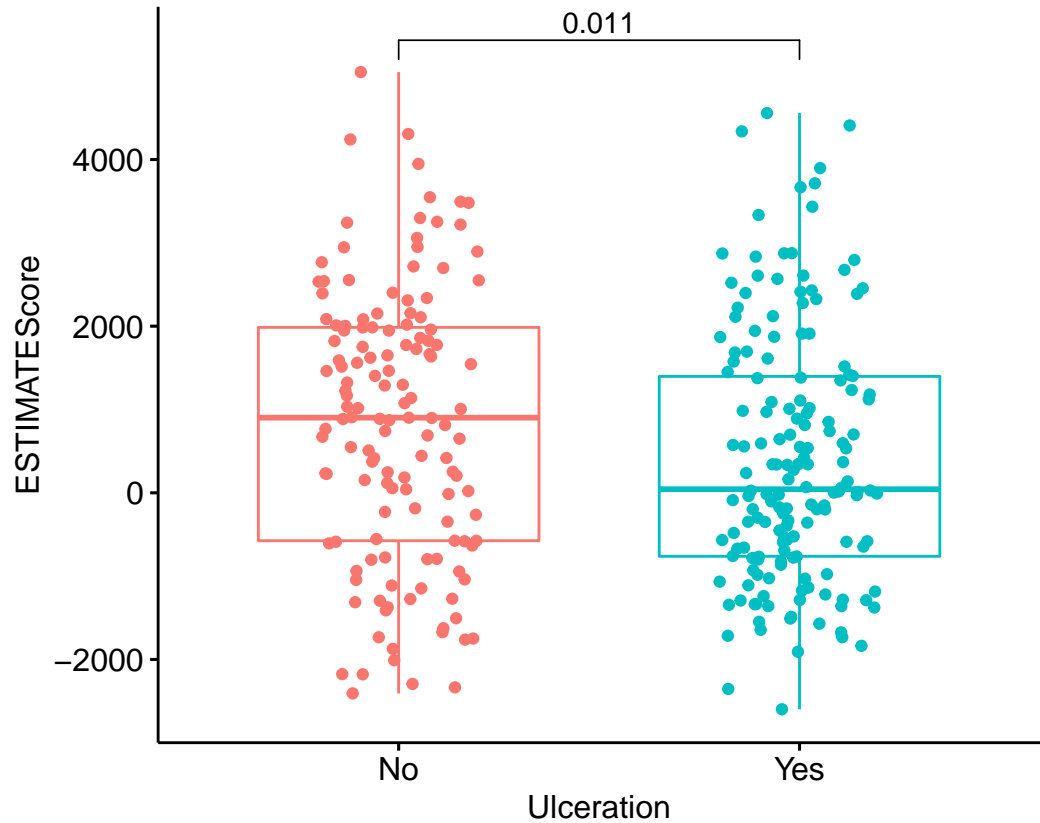

Supplement: S4 File — (ZIP) [file pone.0274897.s006.zip › Step 4.Clinical correlation analysis of the TME/age and Ulceration/output files/ESTIMATEScore.Ulceration.pdf]

age 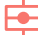 <=65 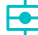 >65

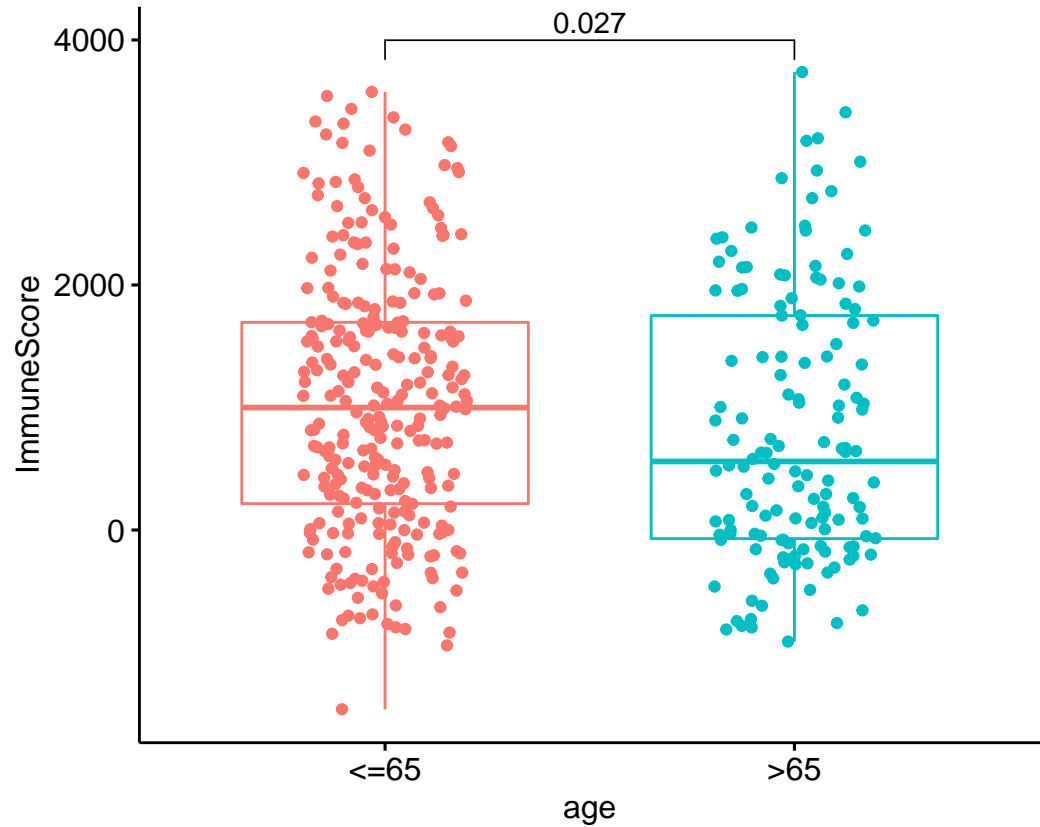

Supplement: S4 File — (ZIP) [file pone.0274897.s006.zip › Step 4.Clinical correlation analysis of the TME/age and Ulceration/output files/ImmuneScore.age.pdf]

Ulceration 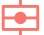 No 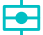 Yes

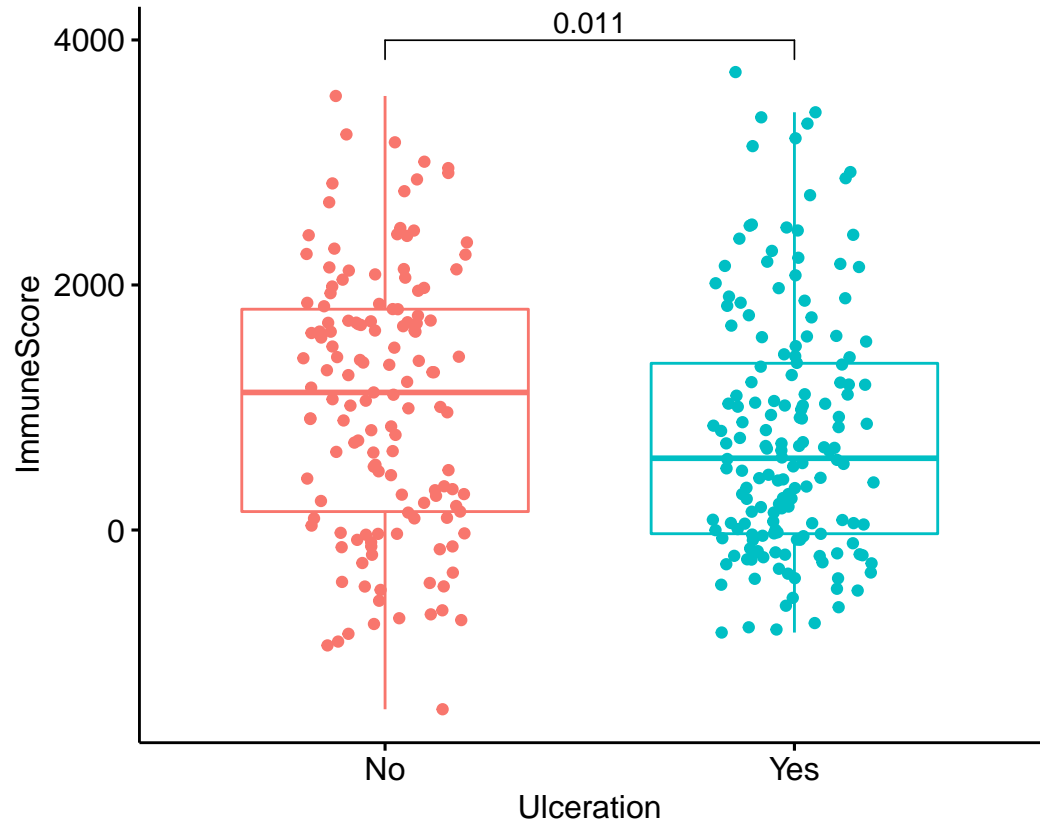

Supplement: S4 File — (ZIP) [file pone.0274897.s006.zip › Step 4.Clinical correlation analysis of the TME/age and Ulceration/output files/ImmuneScore.Ulceration.pdf]

age 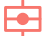 <=65 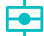 >65

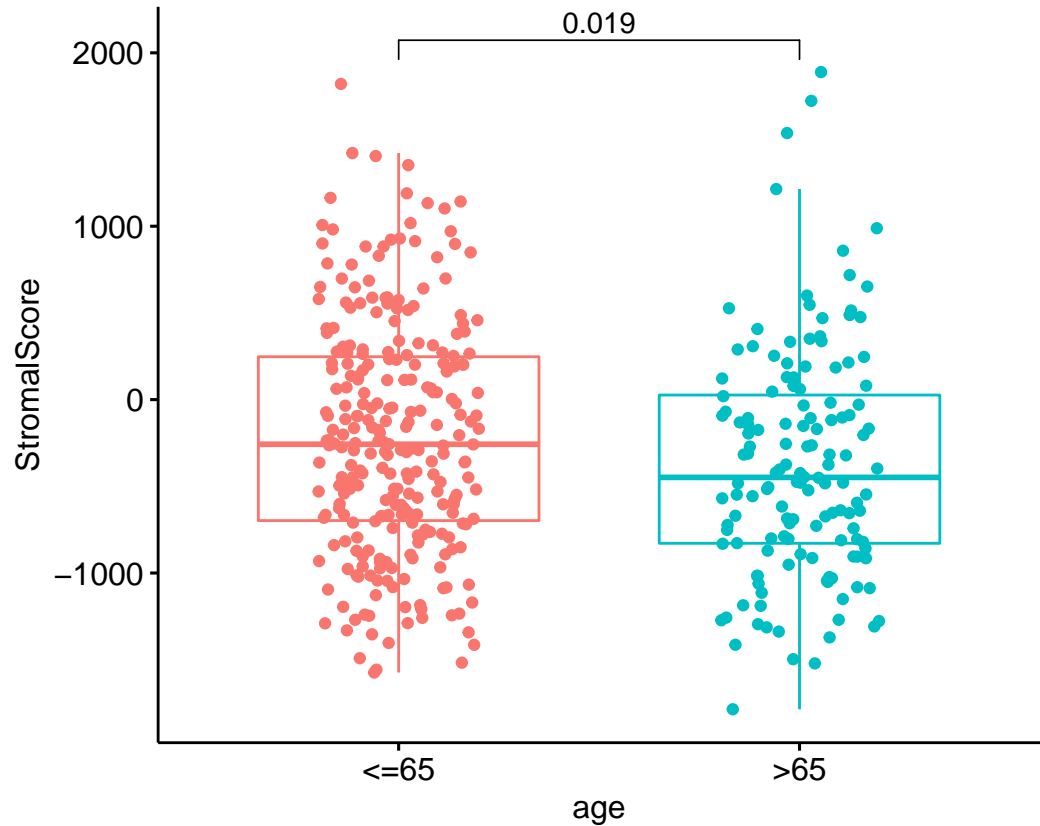

Supplement: S4 File — (ZIP) [file pone.0274897.s006.zip › Step 4.Clinical correlation analysis of the TME/age and Ulceration/output files/StromalScore.age.pdf]

Ulceration 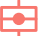 No 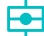 Yes

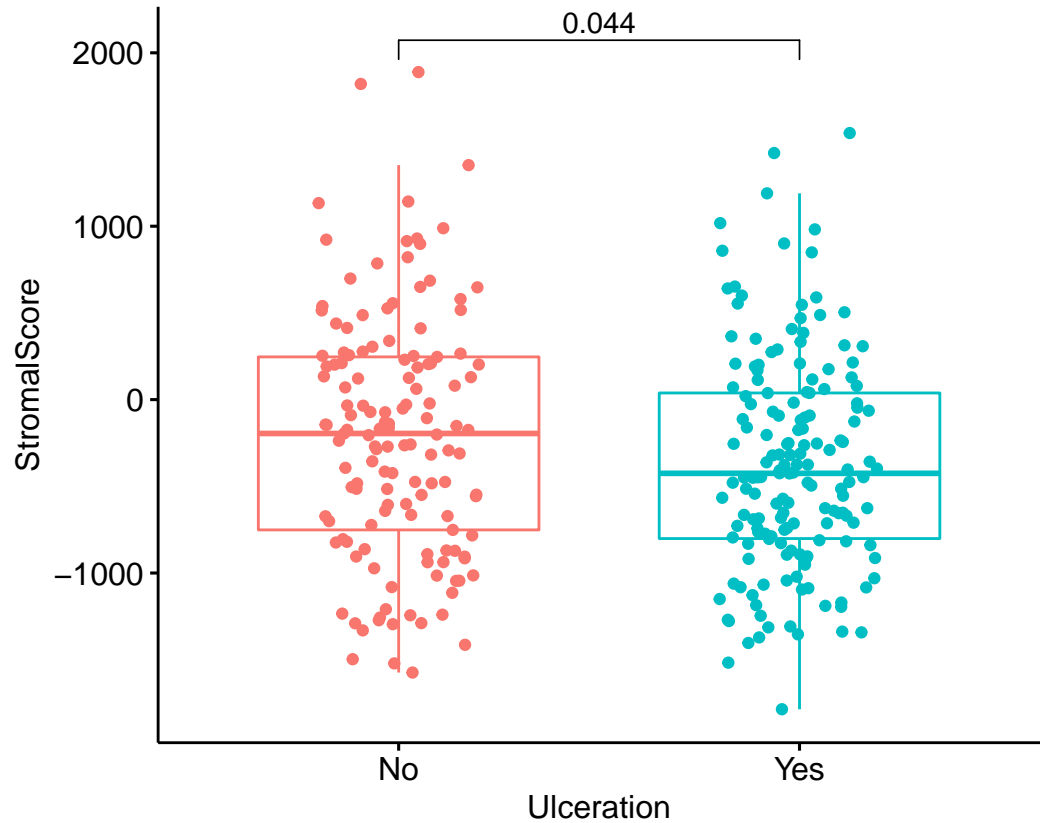

Supplement: S4 File — (ZIP) [file pone.0274897.s006.zip › Step 4.Clinical correlation analysis of the TME/age and Ulceration/output files/StromalScore.Ulceration.pdf]

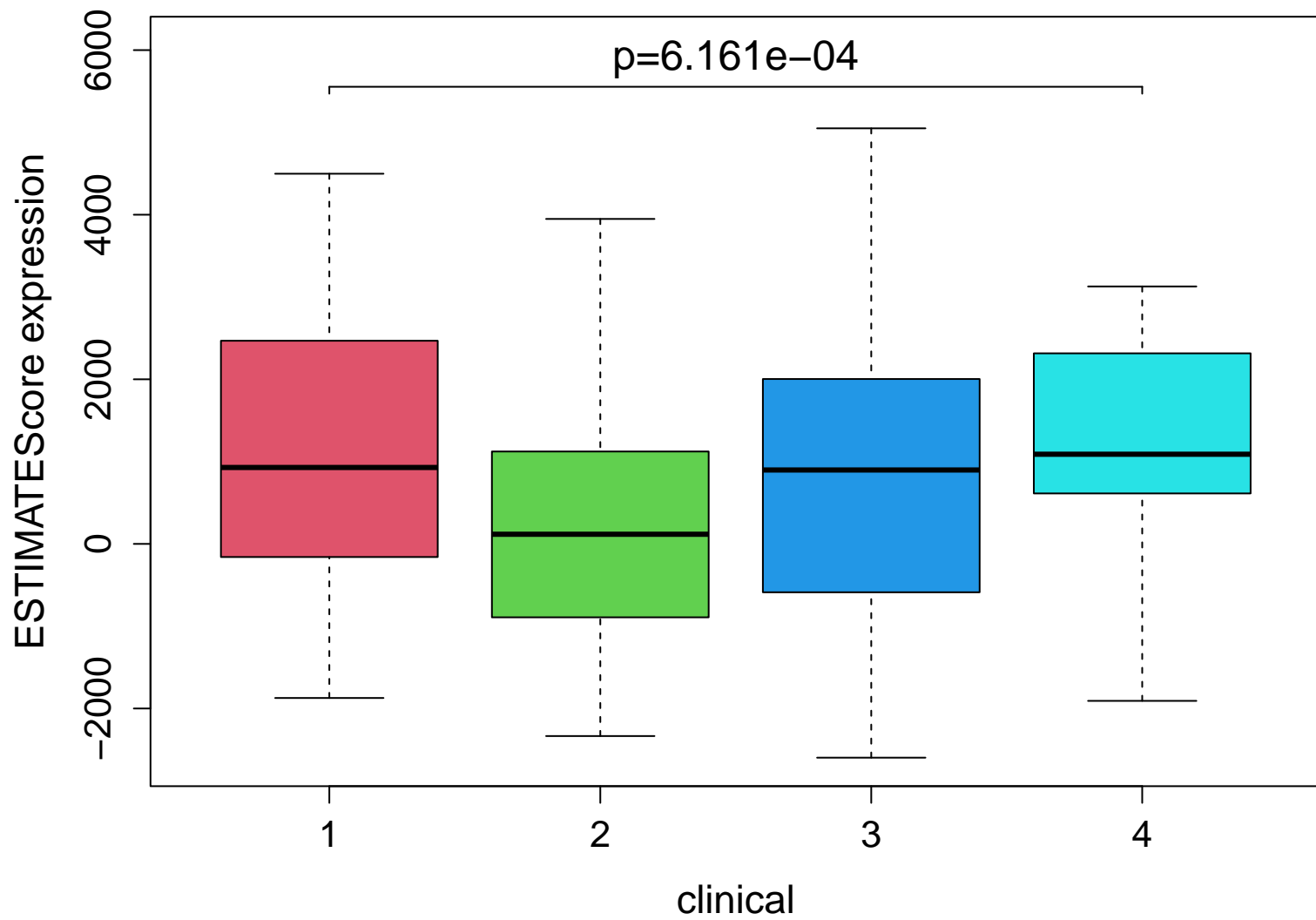

Supplement: S4 File — (ZIP) [file pone.0274897.s006.zip › Step 4.Clinical correlation analysis of the TME/stage/output files/ESTIMATEScore.stage.pdf]

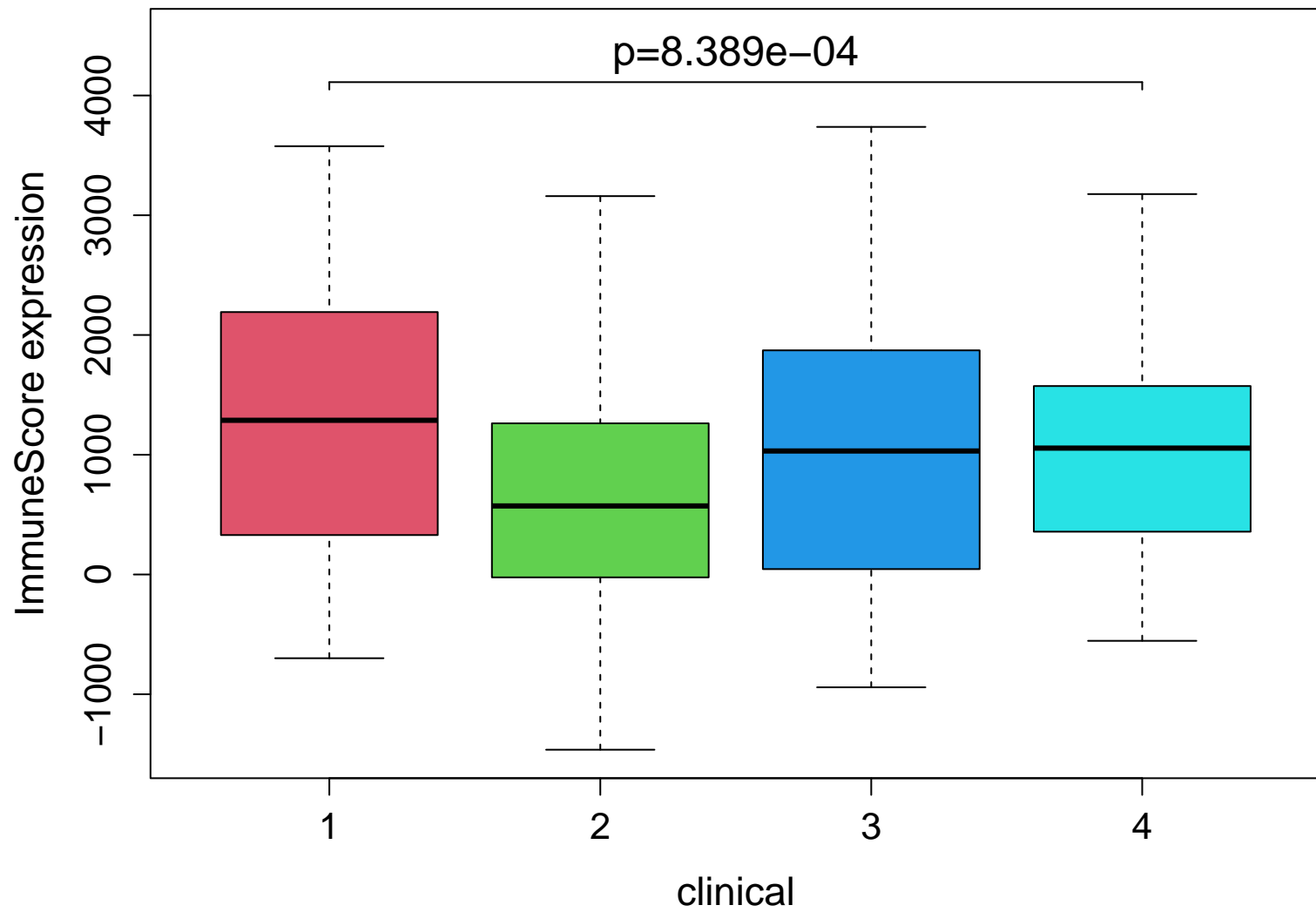

Supplement: S4 File — (ZIP) [file pone.0274897.s006.zip › Step 4.Clinical correlation analysis of the TME/stage/output files/ImmuneScore.stage.pdf]

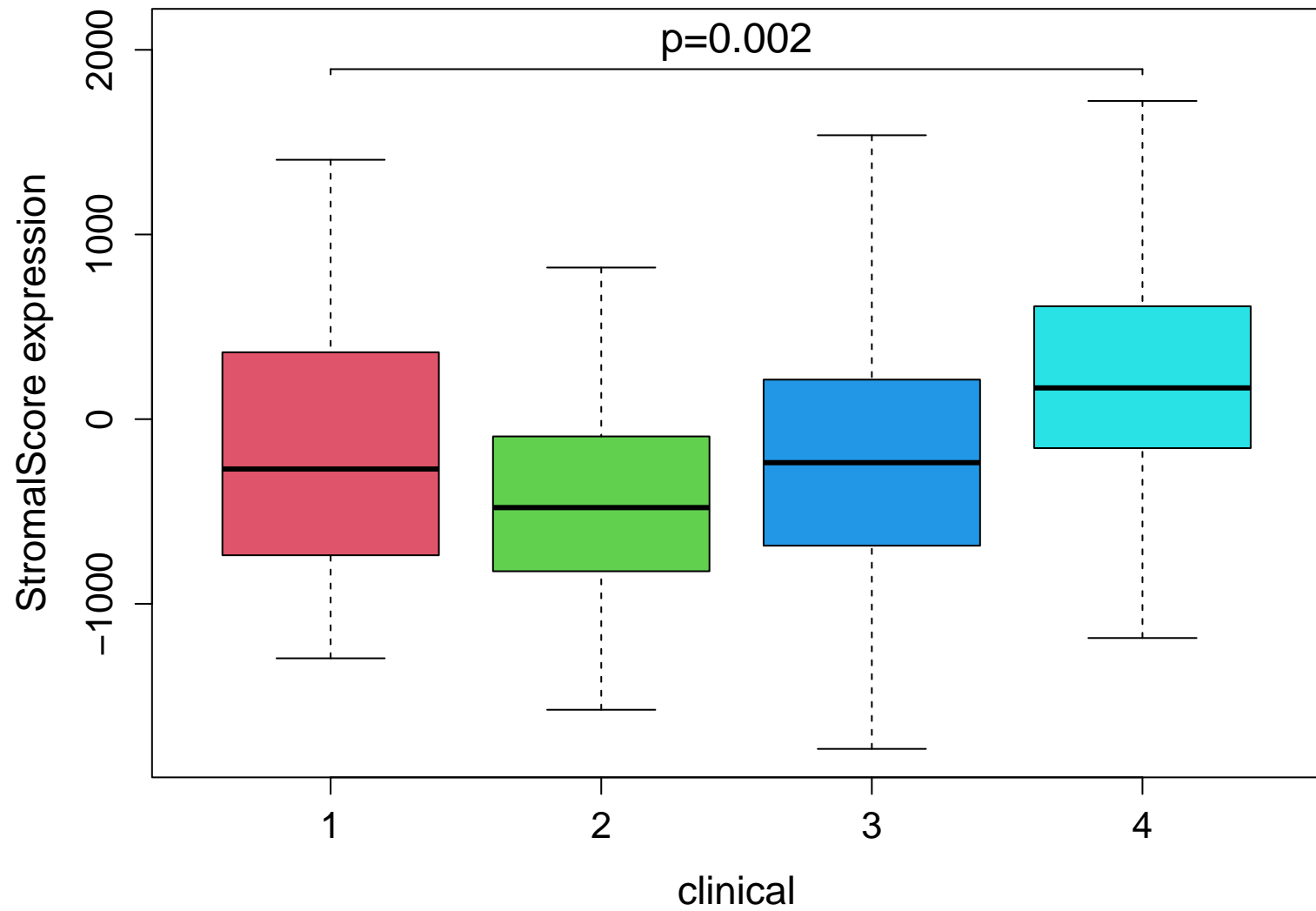

Supplement: S4 File — (ZIP) [file pone.0274897.s006.zip › Step 4.Clinical correlation analysis of the TME/stage/output files/StromalScore.stage.pdf]

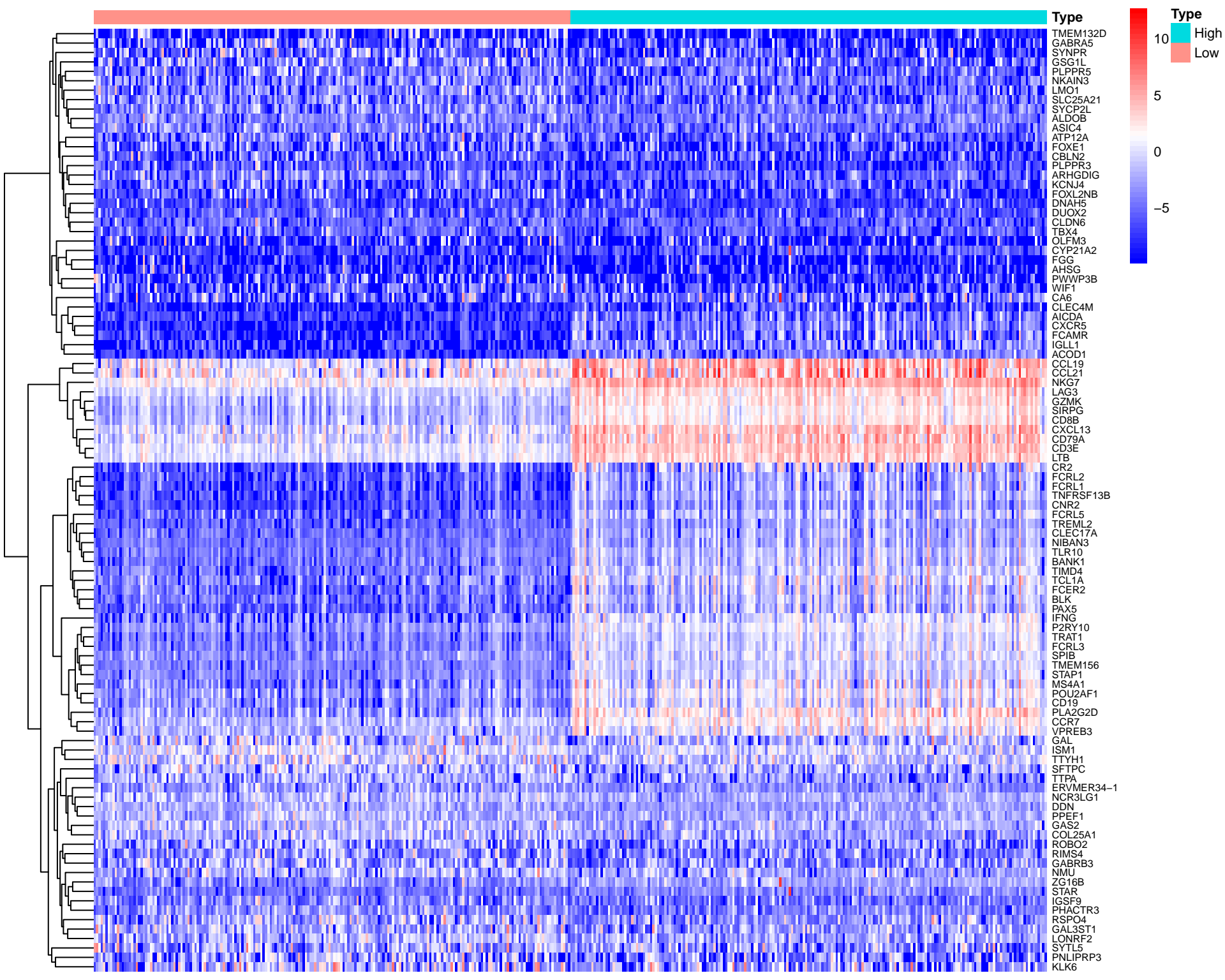

Supplement: S5 File — (ZIP) [file pone.0274897.s007.zip › Step 5.Differentiation analysis/output files/Immune.heatmap.pdf]

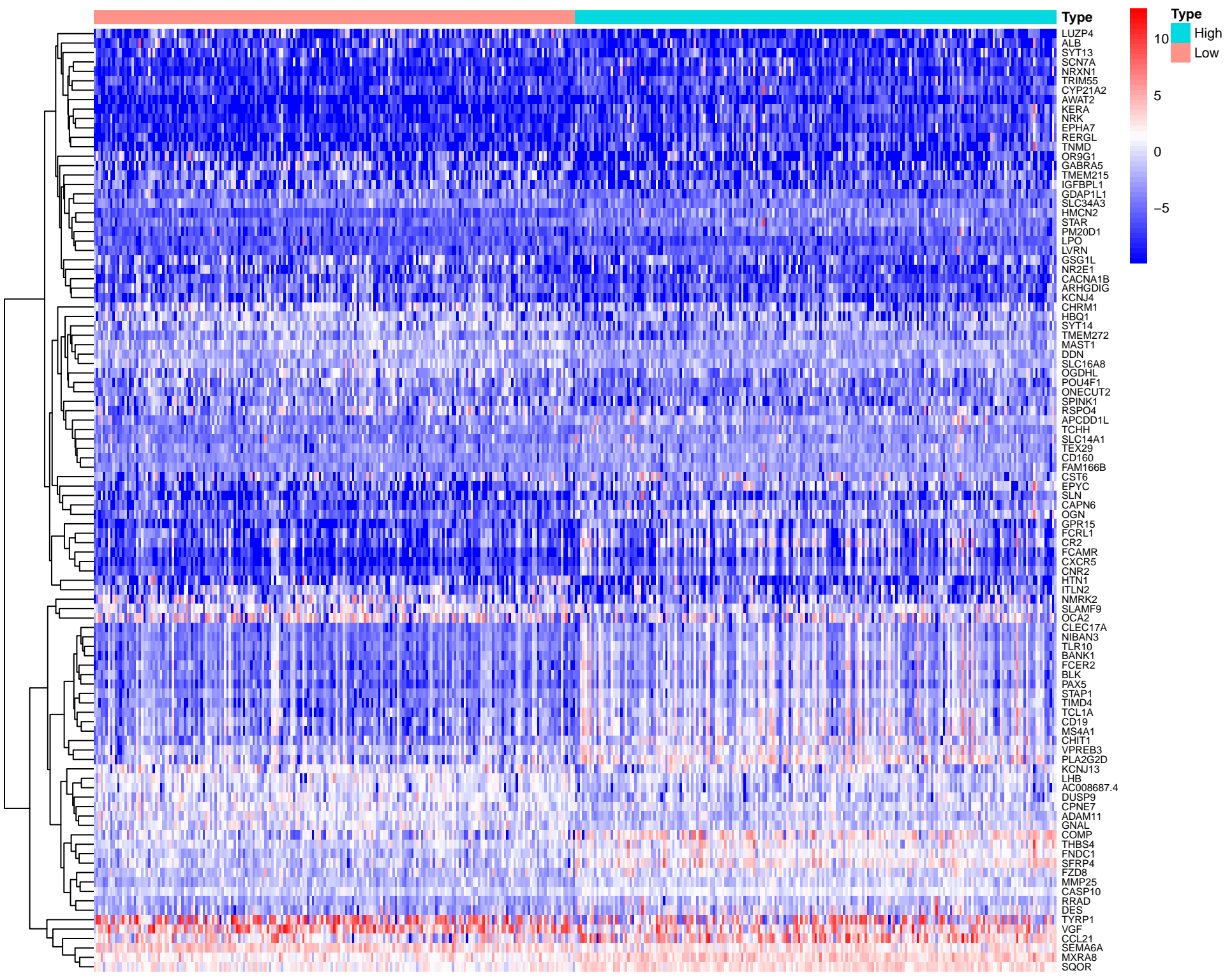

Supplement: S5 File — (ZIP) [file pone.0274897.s007.zip › Step 5.Differentiation analysis/output files/Stromal.heatmap.pdf]

Down

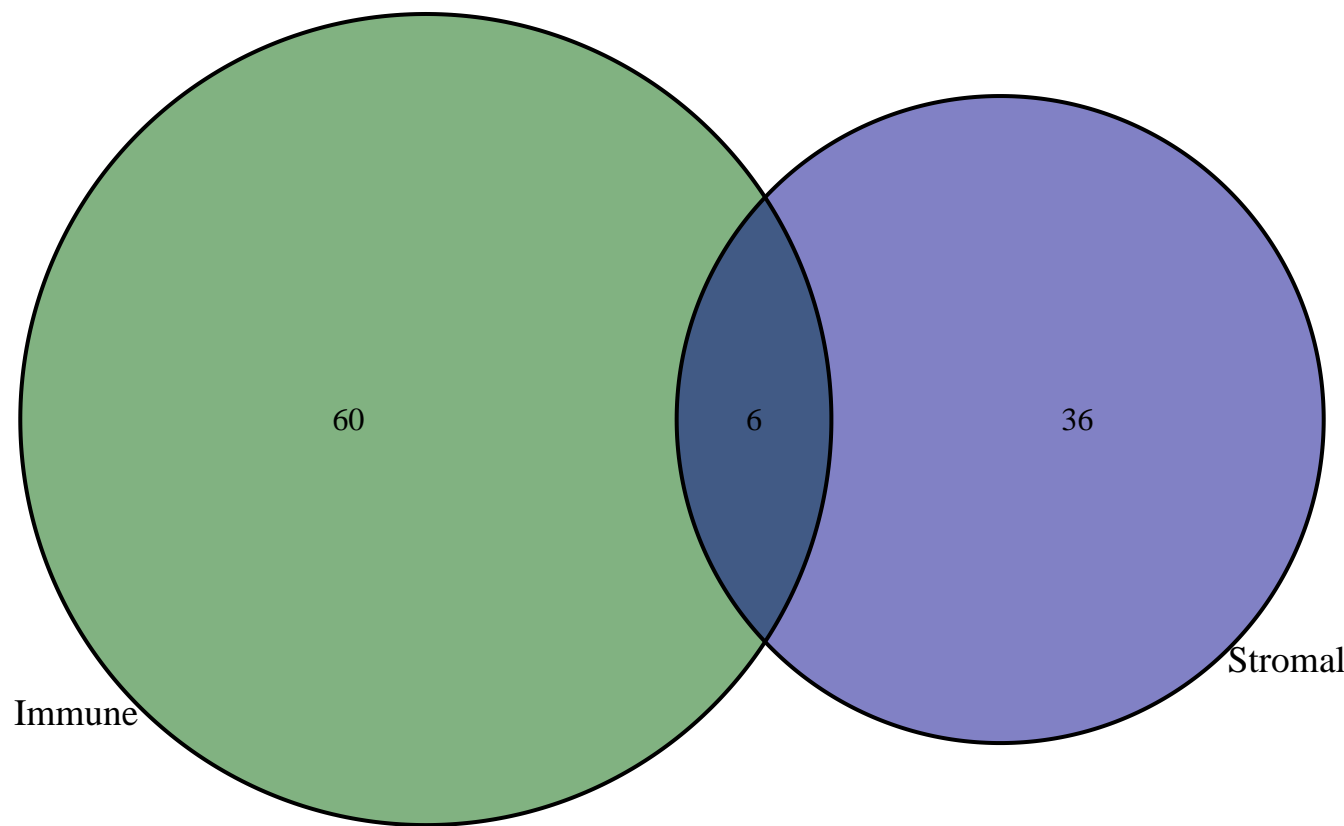

Supplement: S6 File — (ZIP) [file pone.0274897.s008.zip › Step 6.Intersection analysis/output files/DOWN.venn.pdf]

Up

Stromal

Immune

483

908

147

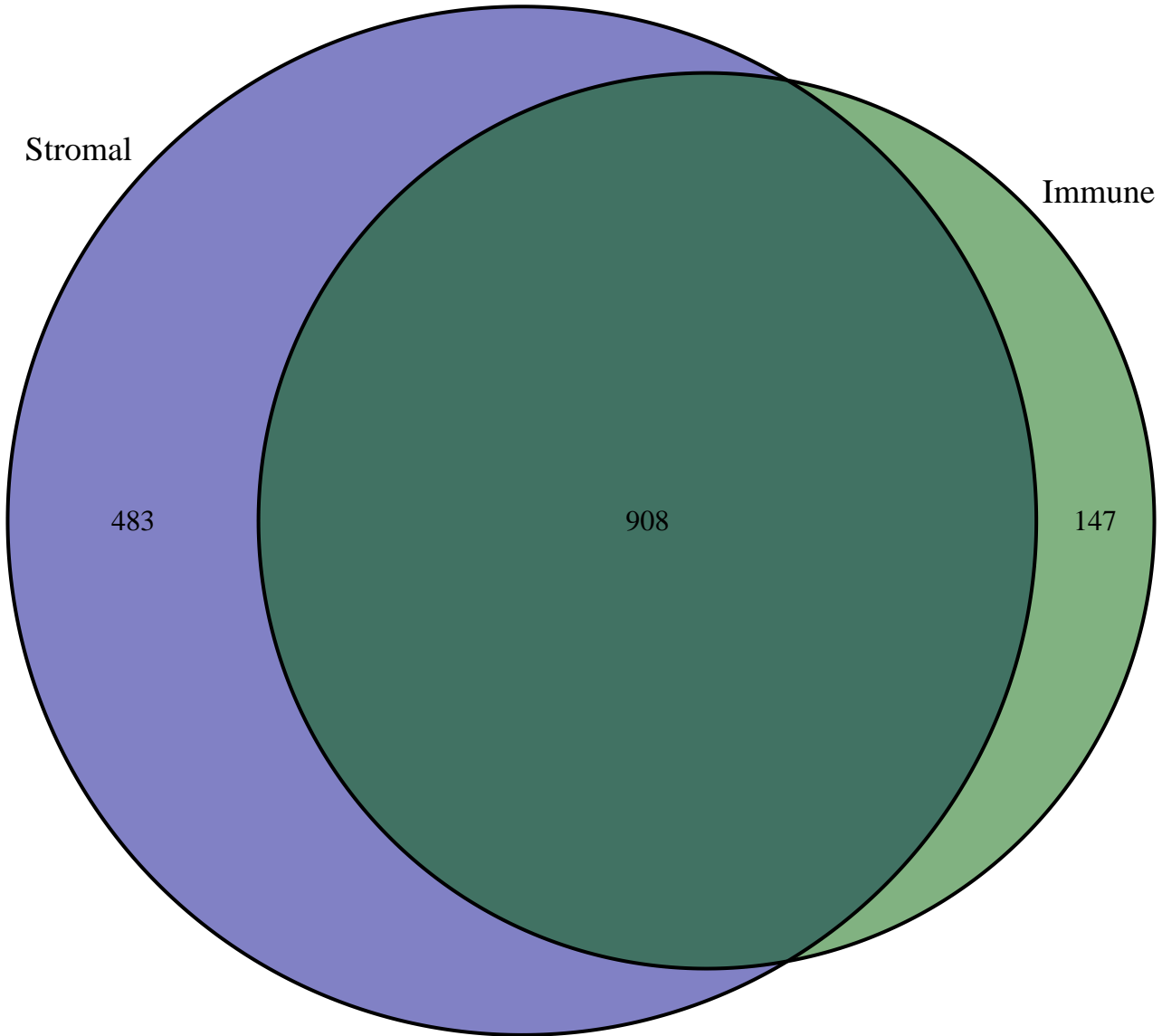

Supplement: S6 File — (ZIP) [file pone.0274897.s008.zip › Step 6.Intersection analysis/output files/UP.venn.pdf]

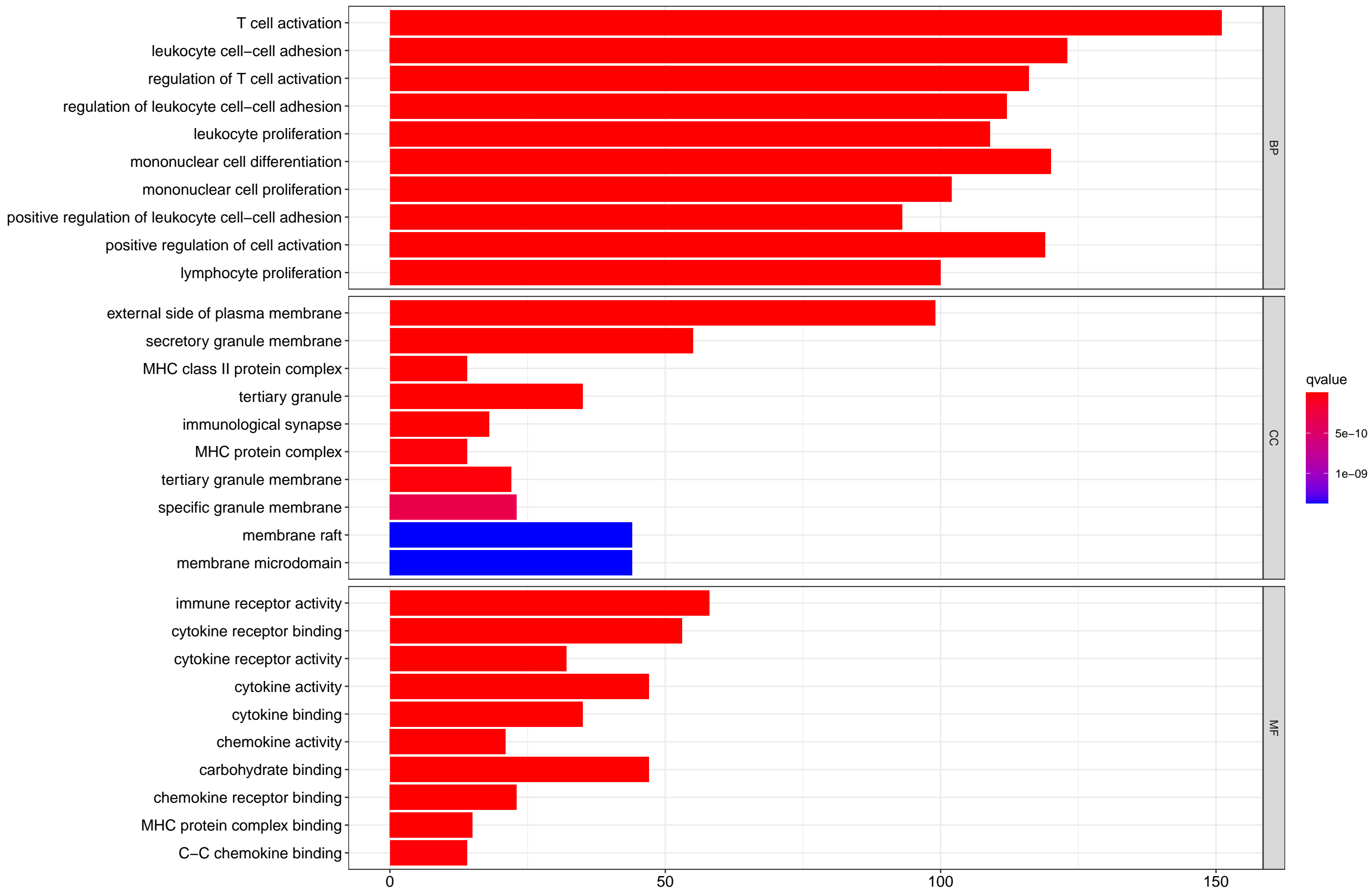

Supplement: S7 File — (ZIP) [file pone.0274897.s009.zip › Step 7.GO and KEGG function enrichment analysis/output files/GObarplot.pdf]

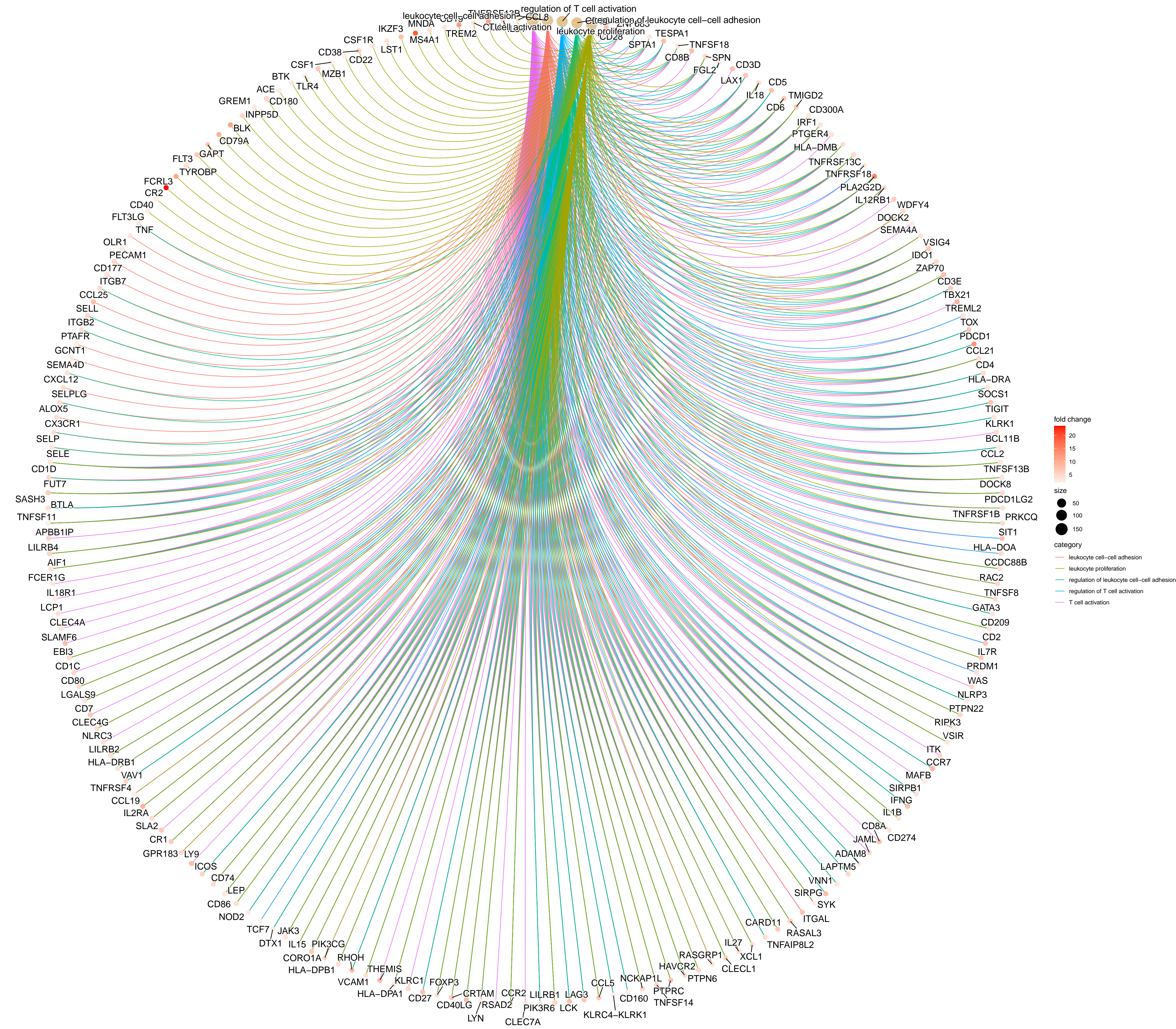

Supplement: S7 File — (ZIP) [file pone.0274897.s009.zip › Step 7.GO and KEGG function enrichment analysis/output files/GOcircos.pdf]

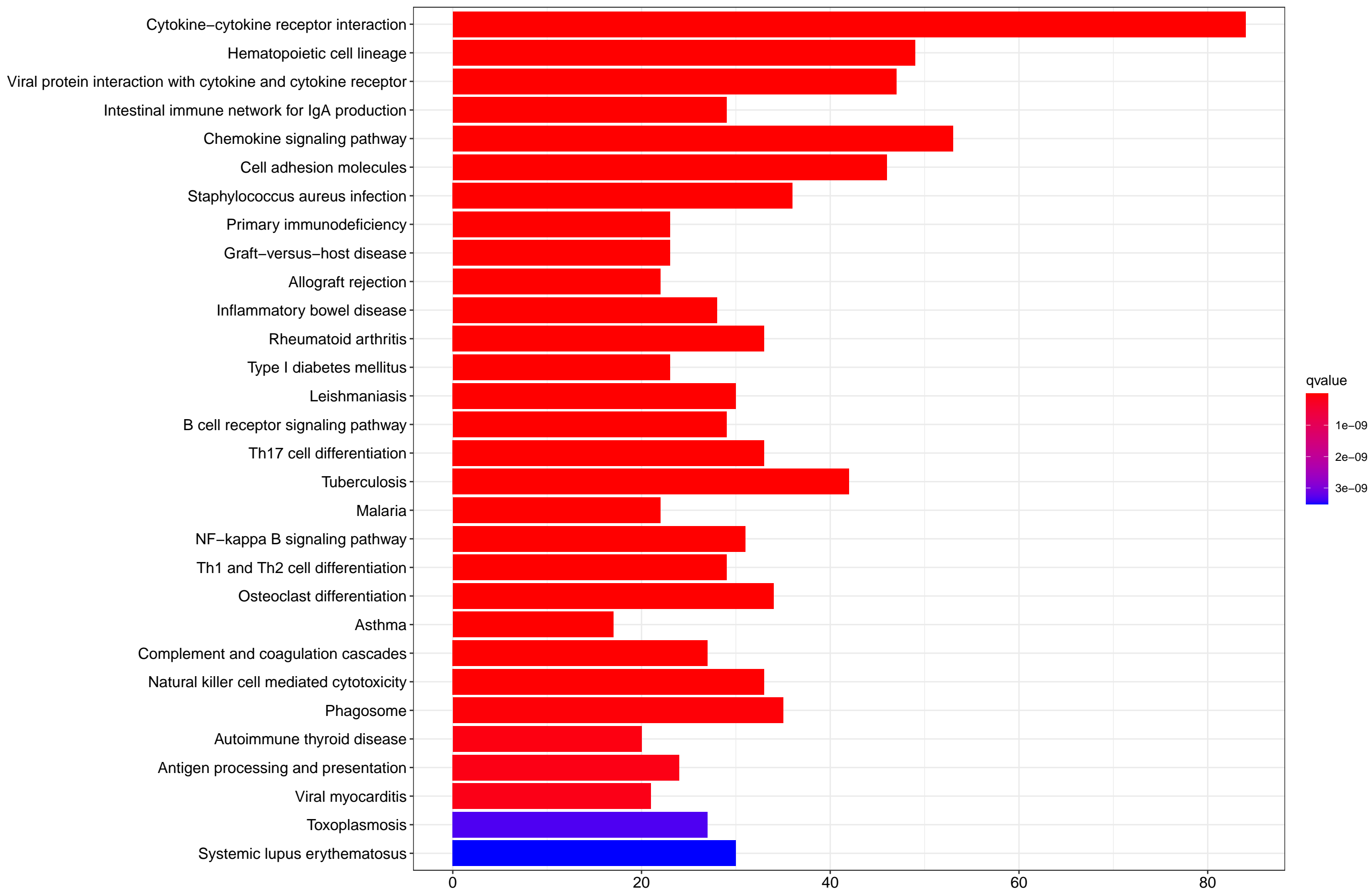

Supplement: S7 File — (ZIP) [file pone.0274897.s009.zip › Step 7.GO and KEGG function enrichment analysis/output files/KEGGbarplot.pdf]

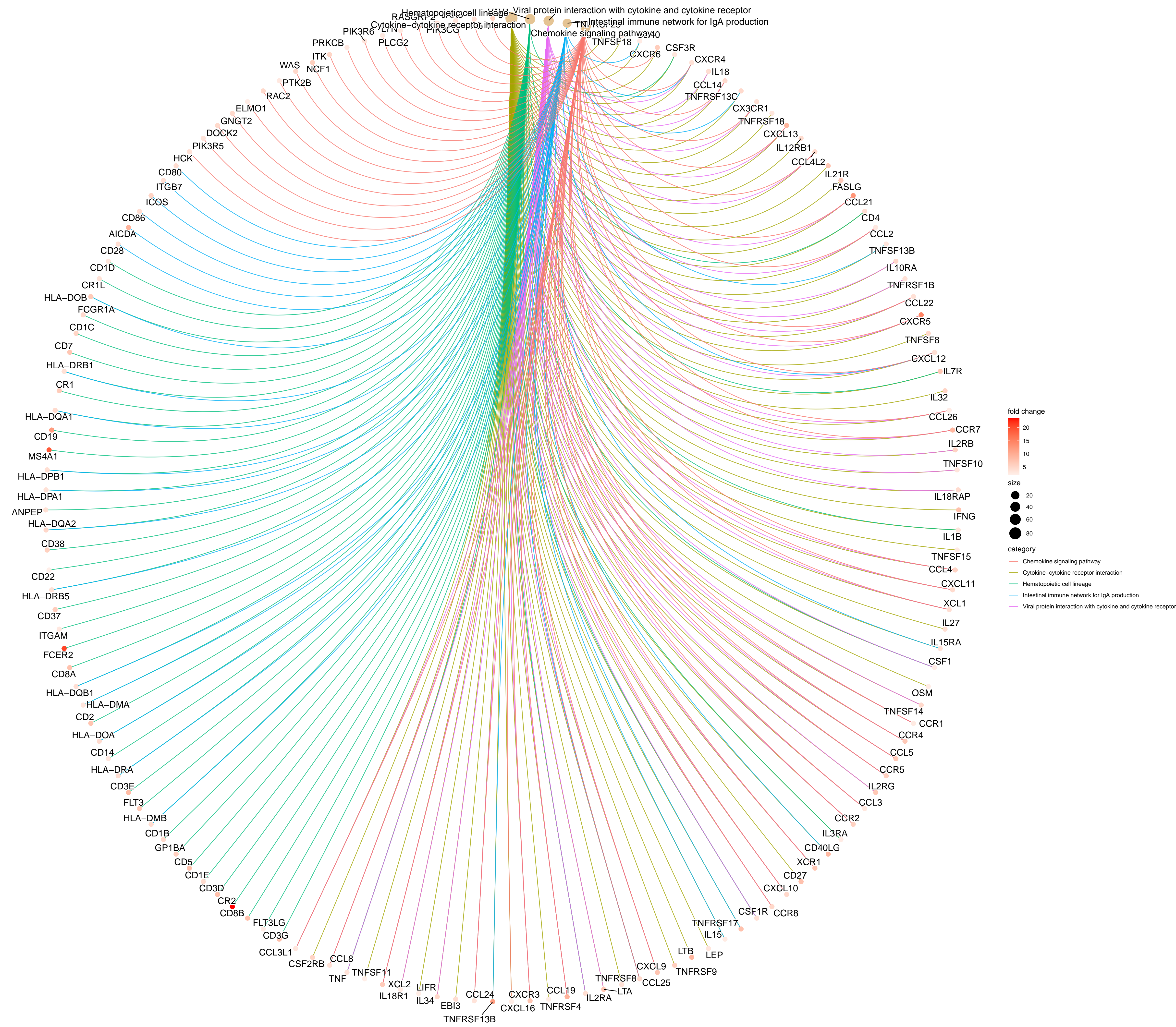

Supplement: S7 File — (ZIP) [file pone.0274897.s009.zip › Step 7.GO and KEGG function enrichment analysis/output files/KEGGcircos.pdf]

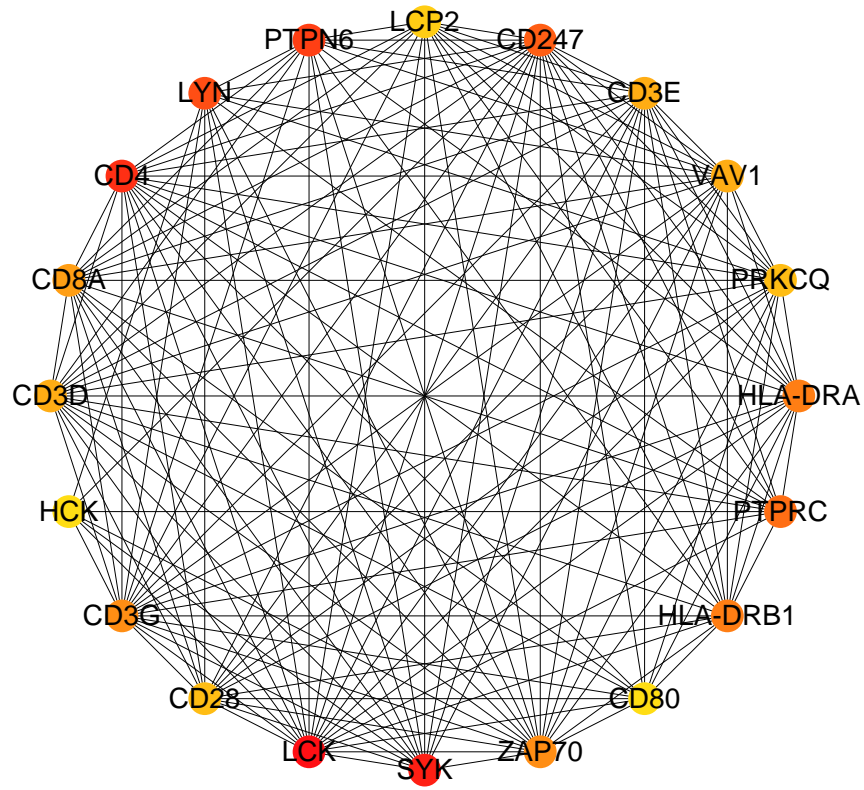

Supplement: S8 File — (ZIP) [file pone.0274897.s010.zip › Step 8.Establishment of protein-protein interaction network/output files/hub20.pdf]

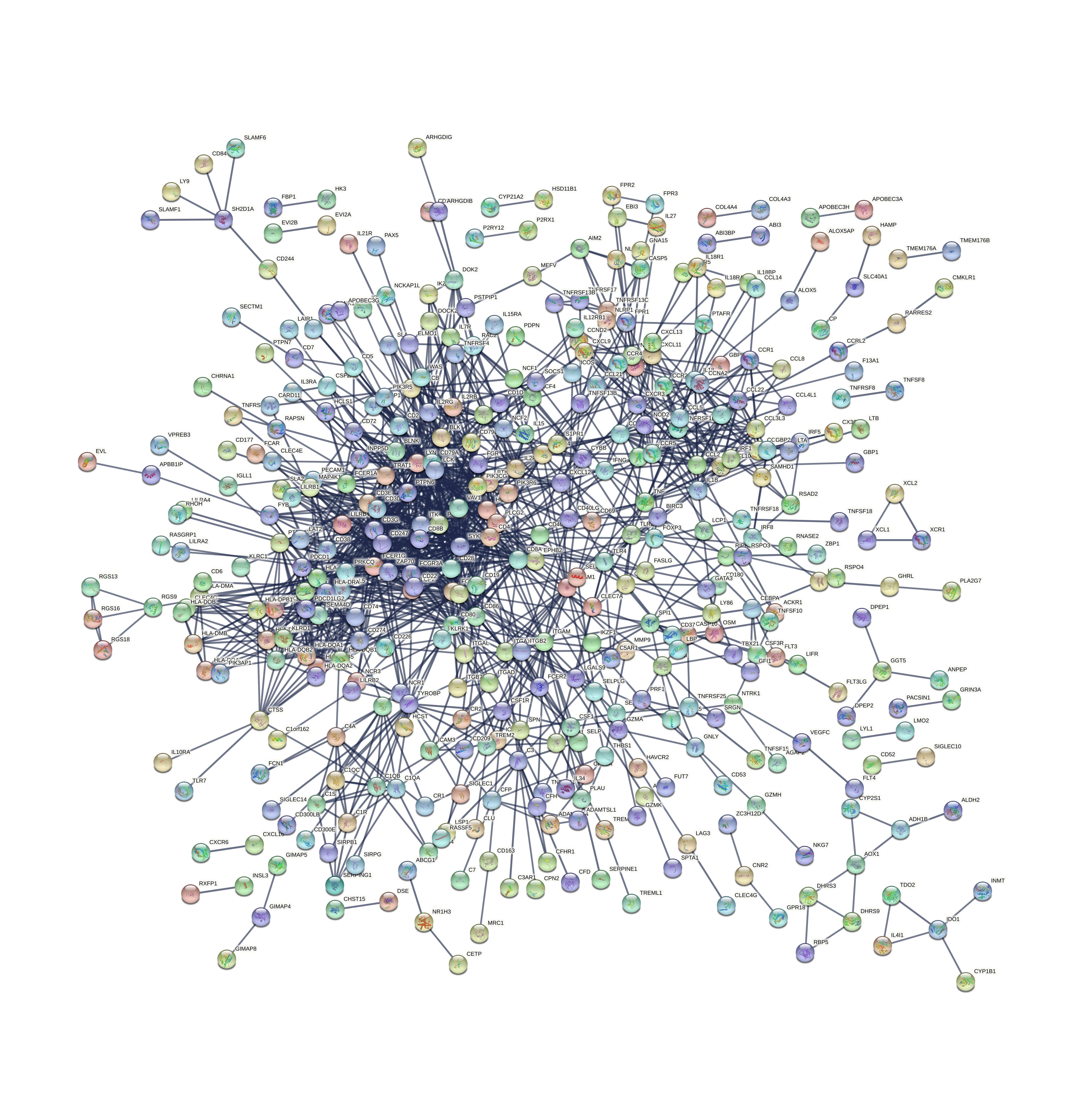

Supplement: S8 File — (ZIP) [file pone.0274897.s010.zip › Step 8.Establishment of protein-protein interaction network/string_hires_image.png]

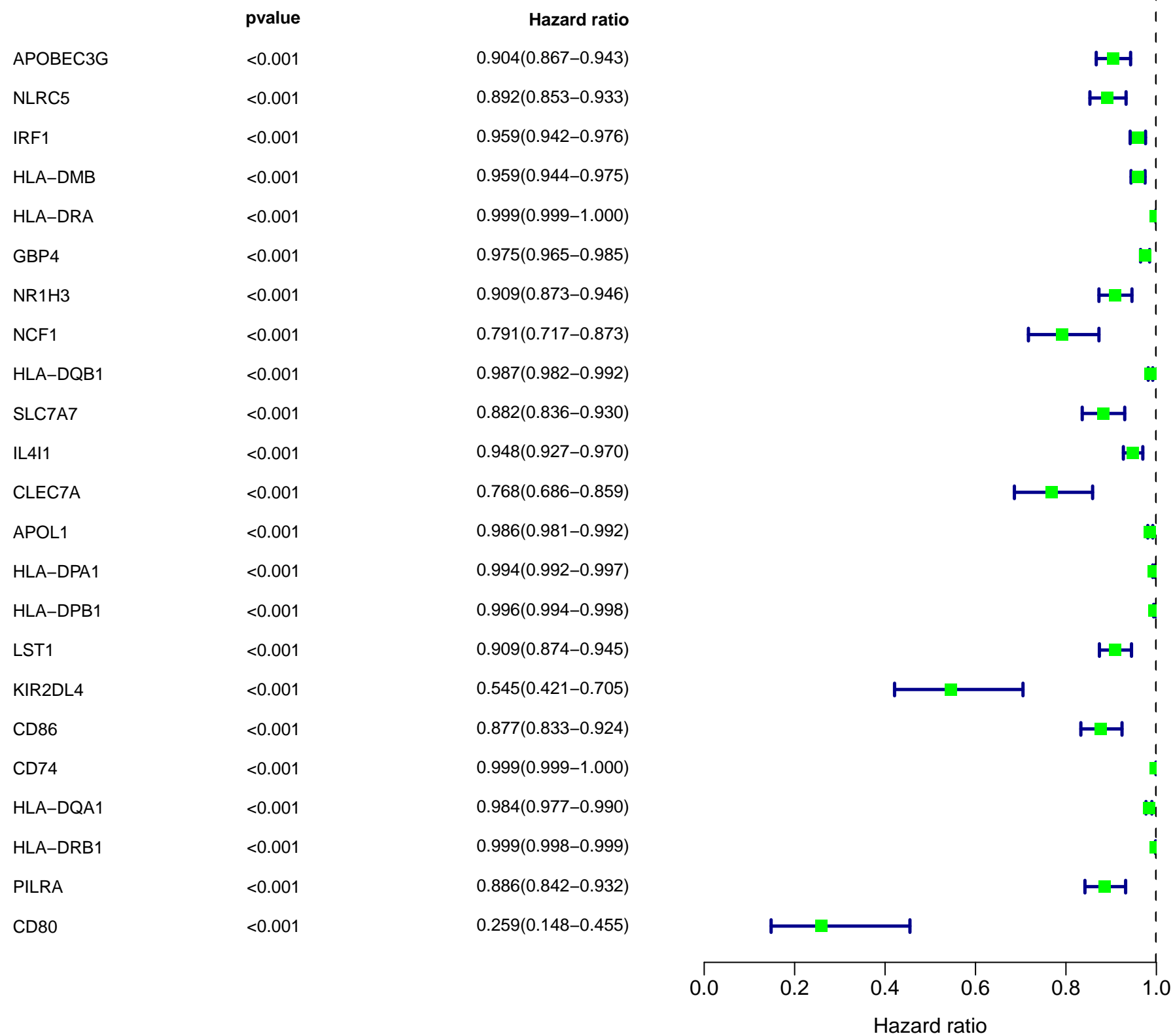

Supplement: S9 File — (ZIP) [file pone.0274897.s011.zip › Step 9.Univariate COX regression analysis/output files/forest.pdf]

# Disease Free Survival

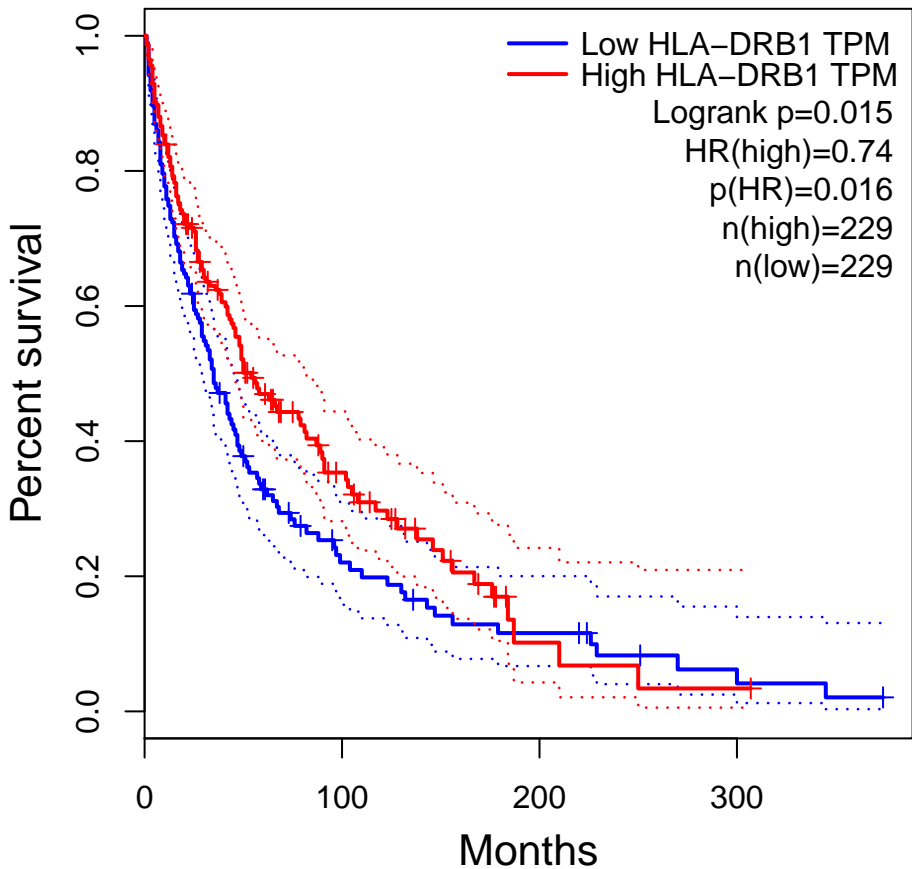

Supplement: S10 File — (ZIP) [file pone.0274897.s012.zip › Step 10.Survival analysis of HLA-DRB1 expression/DFS/DFS.pdf]

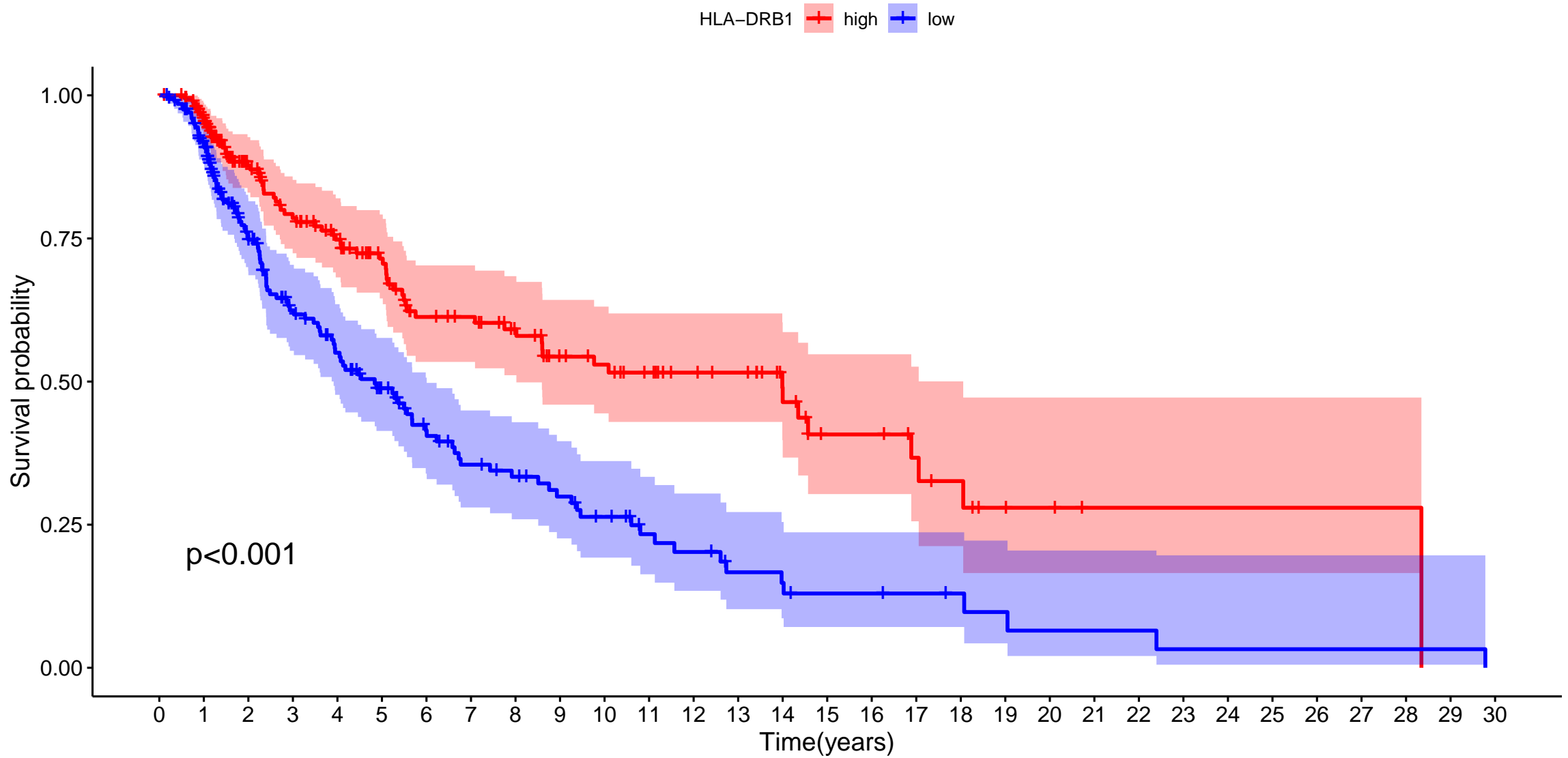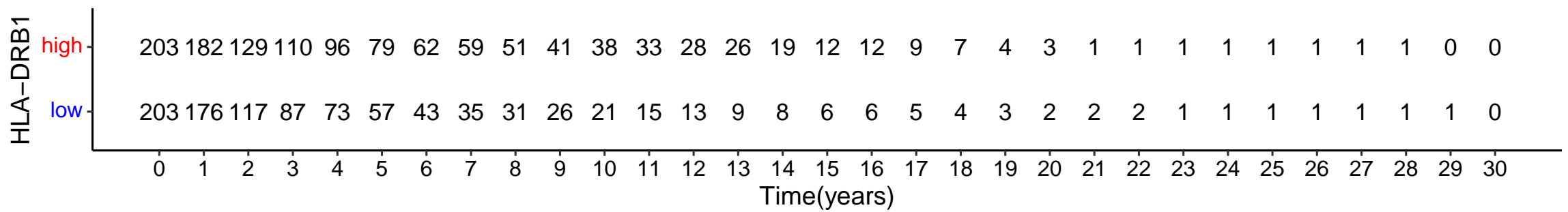

Supplement: S10 File — (ZIP) [file pone.0274897.s012.zip › Step 10.Survival analysis of HLA-DRB1 expression/OS/output file/sur.HLA-DRB1.pdf]

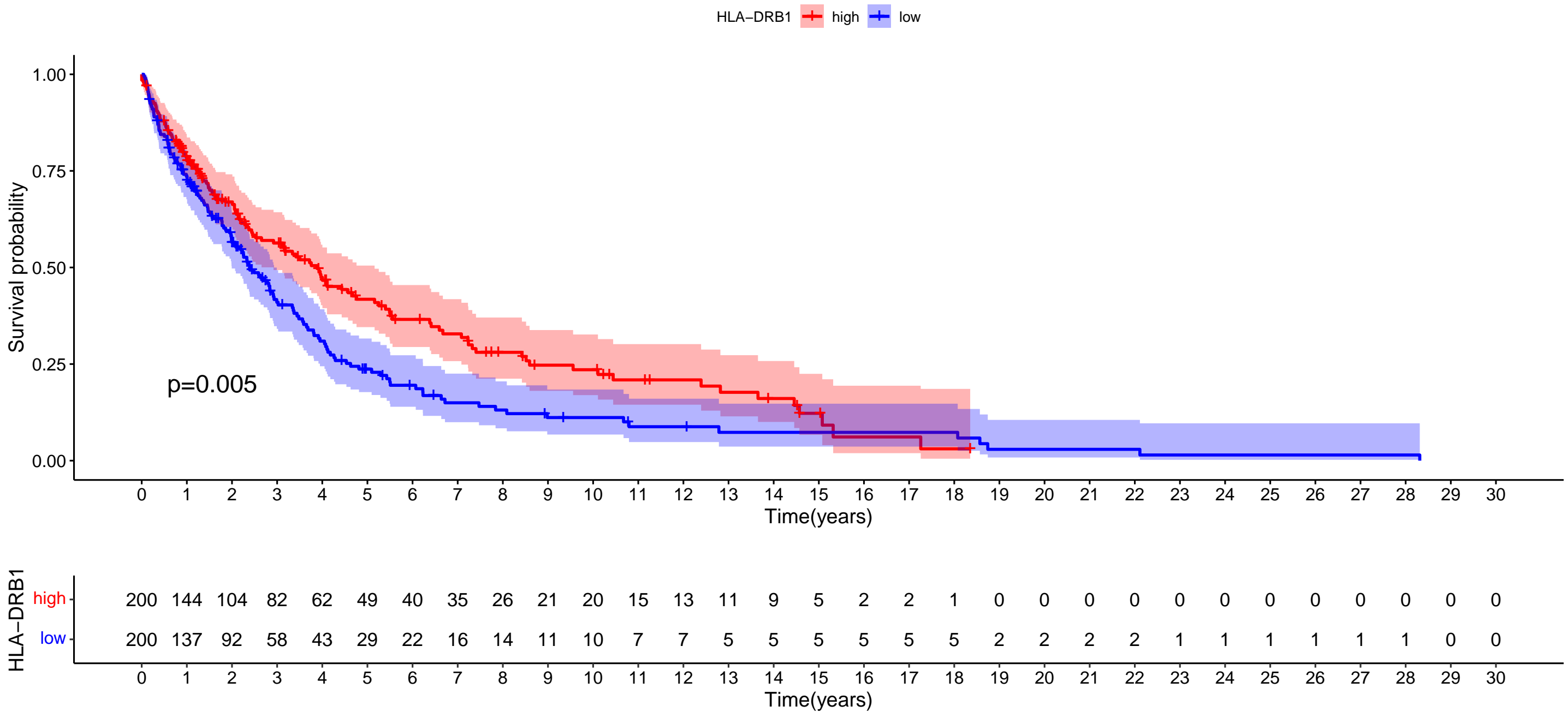

Supplement: S10 File — (ZIP) [file pone.0274897.s012.zip › Step 10.Survival analysis of HLA-DRB1 expression/PFS/output file/PFS.HLA-DRB1.pdf]

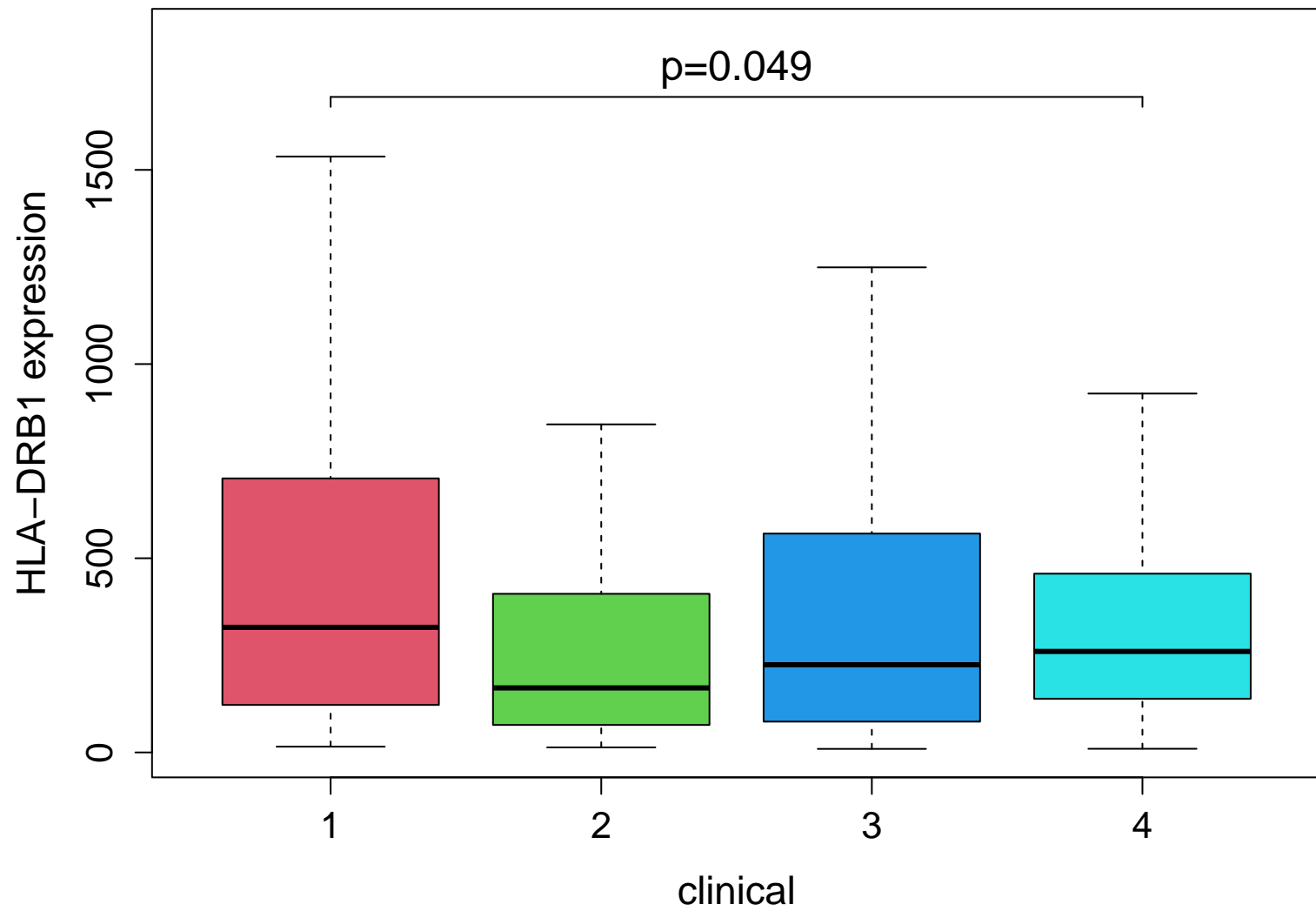

Supplement: S11 File — (ZIP) [file pone.0274897.s013.zip › Step 11.Correlation analysis between Stage and HLA-DRB1 expression/output file/HLA-DRB1.stage.pdf]

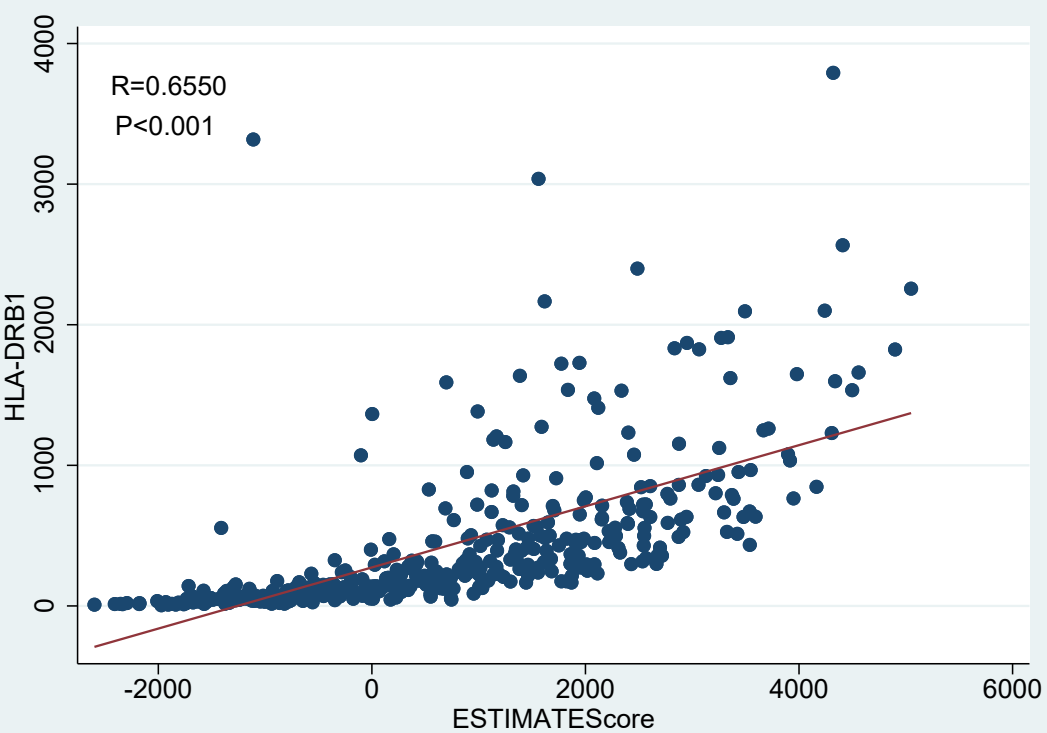

Supplement: S12 File — (ZIP) [file pone.0274897.s014.zip › Step 12.Correlation analysis between TME and HLA-DRB1 expression/output files/ESTIMATEScore.pdf]

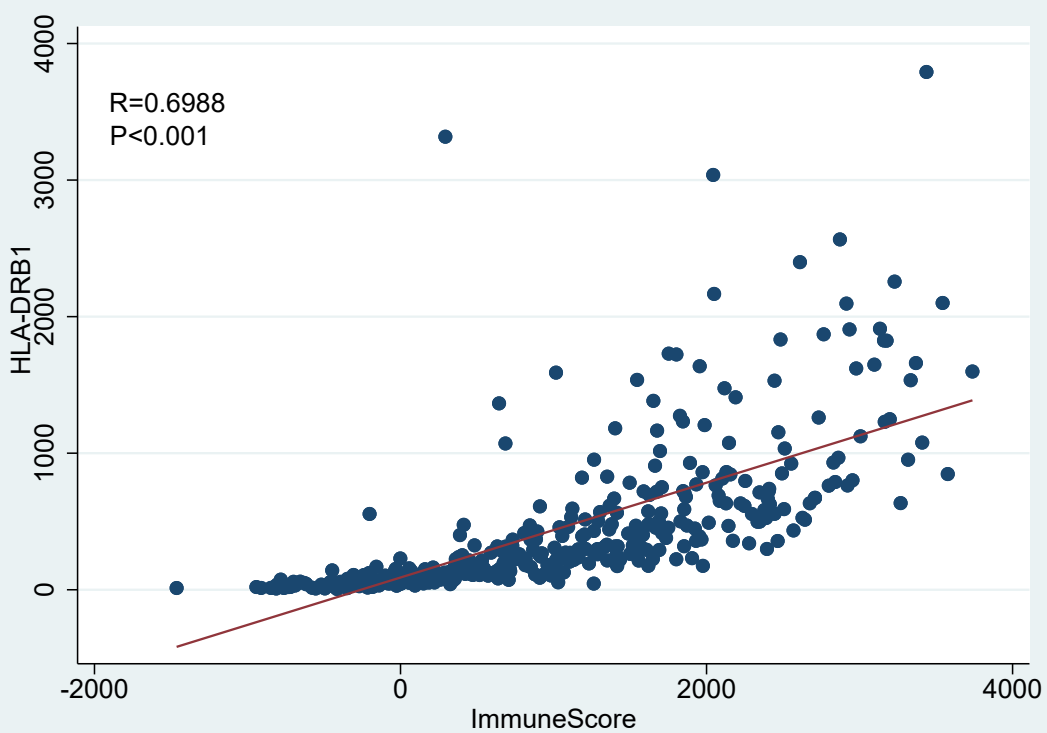

Supplement: S12 File — (ZIP) [file pone.0274897.s014.zip › Step 12.Correlation analysis between TME and HLA-DRB1 expression/output files/ImmuneScore.pdf]

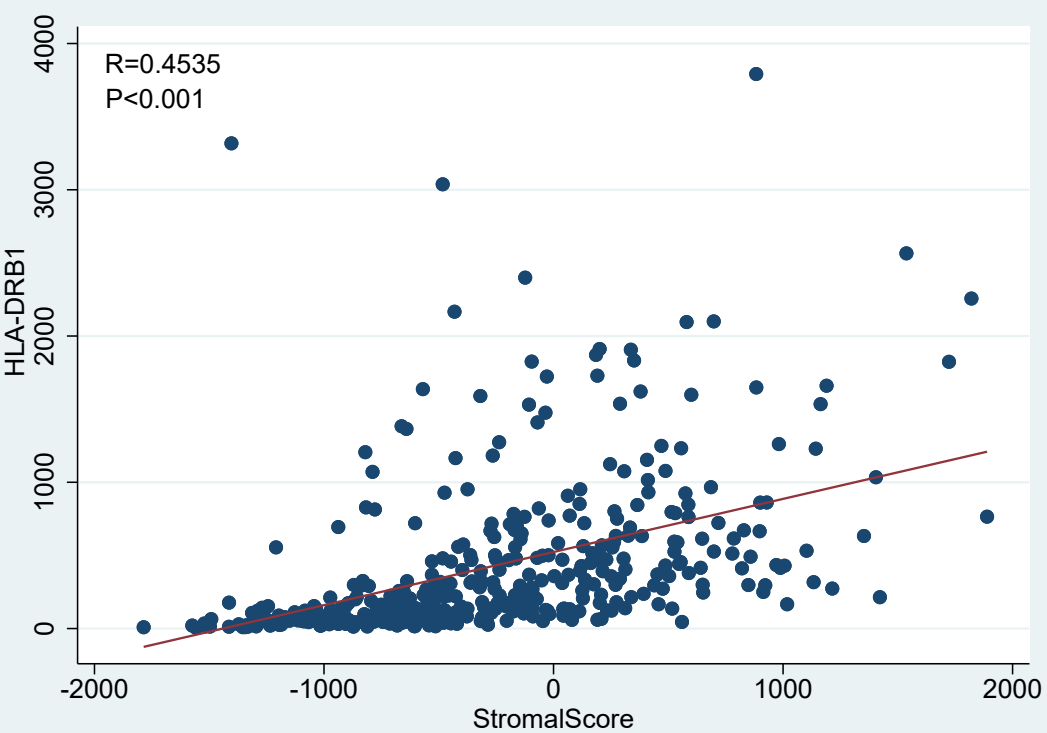

Supplement: S12 File — (ZIP) [file pone.0274897.s014.zip › Step 12.Correlation analysis between TME and HLA-DRB1 expression/output files/StromalScore.pdf]

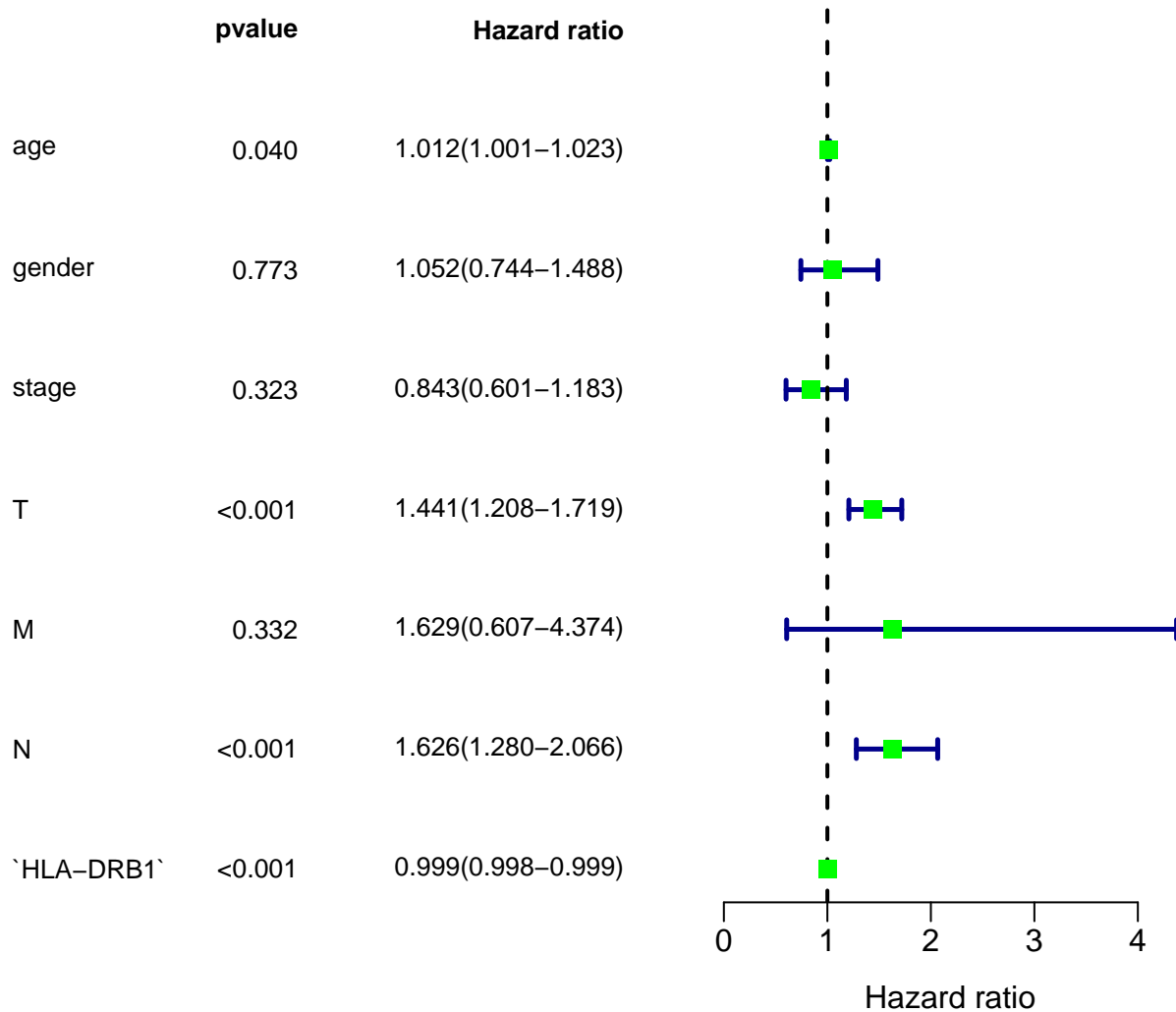

Supplement: S13 File — (ZIP) [file pone.0274897.s015.zip › Step 13.Independent prognostic analysis/output file/multiCoxforest.pdf]

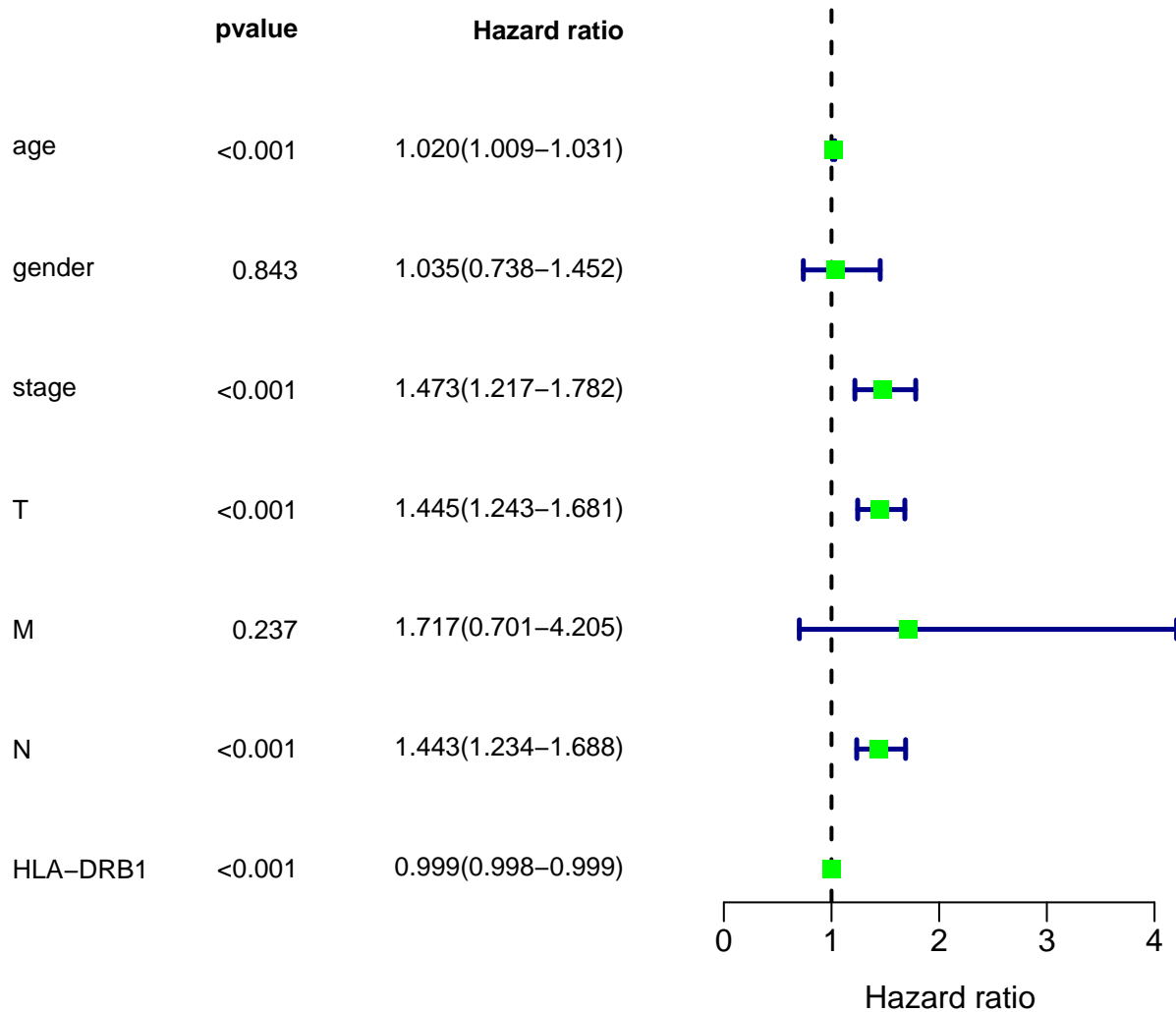

Supplement: S13 File — (ZIP) [file pone.0274897.s015.zip › Step 13.Independent prognostic analysis/output file/uniCoxforest.pdf]

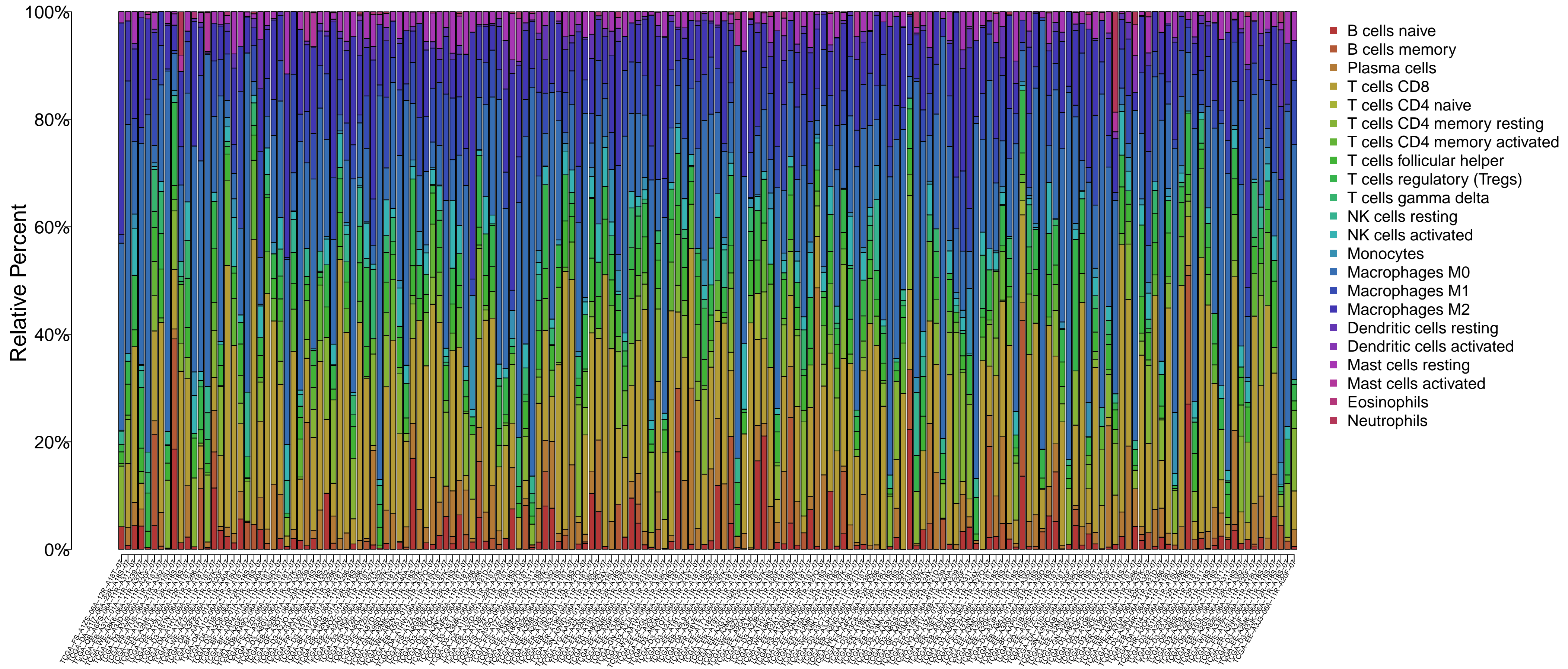

Supplement: S14 File — (ZIP) [file pone.0274897.s016.zip › Step 14.TIC abundance profile/output file/barplot.pdf]

Dendritic cells activated

$R = -0.18, p = 0.018$

0.050

0.025

0.000

0

1000

2000

3000

HLA-DRB1

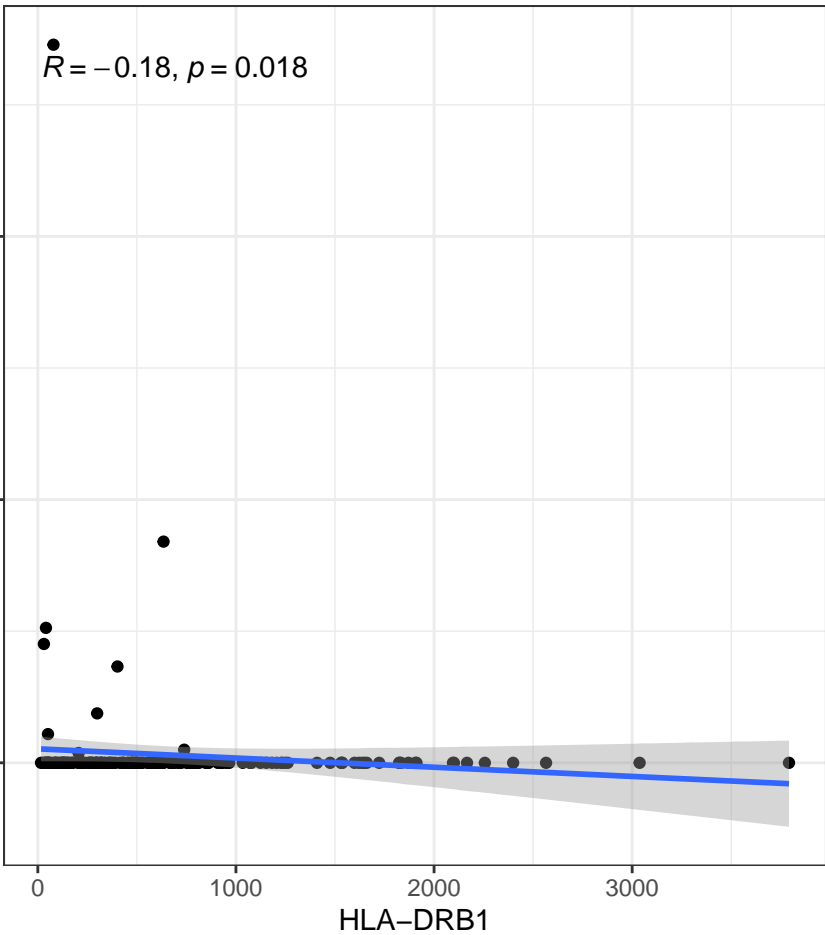

Supplement: S15 File — (ZIP) [file pone.0274897.s017.zip › Step 15.Immune cell differentiation analysis and correlation analysis/output files/Dendritic cells activated.pdf]

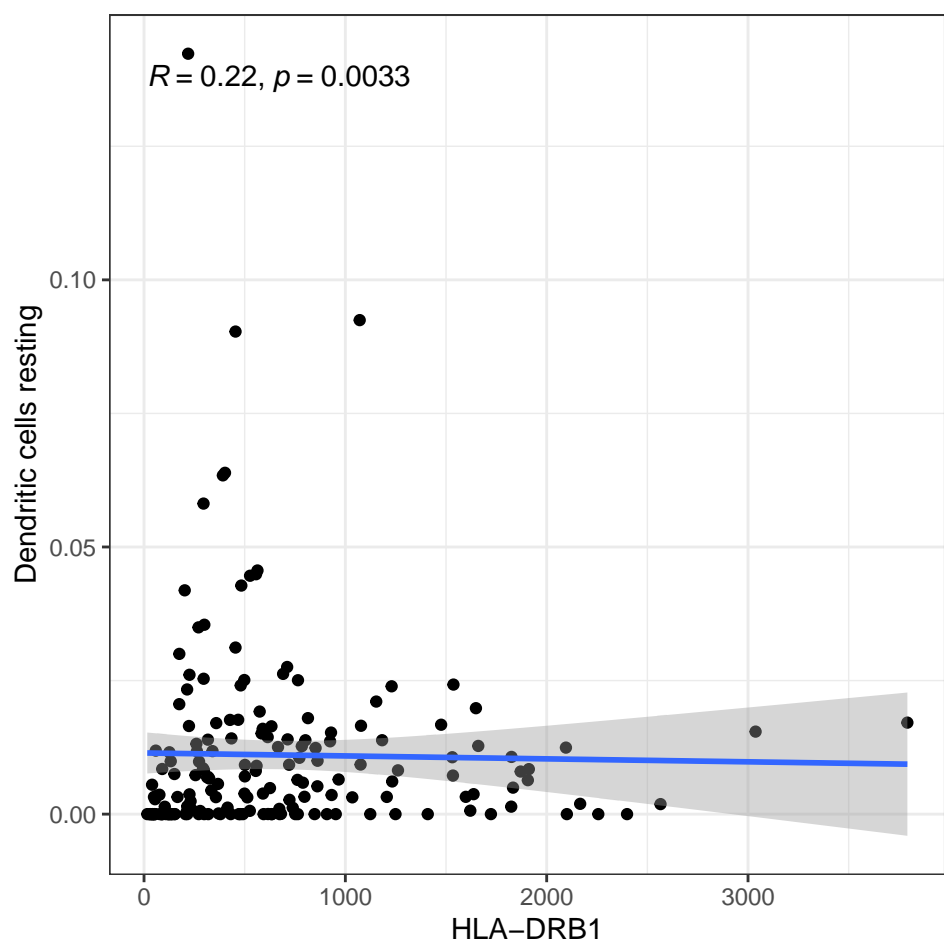

Supplement: S15 File — (ZIP) [file pone.0274897.s017.zip › Step 15.Immune cell differentiation analysis and correlation analysis/output files/Dendritic cells resting.pdf]

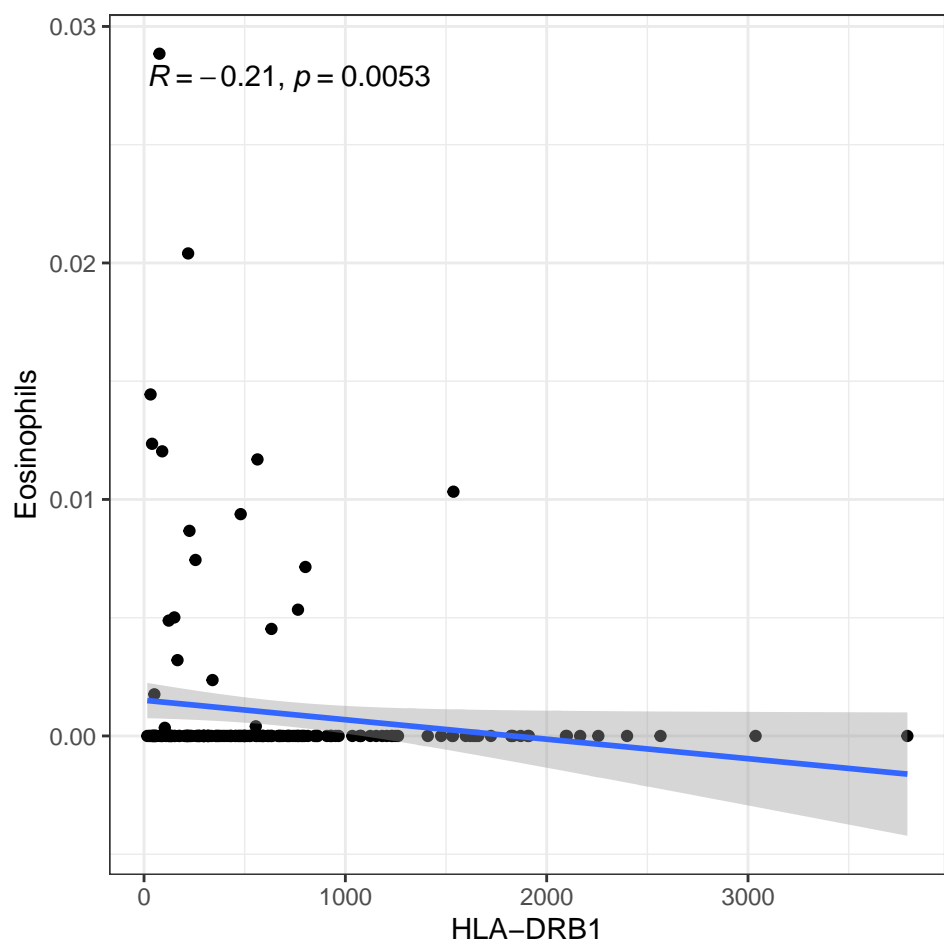

Supplement: S15 File — (ZIP) [file pone.0274897.s017.zip › Step 15.Immune cell differentiation analysis and correlation analysis/output files/Eosinophils.pdf]

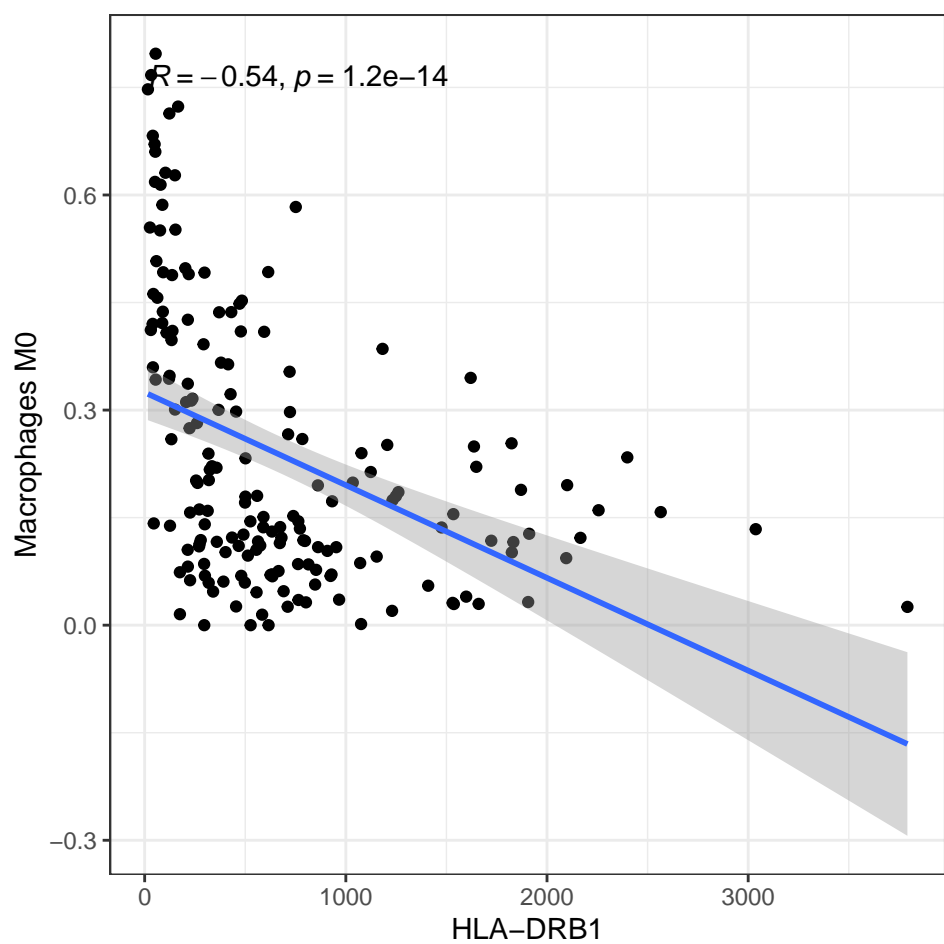

Supplement: S15 File — (ZIP) [file pone.0274897.s017.zip › Step 15.Immune cell differentiation analysis and correlation analysis/output files/Macrophages M0.pdf]

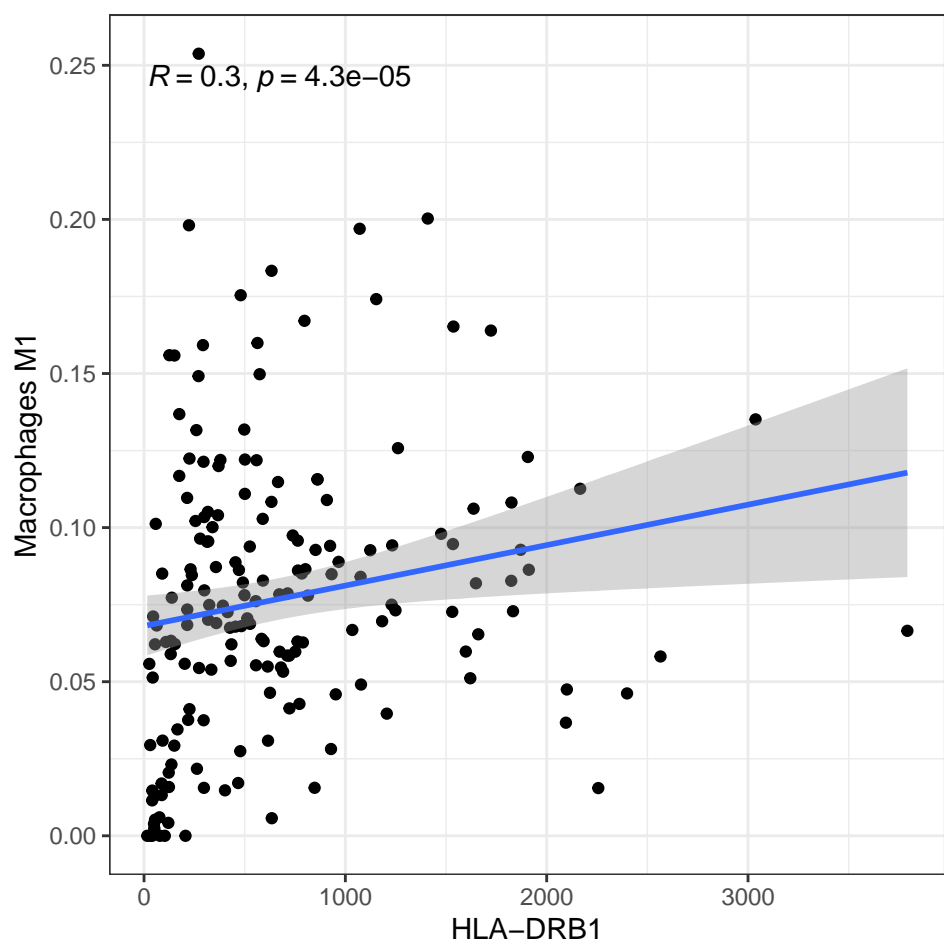

Supplement: S15 File — (ZIP) [file pone.0274897.s017.zip › Step 15.Immune cell differentiation analysis and correlation analysis/output files/Macrophages M1.pdf]

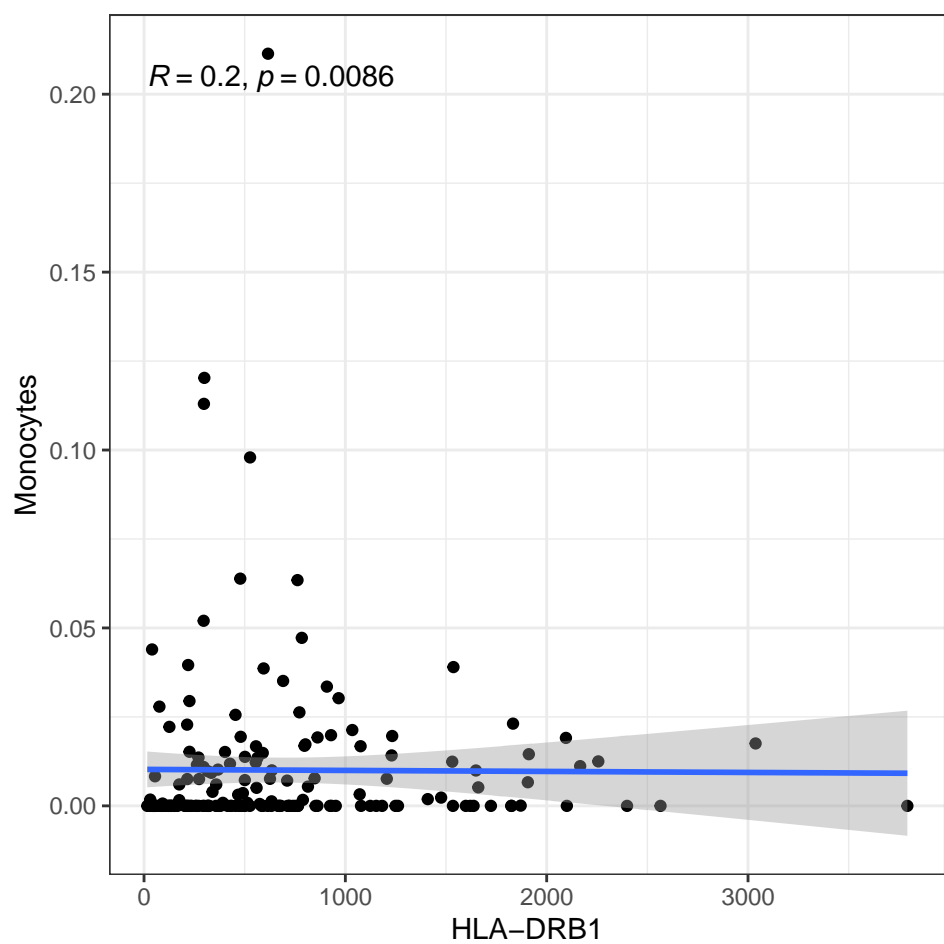

Supplement: S15 File — (ZIP) [file pone.0274897.s017.zip › Step 15.Immune cell differentiation analysis and correlation analysis/output files/Monocytes.pdf]

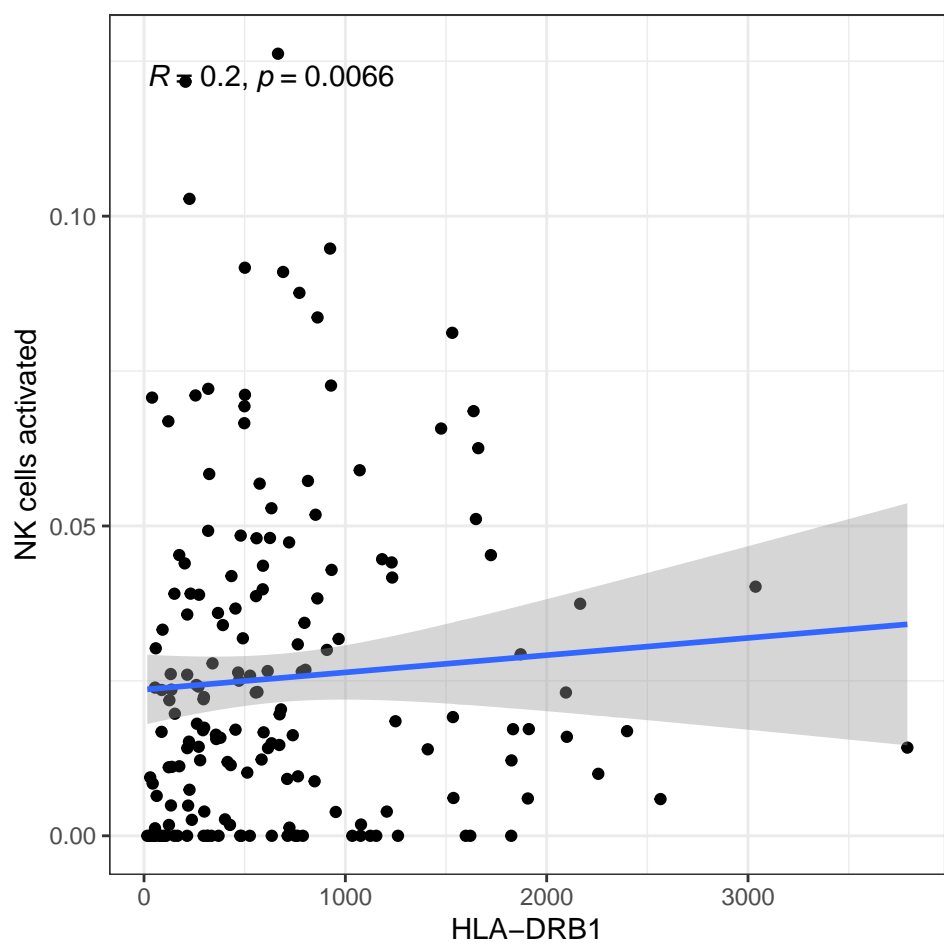

Supplement: S15 File — (ZIP) [file pone.0274897.s017.zip › Step 15.Immune cell differentiation analysis and correlation analysis/output files/NK cells activated.pdf]

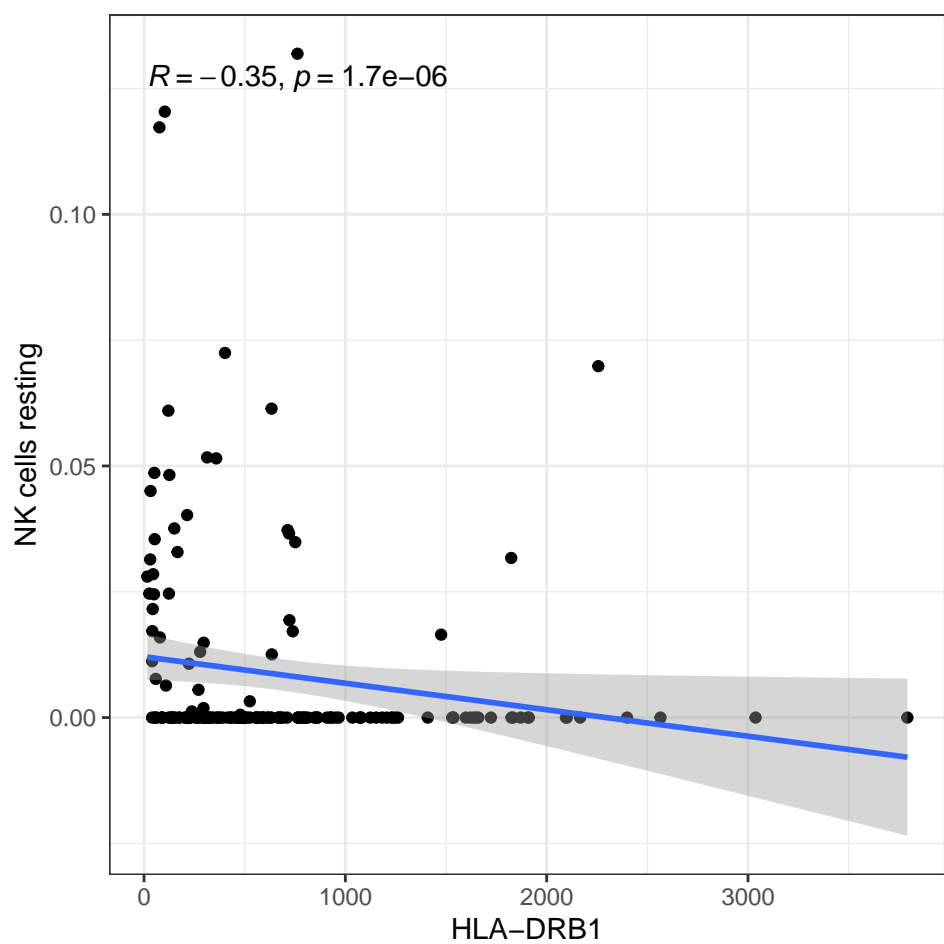

Supplement: S15 File — (ZIP) [file pone.0274897.s017.zip › Step 15.Immune cell differentiation analysis and correlation analysis/output files/NK cells resting.pdf]

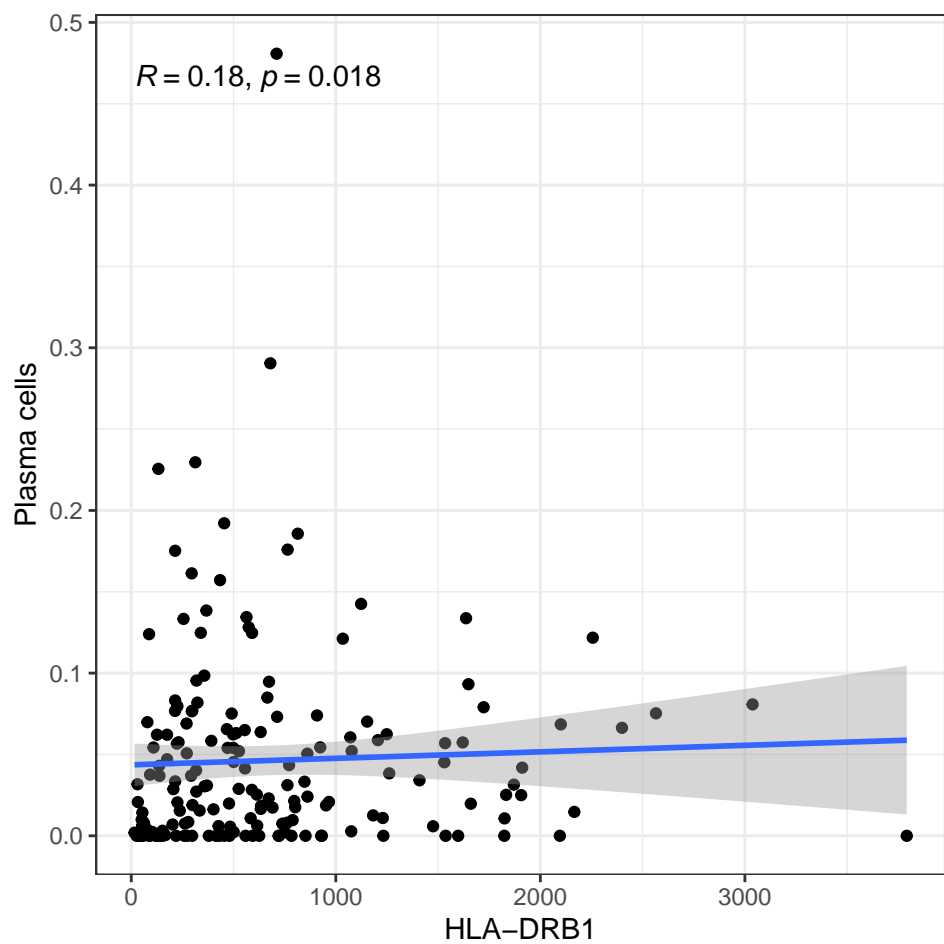

Supplement: S15 File — (ZIP) [file pone.0274897.s017.zip › Step 15.Immune cell differentiation analysis and correlation analysis/output files/Plasma cells.pdf]

T cells CD4 memory activated

$R = 0.35, p = 2.2e-06$

0.15

0.10

0.05

0.00

0

1000

2000

3000

HLA-DRB1

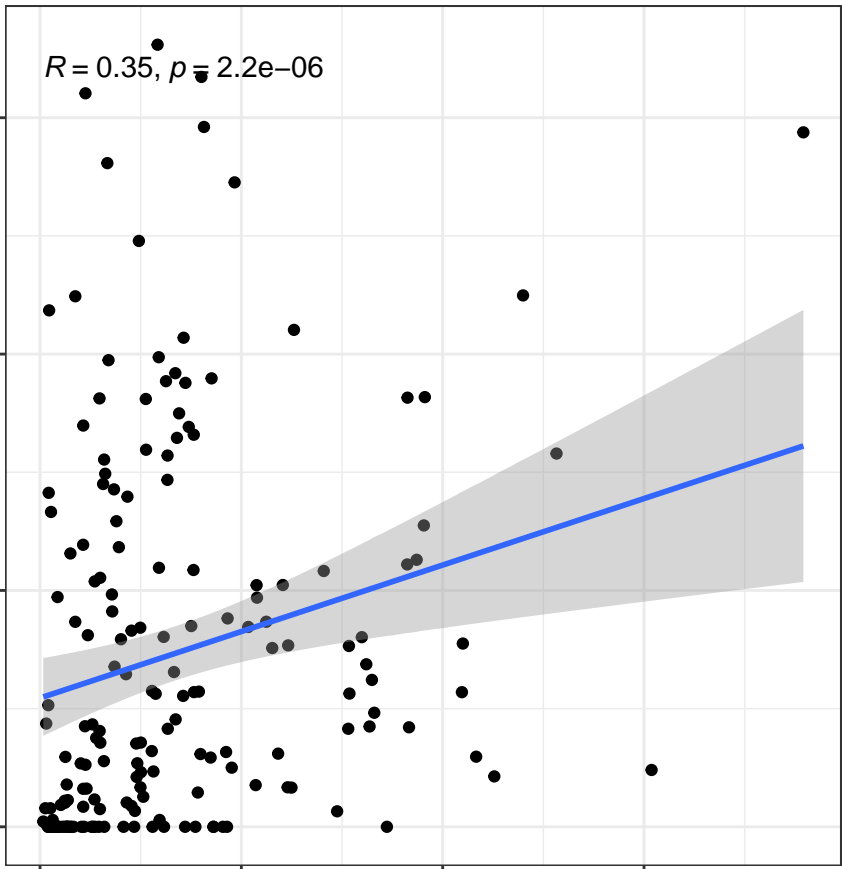

Supplement: S15 File — (ZIP) [file pone.0274897.s017.zip › Step 15.Immune cell differentiation analysis and correlation analysis/output files/T cells CD4 memory activated.pdf]

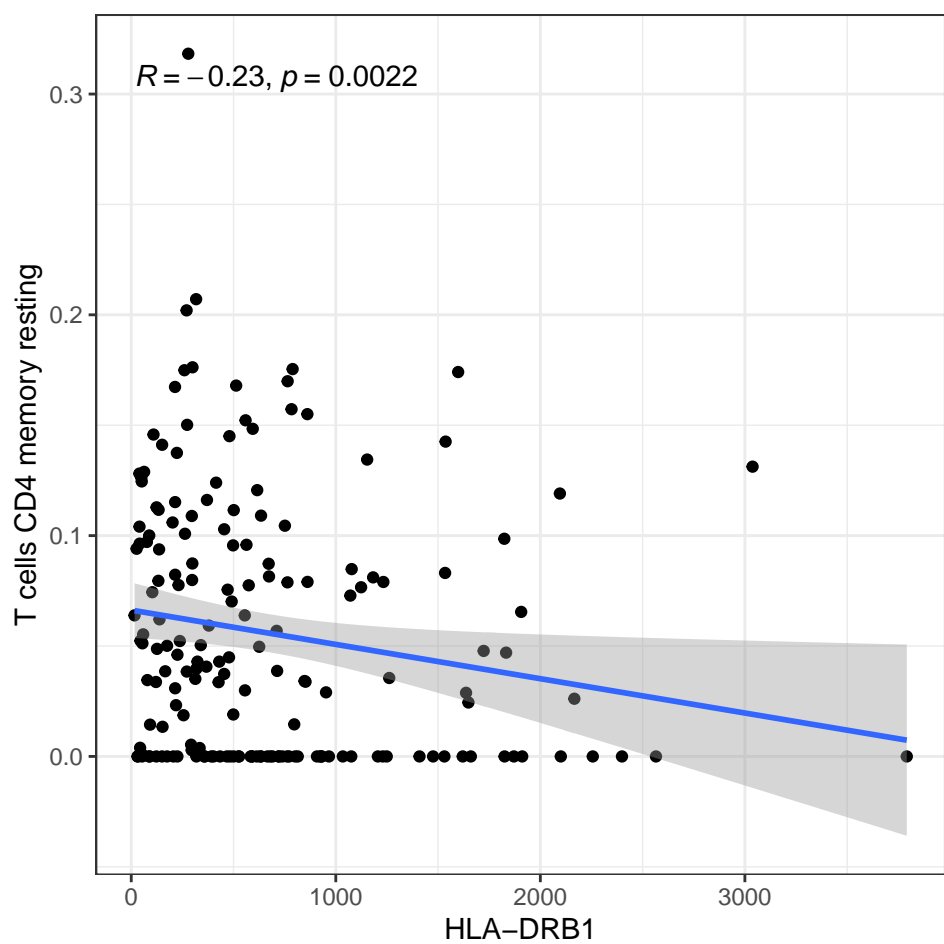

Supplement: S15 File — (ZIP) [file pone.0274897.s017.zip › Step 15.Immune cell differentiation analysis and correlation analysis/output files/T cells CD4 memory resting.pdf]

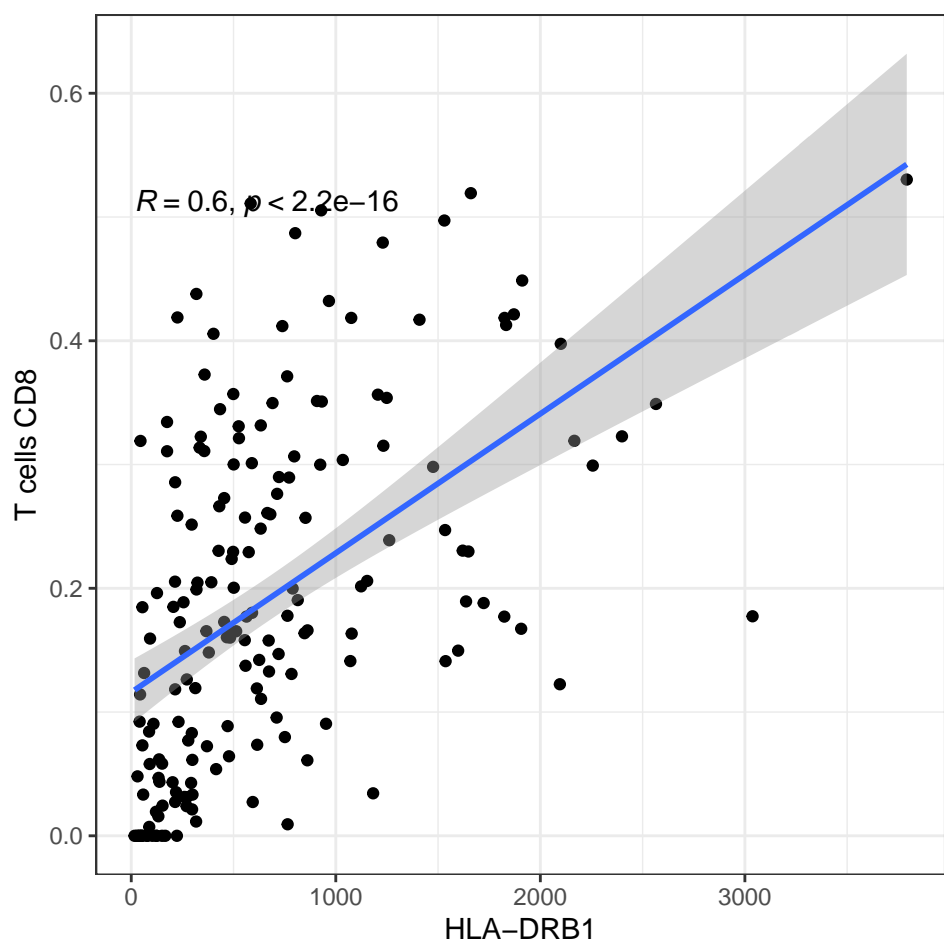

Supplement: S15 File — (ZIP) [file pone.0274897.s017.zip › Step 15.Immune cell differentiation analysis and correlation analysis/output files/T cells CD8.pdf]

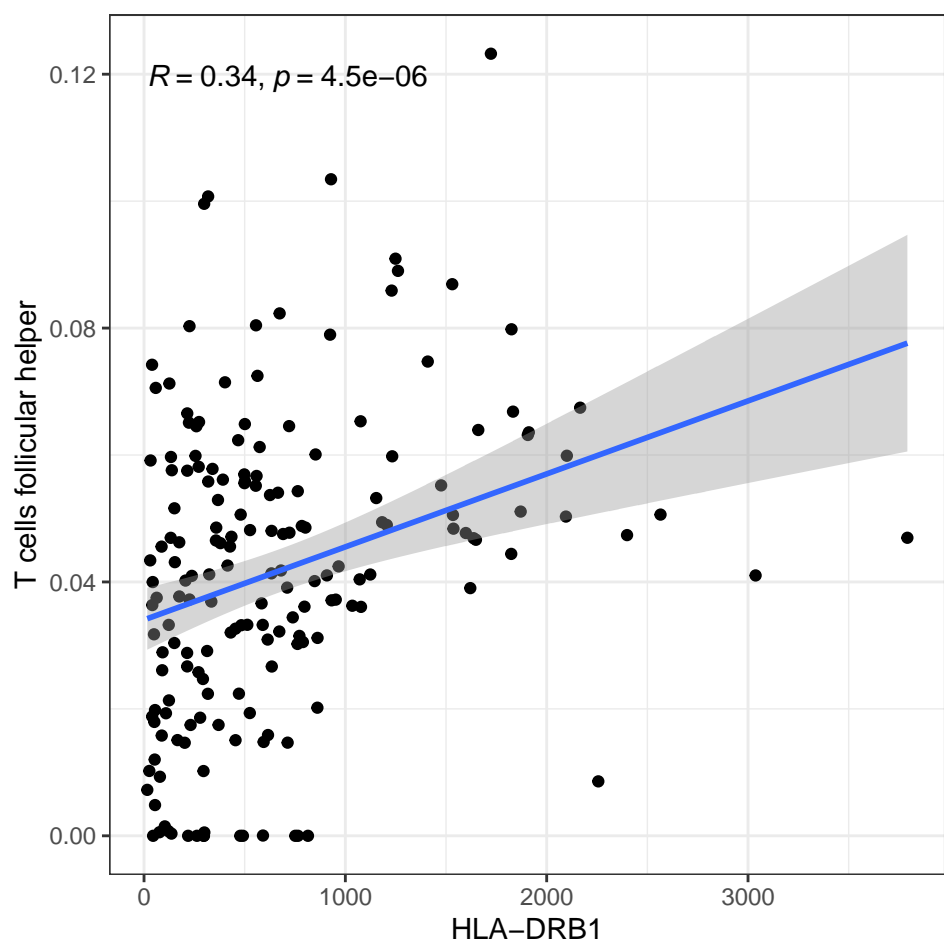

Supplement: S15 File — (ZIP) [file pone.0274897.s017.zip › Step 15.Immune cell differentiation analysis and correlation analysis/output files/T cells follicular helper.pdf]

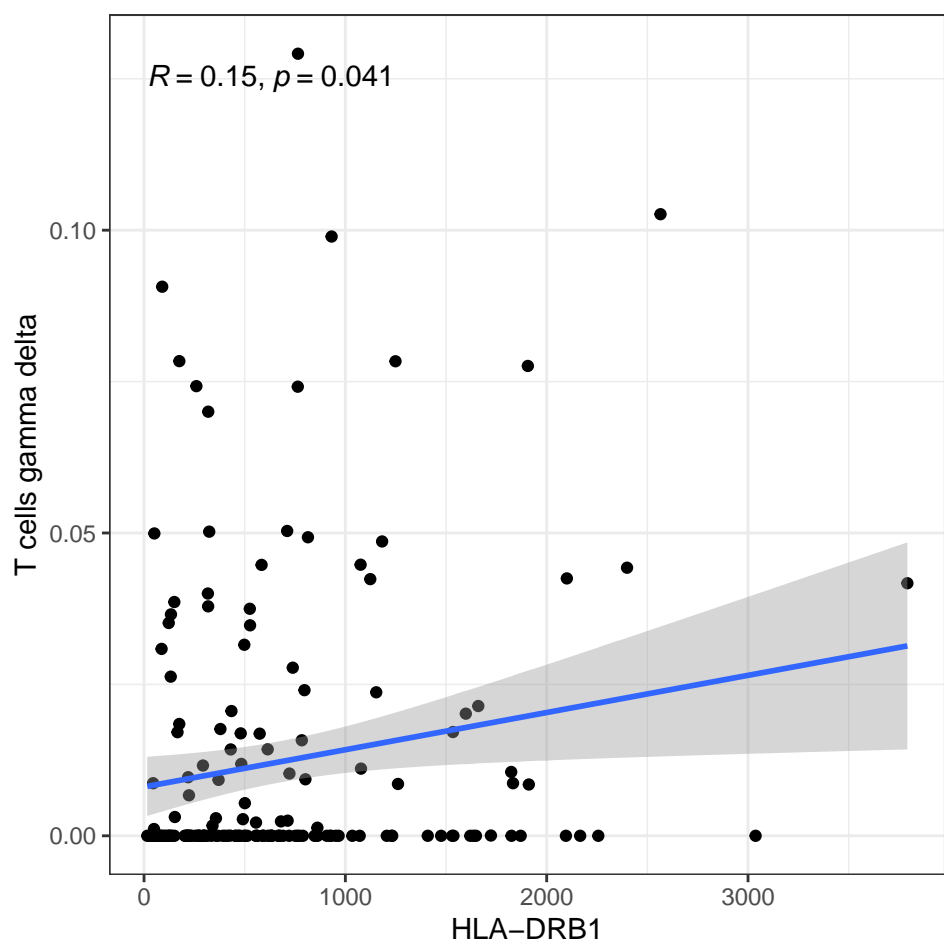

Supplement: S15 File — (ZIP) [file pone.0274897.s017.zip › Step 15.Immune cell differentiation analysis and correlation analysis/output files/T cells gamma delta.pdf]

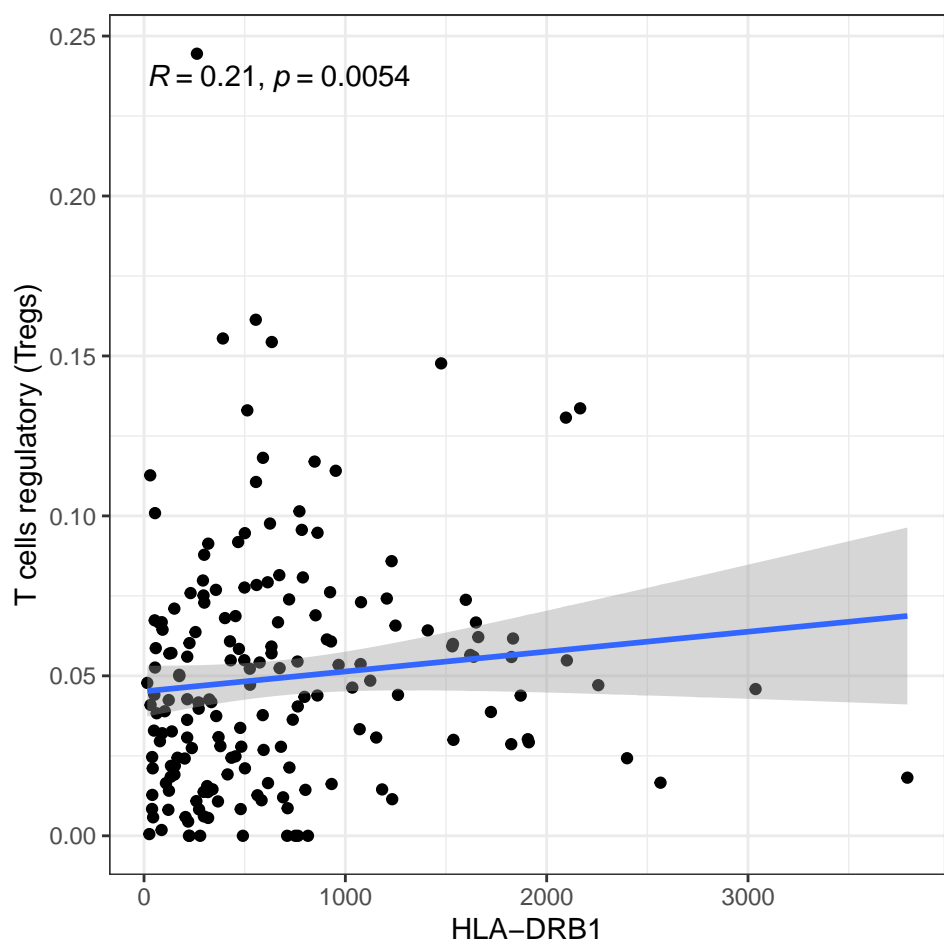

Supplement: S15 File — (ZIP) [file pone.0274897.s017.zip › Step 15.Immune cell differentiation analysis and correlation analysis/output files/T cells regulatory (Tregs).pdf]

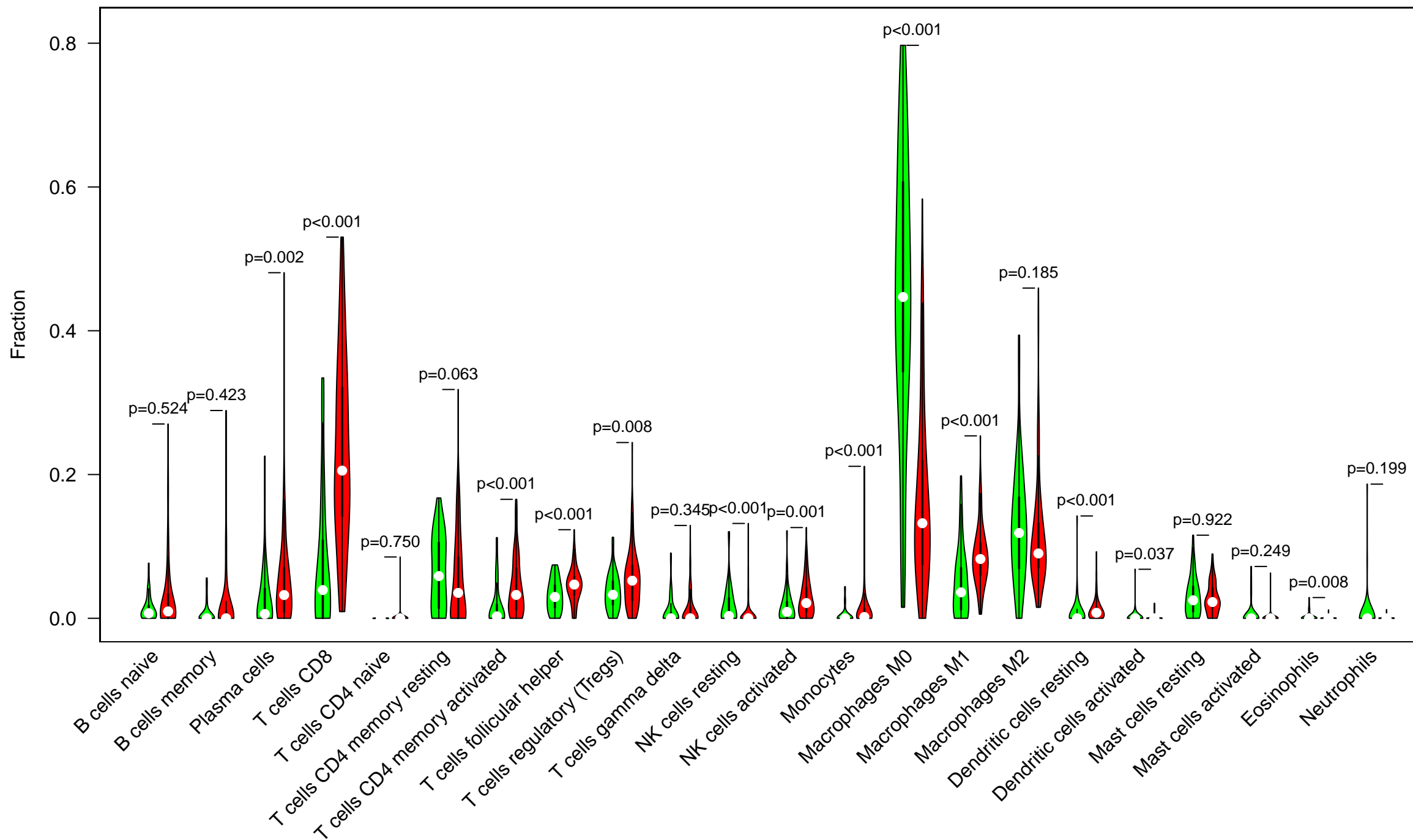

Supplement: S15 File — (ZIP) [file pone.0274897.s017.zip › Step 15.Immune cell differentiation analysis and correlation analysis/output files/vioplot.pdf]

# Intersection

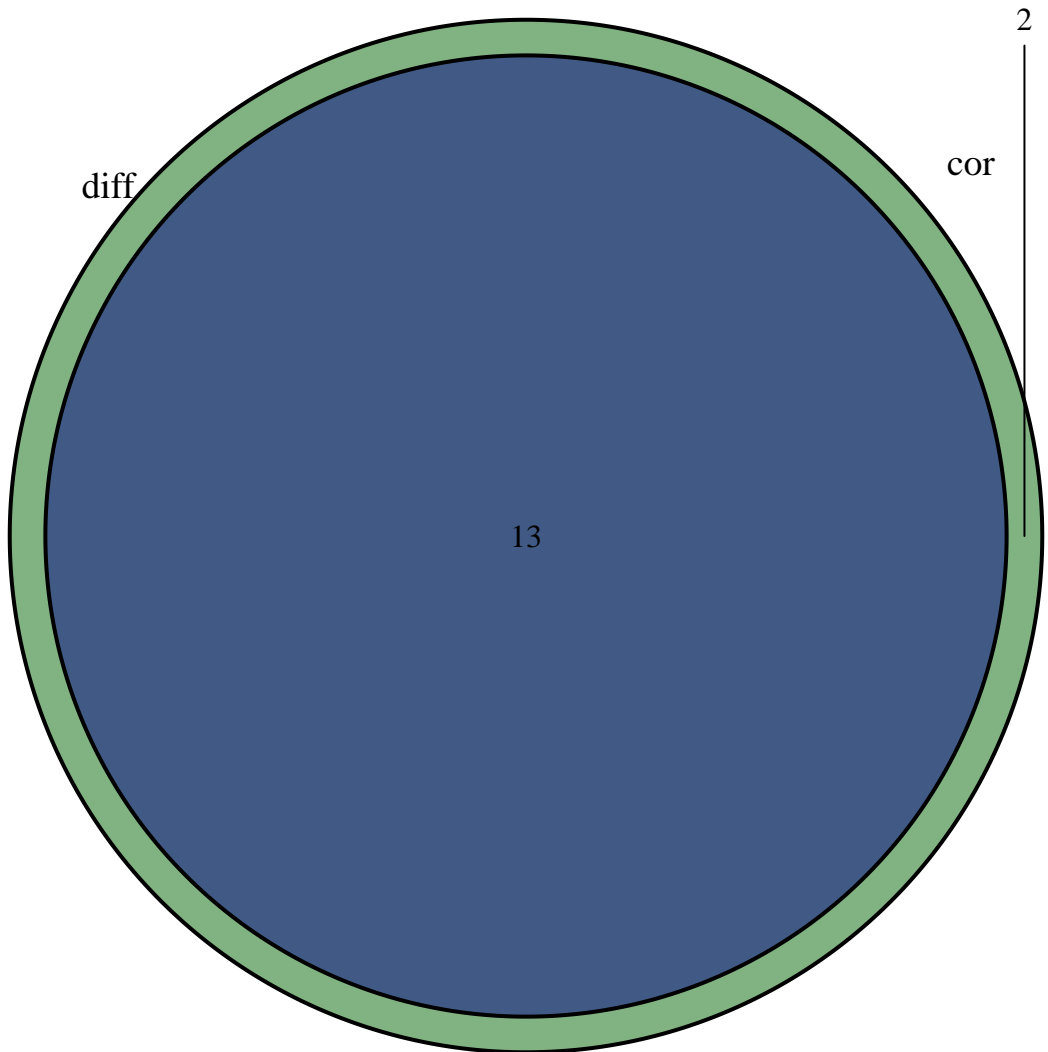

Supplement: S16 File — (ZIP) [file pone.0274897.s018.zip › Step 16.Intersection analysis basing on the result of immune cell differentiation analysis and correlation analysis/output files/immuneVenn.pdf]
